# Palm Fungi and Their Key Role in Biodiversity Surveys: A Review

**DOI:** 10.3390/jof9111121

**Published:** 2023-11-19

**Authors:** Diana S. Pereira, Alan J. L. Phillips

**Affiliations:** Biosystems and Integrative Sciences Institute (BioISI), Faculdade de Ciências, Universidade de Lisboa, Campo Grande, 1749-016 Lisboa, Portugal

**Keywords:** *Arecaceae*, biodiversity surveys, fungal biodiversity, fungal estimates, missing fungi, palm trees, taxonomy

## Abstract

Over the past three decades, a wealth of studies has shown that palm trees (*Arecaceae*) are a diverse habitat with intense fungal colonisation, making them an important substratum to explore fungal diversity. Palm trees are perennial, monocotyledonous plants mainly restricted to the tropics that include economically important crops and highly valued ornamental plants worldwide. The extensive research conducted in Southeast Asia and Australasia indicates that palm fungi are undoubtedly a taxonomically diverse assemblage from which a remarkable number of new species is continuously being reported. Despite this wealth of data, no recent comprehensive review on palm fungi exists to date. In this regard, we present here a historical account and discussion of the research on the palm fungi to reflect on their importance as a diverse and understudied assemblage. The taxonomic structure of palm fungi is also outlined, along with comments on the need for further studies to place them within modern DNA sequence-based classifications. Palm trees can be considered model plants for studying fungal biodiversity and, therefore, the key role of palm fungi in biodiversity surveys is discussed. The close association and intrinsic relationship between palm hosts and palm fungi, coupled with a high fungal diversity, suggest that the diversity of palm fungi is still far from being fully understood. The figures suggested in the literature for the diversity of palm fungi have been revisited and updated here. As a result, it is estimated that there are about 76,000 species of palm fungi worldwide, of which more than 2500 are currently known. This review emphasises that research on palm fungi may provide answers to a number of current fungal biodiversity challenges.

## 1. Introduction

*Arecaceae* (syn. *Palmae*), colloquially known as palm trees, is one of the best known and most extensively cultivated plant families, comprising around 2600 species in 181 genera [1]. Palms are important plants in terms of human exploitation in their native range. Some species, such as oil (*Elaeis* species), coconut (*Cocos nucifera*), rattan (*Calamus* species), and date (*Phoenix dactylifera*) palms, are extremely important in the international trade [2,3,4]. Moreover, palms are highly prized as ornamentals due to their great decorative potential. Thus, although almost exclusively native to tropical or sub-tropical countries, these perennial monocotyledonous trees are currently distributed worldwide due to their use as ornamental plants [4,5]. Therefore, palm trees have become a distinctive component of the urban landscape and an important accessory in interior decoration and in floristry.

Over the last 30 years, a considerable number of studies have shown the association of a diverse range of fungi with palm tissues. The extensive research carried out in Southeast Asia and Australasia showed that palms are a rich source of previously unknown fungal taxa. Thus, many species and genera were formally identified and described as new to science based on palm collections, e.g., refs. [6,7,8]. The comprehensive isolation of fungi from palm tissues has proven that these fungi are an important and taxonomically diverse assemblage that is often referred to as palm fungi or palmicolous fungi, e.g., ref. [9]. While most studies on palm fungi have focused on systematic and descriptive taxonomy, a few studies have explored the biodiversity and ecology of these microfungi, e.g., refs. [10,11,12,13,14,15,16]. Taxonomically, palm fungi are one of the most diverse groups of fungi. The currently established figure is that more than 1500 species of fungi have been described from palm hosts, with representatives from almost all major fungal classes [17]. In earlier studies, most taxa were introduced, described, and arranged in different taxonomic ranks within the *Ascomycota* based on morphological analyses. This approach was, however, subjective and many taxa were wrongly assigned or assigned to the *Ascomycota* genera *incertae sedis*.

Despite the wealth of data on the assemblage of fungi that inhabit palm tissues, there has been no recent comprehensive review of palm fungi to date. In this regard, the present review aims to give a historical overview on the studies that have been performed on palm fungi and reflect on their importance as a diverse and understudied assemblage. Moreover, two main aspects will be presented and discussed: (1) what the taxonomic structure of palm fungi is, along with comments on the need for further studies to place them within modern DNA sequence-based classifications; (2) how palm trees can be regarded as model plants for studying fungal biodiversity, given the key role of palm fungi in biodiversity surveys.

## 2. Historical Account of Research on Palm Fungi and Reflections on Their Importance

The history of the study of palm fungi can be divided into three distinct periods. The first, between 1880 and 1920, includes the publications of classical mycologists, such as Hennings, Rehm, Penzig, Spegazzini, H. Sydow and P. Sydow, and Saccardo [18,19,20,21,22,23,24,25,26,27]. Although many fungal species were described from palm trees during this period, these publications are barely accessible or legible since most of them were written in Latin. The second, between 1920 and 1990, includes several mycologists who, for 70 years, occasionally reported the occurrence of fungi on palm tissues. However, almost no study on palm fungi in this period was very extensive. The third, which began in the 1990s and continues today, represents the first time that a group of mycologists has dedicated itself to specifically analysing the microfungi that occur on palm trees. This includes the research carried out by Hyde, his students, and colleagues, which represents not only a significant advance in the number of fungal species described on palm substrata, but also a considerable advance in the biodiversity of palm fungi.

### 2.1. History of Systematic and Descriptive Taxonomy Studies on Palm Fungi

There are numerous reports scattered throughout the literature on fungi collected from different parts of different species of palm trees and from different regions of the world. An overwhelming number of these studies have been dedicated to collecting and describing fungi that inhabit palm tissues, including new genera and new species. Although there are several reports from before the 1990s, the present overview focuses on the extensive studies carried out by Hyde and co-workers. Hyde’s research is the first body of theory to understand palm fungi as a fungal community with taxonomic characteristics, biological processes, and general diversity that is worth exploring in depth. Most of the descriptions prior to Hyde’s research, especially those from the early 20th century, consist of short Latin paragraphs that lack illustrations and/or give limited information about the identity of the fungi.

#### 2.1.1. From Scattered to the First Systematic Reports on Palm Fungi

**Figure 1:**
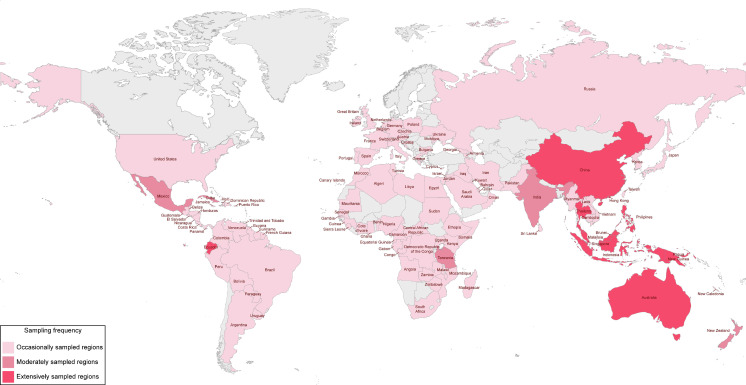
World sampling regions of palm trees for associated fungi based on the available literature. Studies prior to 1950 were not considered when constructing the map, as most of the information is difficult to access. The regions/countries where palm trees were collected are named and delimited with coloured blocks and referred to in the chart legend. Extensively sampled regions are those where most studies have been carried out to specifically analyse palm fungi. Moderately and occasionally sampled regions are those where the reports of palm fungi were a consequence of occasional taxonomic or broader studies. Many occasionally sampled regions have not been mapped, as they are island countries that are barely visible due to the scale of the map. These include several Antilles islands of the Caribbean (Barbados, Dominica, Grenada, Guadeloupe, French West Indies, and Virgin Islands), Bermuda, African island countries (Comoro Islands, Mauritius, São Tomé and Príncipe, Réunion, and Seychelles), the Andaman and Nicobar Islands, and several islands in the Pacific Ocean (Cook Islands, French Polynesia, Niue, Samoa, and Tonga in Polynesia, Fiji, Vanuatu, and Solomon Islands in Melanesia, and Guam and Kiribati in Micronesia, Oceania). Figure source: created with a template available in the webserver for MapChart (https://www.mapchart.net/, accessed on 15 September 2023).

Before the extensive studies carried out by Hyde and co-workers, there were several occasional reports of fungi collected from different palms and tissues, such as leaf litter, green foliage, rachides, trunks, and stems. Although these collections come from different countries around the world, most of them come from tropical and subtropical regions where palm trees are almost exclusively native. A summary of some examples of these studies is given here considering the last decades of the 20th century. The sampling regions around the world where palm fungi have been recorded are mapped in Figure 1, according to the available literature.

Collections of palm species native to tropical regions of Central and South America and West and Southeast Africa (Figure 1) have often yielded taxonomic novelties, including from new genera and species to new records on new hosts and new geographical distributions. For instance, Ellis [28] reported new *Lacellina* species on dead leaves of *Borassus aethiopum* from Ghana and Sierra Leone (West Africa). Later, Ellis also reported new species of palm fungi from Central America, West Africa, and Malaysia on his series of papers and books concerning “Dematiaceous Hyphomycetes” [29,30,31,32].

Southeast Asia, Australasia, and India were also frequently surveyed (Figure 1) and the first two would later become major regions for palm fungi investigation. For instance, while studying leaf-spotting hyphomycetes, Deighton [33,34] reported the new species *Cercospora raphiae* and *Pseudocercospora carpentariae* on leaves of *Raphia farinifera* from Zimbabwe (Southeast Africa) and *Carpentaria acuminata* from Australia. In the same year, several *Xylariaceae* palmicolous fungi were recorded on the rainforests of North Sulawesi (Indonesia) by Rogers et al. [35].

Several other regions were also only occasionally surveyed (Figure 1), including, for instance, Venezuela (South America), where some palmicolous fungi, including new species, were described in the series of papers “Fungi venezuelani” [36,37,38]; Argentina (South America), where Wright [39] recorded the new genus and species *Agaricostilbum palmicola* (currently synonymised under *Sterigmatomyces pulcherrimus*) on weathered spathes of *Butia yatay* and *Phoenix* sp.; and Japan (East Asia), where Hino and Katumoto [40,41,42] described some palmicolous fungi, including new species, in the series of papers entitled “Notes on fungi from western Japan”. Less frequently, the USA, European countries, and China, where only a few palm species are native, have also been the stage for collecting palm fungi (Figure 1). For instance, several helicosporous fungi collected from palm tissues were reported from Hawaii (USA) by Goos [43]; the new phialosporus hyphomycetes genus and species *Craspedodidymum elatum* were reported on rotten petioles of *P. canariensis* from Czech Republic (Central Europe) by Holubová-Jechová [44]; and the new species *Zasmidium caryotae* (as *Stenella caryotae*) have been reported on the leaves of *Caryota mitis* from Guangdong (China) by Liu and Liao [45].

These first scattered reports of palmicolous fungi have established their importance as a highly diverse and understudied community that can be accessed on palms all over the world (Figure 1). Some reports have expanded the geographical distribution of these fungi, which was important for the establishment of the geographical and ecological range of some important phytopathogens. For example, Samuels and Rossman [46] during their studies on the *Amphisphaeriaceae*-recorded *Leiosphaerella cocoes* on fronds and fruits of *Cocos nucifera* from several different regions, namely Dominican Republic, Guam, Indonesia, Mexico, USA, Tonga, Cook Islands, and Papua New Guinea, and reported two new species of *Oxydothis*, *O. rhopalostylidis* and *O. selenosporellae*, on the leaf midrib of *Rhopalostylis sapida* from New Zealand. It is worth mentioning that New Zealand was one of the first countries where several reports of palmicolous fungi were published more systematically, most of them from collections of the endemic palm tree *R. sapida* (nikau palm), which is the only palm native to mainland New Zealand. Thus, several palm fungi, including many new species, were published in the series of papers “New Zealand Fungi” by Hughes [47,48,49,50,51,52,53], as well as in many other occasional publications that were mostly later summarised by McKenzie et al. [54] in their checklist of fungi recorded on nikau palm from New Zealand.

Two of the first most extensive studies on palm fungi include those of Pirozynski and Matsushima. Pirozynski [55] reported forty-six species, including the new genus *Bondiella*, six new species, and some new combinations of fungi from the oil palm *E. guineensis* collected from Tanzania. Matsushima [56,57,58,59,60,61,62,63,64,65,66,67] and Matsushima and Matsushima [68,69] reported more than 300 fungi from palm litter, including 8 new genera, viz. *Apogaeumannomyces* [67], *Atrosetaphiale* [65], *Hyalobelemnospora*, *Paradactylella* [64], *Setophiale*, *Venustocephala* [65], *Veramyces* [64], *Verticimonosporium* [56], and more than 80 new species, mostly of rare and interesting hyphomycetous fungi. Most of these fungi were recorded in collections of palms from different regions of the world, from the Americas to Australia and Eastern Asia, including Peru, Guam, Taiwan, Cuba, Northern Queensland, and Ecuador (Figure 1), were compiled in the classic book series “Matsushima mycological memoirs”.

Castañeda-Ruiz, Holubová-Jechová, Mena-Portales, and Mercado-Sierra were one of the first groups of mycologists to report several species of palm fungi growing on dead and decaying palm tissues, such as trunks, rachides, and petioles. Although these reports were abundant and consistent, their main purpose was not to report on the palmicolous fungi themselves, i.e., to understand the taxonomic or ecological structure of palm fungal communities. They were part of an investigation of hyphomycetes of Cuba, where many new species were introduced, e.g., refs. [70,71,72,73,74,75,76,77,78,79,80,81,82,83,84,85]. Most of these reports were summarised and discussed in series of papers, such as “Hifomicetes demaciáceos de Cuba” [86,87], “Studies on hyphomycetes from Cuba” [88,89,90,91,92,93,94,95], and “Nuevos o raros hifomicetes de Cuba” [96,97,98,99,100,101,102], and books, such as “Hifomicetes demaciáceos de Sierra del Rosario, Cuba” [103]. Although several of these reports were from undetermined palm trees and other particular known species, most of them were from the Cuban royal palm *Roystonea regia*, where several new species were described, insomuch that Mercado-Sierra [73] has described *R. regia* as an “ideal substratum for the development of dematiaceous hyphomycetes”.

#### 2.1.2. Hyde and Co-Workers and the Extensive Studies on Palm Fungi from Tropical Regions

The last 30 years has seen an extensive profusion of studies regarding palmicolous fungi by Hyde and co-workers. The extent and depth of their investigation yielded an impressive body of literature that made it possible to begin to understand and characterise the taxonomy of palm fungi, particularly the communities inhabiting tropical and subtropical palms growing on their native regions. These systematic studies culminated in the publication of three books entitled “Genera of ascomycetes from palms” [104], “Palm microfungi” [6], and “Microfungi of tropical and temperate palms” [8], and a series of publications entitled “Fungi from palms”, comprising 49 papers where numerous new fungi to science were described [105,106,107,108,109,110,111,112,113,114,115,116,117,118,119,120,121,122,123,124,125,126,127,128,129,130,131,132,133,134,135,136,137,138,139,140,141,142,143,144,145,146,147,148,149,150,151,152,153]. Genera that have been described as new to science and found on palm trees over the last three decades are summarised in Table 1. Genera and respective families in subclasses of *Dothideomycetes* and *Sordariomycetes* with common representatives found on palm trees are summarised in Table 2.

Hyde et al. have been studying microfungi from palms since 1988 when new species of the genera *Linocarpon*, *Oxydothis*, and *Astrosphaeriella* (as *Trematosphaeria*) were described from the fronds of the mangrove palm *Nypa fruticans* from Brunei [154,155,156], during their investigations concerning tropical marine mangrove fungi on three particular regions, i.e., the Indian Ocean (Seychelles), the Straits of Malacca (North Sumatra, Indonesia), and the South China Sea (Brunei) [157,158,159]. These early studies were perhaps the driving force and what set up the research on palm fungi in the 1990s. After those first three taxonomical novelties, numerous further reports of new taxa were made on fronds of intertidal *N. fruticans*, predominantly from Brunei, e.g., refs. [105,110,117,131,160,161,162,163,164,165] and Malaysia, e.g., refs. [116,143,166,167], which yielded more than thirty new species and seven new genera (Table 1).

Nipa palm was found to be a “very distinct mangrove habitat” for fungal surveys, where a “largely distinct mycota” was found, including several “unique marine species belonging to genera consistently found on terrestrial palms” [168]. Hyde [110] observed that *Astrosphaeriella*, *Linocarpon*, and *Oxydothis*, which are genera typically associated with terrestrial palm petioles in the tropics, were often associated with decaying intertidal Nipa palms. Not only was this mangrove palm tree important for systematic and descriptive taxonomy studies, but it was also a very important substratum for some of the first studies on the ecology of palm fungi, e.g., ref. [7], as will be discussed later. Although most studies focused on the intertidal fungi occurring on *N. fruticans*, studies have also been conducted in order to access the fungi inhabiting the aerial parts of this mangrove palm [165].

Perhaps encouraged by those early records, during the last decade of the 20th century, Hyde et al. carried out an extensive survey of palmicolous fungi from a wide range of tropical palm species in different regions of the world (Figure 1). The tropical regions of Southeast Asia and Australasia comprised the countries in which most of the collections were made, such as Brunei [113,115,116,117,133,134,138,154,155,160,161,162,163,164,169,170,171], Indonesia [109,113,115,124,126,131,133,134,172], Malaysia [110,116,117,118,123,131,133,134,165,167,172], and Australia [108,109,112,114,117,133,135,138,172]. Other regions and countries were moderately or occasionally sampled, including Thailand [162], Papua New Guinea [117,123,127,131,133,173,174,175], Philippines [117,125,167], Japan [117,133], Ecuador [128,138,171], Brazil [107], and the USA [122,124]. All the regions surveyed revealed the presence of an enormous diversity of fungi, among which, 22 genera and more than 80 species were described as new to science, several existing genera were re-examined, and some new combinations were proposed. In fact, one of the most remarkable outcomes of these early works by Hyde was the number of new genera introduced (Table 1). Not only did they reveal the great untapped diversity of the fungal communities that inhabit palm trees in the tropics, but they also discovered a very particular unknown and underexplored taxonomic group of ascomycetes which make up what has been termed palm fungi (Table 2). Therefore, this comprehensive research began to unveil and shape the community of palmicolous fungi, insomuch that it allowed to build up and consolidate the knowledge on the common mycota that typically inhabit the tissues of tropical and subtropical palms. According to Hyde [111], fallen palm rachides and leaves in the tropics were found to be invariably colonised by fungi of the genera *Astrosphaeriella*, *Linocarpon*, *Oxydothis*, and *Phomatospora*. It later became clear that, in addition to *Oxydothis*, several other genera of *Xylariales* had common representatives on palm trees, including *Anthostomella*, *Apioclypea*, *Arecomyces*, *Astrocystis*, *Capsulospora*, *Fasciatispora*, *Nipicola*, and *Pemphidium* [134,138] (Table 2).

Along with Hyde’s work on clarifying, redescribing, illustrating, and monographing existing genera [105,107,111,113,117,124,126,127,130,133,171], these studies resolved certain taxonomic relationships within various important fungal families and orders. Moreover, they also expanded the knowledge of tropical mycology, especially with regard to the biodiversity of fungi that inhabit tropical hotspots, such as rainforests. Striking examples include the description of numerous new species in the genera *Linocarpon* [105,154,165,172], *Oxydothis* [111,112,117,156], and *Anthostomella* [133,175], three of the most common genera found on palms (Table 2); the clarification of certain unclear generic concepts for some of the genera, and their corresponding species, found on palms, including *Pemphidium* [107,111,135], *Guignardia* [126], and *Roussoella* [171,176]; the description and discussion of some amphisphaeriaceous fungi occurring on palms, such as the genera *Fasciatispora* [124,161], *Myelosperma* [113], *Seynesia* [127], *Arecophila* [131], and *Amphisphaeria* [136]; and the investigation of palmicolous fungi inhabiting the palms in tropical Australian, e.g., refs. [112,114], Bruneian, e.g., refs. [170,174], and Ecuadorian, e.g., refs. [128,171] rainforests. These first case studies in the early 1990s verified the existence of a well-represented set of morphological characters to describe these genera, as well as to distinguish them from other related genera, and to clarify the existence of new morphologically similar genera. As a result, it began to become clearer what the taxonomic placement of palm fungi was in higher taxonomic ranks, including different families of the *Xylariales* and other less representative orders, e.g., ref. [138] (Table 2).

The large number of new genera and species reported on palms by Hyde in the early 1990s quickly prompted a growing interest in the mycobiota of these hosts and several of his students and collaborators, such as Fröhlich, Taylor, Aptroot, and Goh, worked to expand the knowledge of palmicolous fungi. Thus, in addition to the many new taxa recorded, different aspects of these fungi have been studied and have contributed to the fundamental knowledge of fungi and their biodiversity. This intensive research ultimately led to the description of at least three new families to accommodate genera that have been described and found to be common on palms, along with extant related genera, namely *Phaeochoraceae* for *Cocoicola*, *Phaeochora* and *Serenomyces* [177], *Apiosporaceae* for *Apiospora* and *Appendicospora* [143], and *Myelospermaceae* for *Myelosperma* [178].

Hyde, Fröhlich, Taylor, Aptroot, and Goh, studying ascomycetes developing on living, diseased, and dead palm material, surveyed different regions from East, South, and Southeast Asia, including, respectively, China (Hong Kong, Hubei, Hunan) [6,8,129,140,142,143,144,146,147,148,149,151,179,180,181,182,183,184,185,186], India [152], and Brunei, Indonesia, Laos, Malaysia, Philippines, and Singapore [6,8,122,139,140,143,147,148,149,150,153,179,181,185,187,188,189,190,191,192,193]. The Australasian regions were also intensively surveyed, including Australia [6,8,121,140,141,143,144,147,148,181,185,187,189,194,195,196,197,198,199,200,201,202,203,204] and Papua New Guinea [150,198]. Other regions and countries were also frequently or occasionally surveyed, including Ecuador (South America) [6,119,143,144,150,179,181,185,205,206,207,208], USA [120], South Africa [208,209,210], Seychelles [8,178,181], and European countries, such as Switzerland and Great Britain [8] (Figure 1). As expected, the huge diversity of fungi collected from palm trees increased, with 22 new genera and more than 200 new species described. As a result, the importance of palm fungi began to become more evident as studies continuously revealed their broad taxonomic structure (Table 2). Most of these studies and their outcomes have been compiled in the first book published by Hyde and co-workers in the Fungal Diversity Research Series concerning palmicolous fungi, which was entitled “Genera of ascomycetes from palms” and treated 100 selected genera of common fungi inhabiting the tissues of tropical palms [104]. Moreover, another book on the same series, entitled “Palm microfungi”, has been published in the same year, which supplemented the previous information describing the ascomycetes found on palms during their comprehensive collections in Australia, Brunei, and Hong Kong [6]. Remarkably, in addition to the ongoing revision of several genera of ascomycetes with common representatives on tropical palms, Fröhlich and Hyde [6] described 65 taxa as new to science, including 3 new genera and an impressive number of 23 new species of *Oxydothis*, considered to be the genus most commonly found on palms and invariably one of the earliest colonisers of dead palm leaves and fronds [185,211] (Table 1 and Table 2).

Following Hyde’s early studies of palm fungi in tropical rainforests, e.g., refs. [112,170,171], one intensively studied region worth mentioning is the rainforests of North Queensland (Australia) (Figure 1), where an immense diversity of fungi has been found in collections of different endemic palm species, such as *Archontophoenix alexandrae*, *Laccospadix australasica*, *Licuala ramsayi*, *Linospadix microcaryus*, *L. monostachyos*, and *Oraniopsis appendiculata*, e.g., refs. [121,140,141,143,145,147,148,181,185,187,189,194,195,196,197,198,199,200,202]. While most of the studies conducted by Hyde and co-workers were dedicated to surveying saprobic fungi that develop on palm trees, some of these studies in the North Queensland rainforest were conducted to survey palm phytopathogens, particularly those parasitic on palm leaves and causing leaf spot diseases. Palm phytopathogens were also documented in studies conducted during an expedition of the British Mycological Society to Ecuador in August 1993 to gather fungi developing on palms in the rainforest of Cuyabeno [119,143,144,150,181,185,206,207,208]. Moreover, Hyde and Cannon [212] monographed members of the *Catabotrydaceae*, *Phaeochoraceae*, and *Phyllachoraceae* families that occur in association with tar spots on palms. In addition to treating these families and their placement in the fungal classification, several genera were treated and analysed, some were re-introduced based on palm collections, and four genera and three species were described as new to science (Table 1). Some of the genera discussed by Hyde and Cannon [212] represent important taxa in the phytopathogenic mycobiota of palm trees, such as the genus *Serenomyces*, a group of mostly biotrophic fungi apparently known only in association with members of *Arecaceae* [213,214].

Although some palm phytopathogens have been identified, most studies on palmicolous fungi in the last decade of the 20th century focused on the myriad of saprobic fungi that inhabit the palms from tropical rainforests, where palm litter is a major component. Samples were collected from many different palm trees that inhabit the tropics, including from typical *Phytelaphas* spp. of Central and South America to typical *Licuala* spp. of Southern China (Figure 1). Palm material, such as senescent and dead petioles, rachides, stems, fronds, and decaying and dead trunks, yielded plentiful novelties (Table 1). One interesting palm tree worth mentioning is *A. alexandrae*, an endemic palm from Queensland, Australia. Studies on the palmicolous fungi that inhabit the tissues of this palm have perhaps been the driving force for studying other questions about the taxonomy and ecology of palm fungi, as well as extending the research to fungi associated with palms from non-tropical habitats. In fact, its endemic nature and relative geographic isolation make *A. alexandrae* an ideal substratum for studying host-specificity and fungal biogeography, which have been identified as important factors for the description of many novel palmicolous fungi when hosts are studied in their natural environment [142,194]. Likewise, the description of the new palmicolous genus *Cannonia* [204] collected from *Trachycarpus fortunei* in Australia, outside its native temperate range in parts of China and some neighbouring countries (Table 1), also raised questions about the importance of studying the biogeography of palm fungi. As a result, studies on palmicolous fungi that expanded their geographical boundaries and temperate palms also began to be surveyed [215].

The constant description and illustration of new taxa found on palm trees has often led to discussions of their placement in the fungal classification. As a result, several families, and their respective genera, with common representatives on palms, have been extensively treated and the taxonomy of palmicolous fungi, at least in the tropics, has become clearer, e.g., refs. [6,8,104,143,150,171,178,181,185,212,216,217] (Table 2). Noteworthy examples include the description and discussion of common genera that occur on palms with the introduction of several new species, such as *Nipicola* [137], *Nectria*, and allied genera [207], *Massarina* [139,149], *Anthostomella* [143,209,218,219,220,221], *Astrosphaeriella* [144,148], and *Neolinocarpon* [140], whose geographical distribution has widened considerably throughout Australasia, Southeast Asia, South America (Ecuador), and China (including Hong Kong). Other common genera discussed, with the introduction of new species, include *Dictyosporium* [184] and *Lasiosphaeria* and similar genera, such as *Chaetosphaeria*, *Iodosphaeria*, and the newly described genus *Arecacicola* [146,181,185] (Table 1). Likewise, some doubtful species described for some genera that occur on palms have been clarified and revised, namely in *Mycosphaerella* and *Sphaerella*, which are common phytopathogens [145], *Didymosphaeria* and similar taxa, which include truly terrestrial ascomycetes with ascospores with appendages described from palms [147,222,223], and genera of unitunicate ascomycetes with apiospores, which are frequently recorded on palms, such as *Anthostomella, Apioclypea*, *Apiospora*, *Appendicospora*, and the new described genera *Brunneiapiospora* and *Palmaria* (as *Palmomyces*) [143] (Table 1). Several xylariaceous genera recorded on palms have also been treated based on herbarium specimens and fresh material collected during the investigation on palmicolous fungi, which led to the description of several new species, for example, of the genus *Astrocystis* [150].

Following the incredible contribution to the knowledge of fungal biodiversity and their taxonomy, these studies have also made it possible, on several occasions, to discuss and even uncover some links between sexual and asexual morphs [150,167,181,203,208,224]. The importance of studying “anamorph-teleomorph” connections was strongly emphasised in the last compilation book published by Taylor and Hyde [8] in the Fungal Diversity Research Series on palm fungi, entitled “Microfungi of tropical and temperate palms”. As well as continuing the previous studies on palm fungi carried out on tropical rainforest palms, Taylor and Hyde [8] also studied fungi associated with palms in non-rainforest habitats in the tropics and palms in temperate habitats. Interestingly, this is the first book to deal not only with the identification of palmicolous fungi but also with the extent of their diversity and the factors that affect it, which reflects the complexity of the investigation carried out by Hyde and co-workers and the diversity of the approaches that were used. A total of thirty-four new species, including four new species of *Anthostomella*, and one new genus, *Tribulatia*, were described (Table 1), which is a surprising number considering the extent of the sampling and the number of taxa already described from palms by Hyde and co-workers.

The studies on palmicolous fungi in tropical regions continued, with other regions beginning to be surveyed more systematically, including many reports from additional Hyde co-workers, namely McKenzie, Pinnoi, Pinruan, and Yanna. Although the description of new taxa remained the main objective of these studies, which yielded more than thirty species and seven genera described as new to science (Table 1), they have also begun to provide ecological data on the communities of tropical palmicolous fungi that inhabit some tropical palm tree species, such as *Livistona chinensis* and *Phoenix hanceana*, e.g., refs. [13,14,15]. Yanna et al. [224,225,226,227,228,229] described several new species from different palm tree species in Hong Kong, comprising the typical *Ascomycota* assemblage commonly found on palms, particularly species of *Appendicospora* [225], to atypical hyphomycetes and coelomycetes genera, such as *Koorchaloma* [226], *Staurophoma* [224], *Endomelanconium* [227], and *Everhartia* [229].

Although the initial studies on palms focused mainly on the evaluation of the *Ascomycota* coverage through the presence of its sexual morphs on the host, the evident potential of these hosts for biodiversity surveys has led to a diversification of approaches and, consequently, discoveries. As a result, a considerable number of reports began to describe several new species and genera of palmicolous hyphomycetes, particularly dematiaceous hyphomycetes, in addition to the usual sexual morphs of ascomycetes from well-studied regions, such as Australia (North Queensland) [15,189,201,202,230], China (Hong Kong and Hainan) [182,183,184,231,232,233,234,235], Brunei, and Thailand [166,188,189,190,228,236,237,238] (Figure 1). In addition to hyphomycetes, species of the discomycetes genus *Lachnum*, new species of which are often found on palm trees [239,240], have also been reported in tropical China, e.g., ref. [231].

Just before the input of molecular data began to broaden and strengthen the research of Hyde and co-workers on palm fungi, McKenzie, Pinnoi, and Pinruan et al., studying the fungal diversity on palms from the Sirindhom Peat Swamp Forest at Narathiwat, Southern Thailand, found and described several new palmicolous taxa [236,241,242,243,244,245,246,247]. Thus, in addition to new species of the typical *Ascomycota* assemblage reported from palm tissues in the tropics, including new species of *Submersisphaeria* [247], *Jahnula* [242], and the new genus *Unisetosphaeria* [245] (Table 1), some new palmicolous “anamorphs” were introduced, including new species of *Chalara* [241], *Dactylaria* [245], *Custingophora*, *Vanakripa* [246], *Craspedodidymum* [243], and *Stachybotrys* [244]. Moreover, following the studies carried out by Yanna et al., ecological data on tropical palm fungal communities have also been documented in the peat swamp palms *Eleiodoxa conferta* [248] and *Licuala longicalycata* [249], as well as in *Calamus* spp. [16].

#### 2.1.3. The Palmicolous Hyphomycetes from Central American Countries

A perusal of the available literature on palm fungi reveals that, to date, no intensive studies have been carried on palmicolous “anamorphs”, i.e., hyphomycetes and especially coelomycetes. However, it is worth mentioning a few scattered studies that reported new species and genera of palmicolous hyphomycetes. These studies have shown that the predominant group of “anamorphs” in palm litter are the dematiaceous hyphomycetes. In fact, one of the first consistent reports of palmicolous hyphomycetes is that of Ellis in the 1960s and 1970s, who reported several new species and some new combinations based on collections of palms from West Africa, Southeast Asia, and Central America in his series of papers and books about “Dematiaceous hyphomycetes” [29,30,31,32,250,251,252,253,254,255,256,257,258].

**Table 1:** Genera described as new to science and found on *Arecaceae* hosts in the last three decades.

Genus	Type Species	Host	Country/Region	Sequence Data ^1^	Reference
*Acarocybellina*	*A. arengae*	On a dead leaf of *Arenga engleri*	Japan	N/A	[259]
*Acarocybiopsis*	*A. cubitaensis*	On a dead trunk of *Roystonea regia*	Cuba	N/A	[260]
*Acuminatispora*	*A. palmarum*	On decaying petioles and rachides of an unidentified palm in mangrove	Thailand	A	[261]
*Agrabeeja*	*A. kavakapriya*	On synnemata of *Melanographium citri* on a rachis of *Korthalsia grandis*	Singapore	N/A	[262]
*Allodiatrype*	*A. arengae*	On a dead petiole of *Arenga pinnata*	Thailand	A	[263]
*Anabahusakala*	*A. amapensis*	On decaying leaves of *Syagrus* sp.	Brazil (Amapá)	N/A	[264]
*Anisospadicoides*	*A. macrocontinua* (as *Spadicoides macrocontinua*)	On a rotten petiole of an unidentified palm	Peru	N/A	[64,265]
*Apioclypea*	*A. livistonae*	On a rachis of *Livistona* sp.	Papua New Guinea	N/A	[175]
*Apogaeumannomyces*	*A. perplexus*	On a decaying frond of an unidentified palm	Peru	N/A	[67]
*Appendicospora*	*A. coryphae*	On dead rachides of *Corypha elata*	Philippines	N/A	[125]
*Appendispora*	*A. frondicola*	On a dead rachis of *Oncosperma horridum* on forest floor	Brunei	N/A	[115]
*Arecacicola*	*A. calami*	On a trunk of *Calamus* sp.	Indonesia (Java)	N/A	[185]
*Arecomyces*	*A. frondicola*	On a rachis of *Arenga undulatifolia*	Brunei	N/A	[138]
*Arecophila*	*A. gulubiicola*	On a dead trunk of *Gulubia costata*	Papua New Guinea	N/A	[131]
*Ashtaangam*	*A. Sundaram*	On a rachis of an unidentified palm	Malaysia	N/A	[266]
*Astrosphaeriellopsis*	*A. bakeriana*	On a petiole of *Borassus* sp.	Thailand	A	[267]
*Asymmetricospora*	*A. calamicola*	On a dead stem of *Calamus caryotoides*	Australia (Queensland)	N/A	[141]
*Atrosetaphiale*	*A. flagelliformis*	On a decayed petiole of an unidentified palm	Peru	N/A	[65]
*Aunstrupia*	*A. nodipes*	On rotten and dead leaves and rotten petiole and branches of unidentified palms	China (Guangdong)	A	[268]
*Bacusphaeria*	*B. nypae*	On a petiole base of *Nypa* fruticans	Malaysia	A	[269]
*Baipadisphaeria*	*B. spathulospora*	On a trunk of *Licuala longicalycata* submerged in peat bog	Thailand	A	[270]
*Basauxia*	*B. pulchra*	On a rachis of an unidentified palm	Malaysia	N/A	[266]
*Bhadradriella*	*B. hyalina*	On fallen pods of *Roystonea regia*	India (Andhra Pradesh)	N/A	[271]
*Brachysporiopsis*	*B. chinensis*	On a decaying rachis of *Livistona chinensis*	China (Hong Kong)	N/A	[228]
*Brobdingnagia*	*B. nigeriensis*	On tissues of *Calamus* sp.	Nigeria	N/A	[212]
*Brunneiapiospora*	*B. javensis*	On a rachis of *Calamus* sp.	Indonesia (Java)	N/A	[143]
*Bulbocatenospora*	*B. complanata*	On fallen leaves of *Bactris setulosa*	Venezuela	N/A	[272]
*Cannonia*	*C. australlis*	On rotten branches of *Butia yatay*	Argentina	N/A	[204]
*Capsulospora*	*C. frondicola*	On a rachis of *Daemonorops* sp.	Brunei	N/A	[134]
*Carinispora*	*C. nypae*	On decaying intertidal fronds of *Nypa fruticans*	Brunei	N/A	[162]
*Castanedospora*	*C. pachyanthicola*	On the petiole of a dead leaf of *Sabal palmetto*	USA (Florida)	A	[273]
*Caudatispora*	*C. palmicola*	On a dead rachis of *Phytelaphas*	Ecuador	N/A	[119]
*Cenangiumella*	*C. rattanicola*	On a dead rattan sheath of *Calamus conirostris*	Brunei	N/A	[6]
*Chitinasiproducens*	*C. palmae*				
*Circinoconiopsis*	*C. amazonica*	On decaying leaves of *Oenocarpus* sp.	Brazil (Pará)	N/A	[274]
*Cocoicola*	*C. cylindrospora*	On petioles of *Cocos nucifera*	Papua New Guinea	N/A	[123]
*Corynesporasca* *	*C. caryotae*	On rotting leaves of *Caryota urens*	Sri Lanka	N/A	[275]
*Curvatispora*	*C. singaporensis*	On a fallen decaying frond of *Livistona spinosa*	Singapore	N/A	[153]
*Cyanopulvis*	*C. australiensis*	On a dead rattan of *Calamus australis*	Australia (Queensland)	N/A	[6]
*Cylindrotorula*	*C. indica*	On a decaying spathe of *Cocos nucifera*	India (Maharashtra)	A	[276]
*Diabolocovidia*	*D. claustri*	On leaves of *Serenoa repens*	USA (Florida)	A	[277]
*Dictyopalmispora*	*D. palmae*	On decaying leaves of *Licuala longicalycata*	Thailand	A	[278]
*Discopycnothyrium*	*D. palmae*	On the branches of an unidentified palm	Thailand	A	[279]
*Durispora*	*D. elaeidicola*	On dead rachides of *Elaeis guineensis*	Malaysia	N/A	[118]
*Dwibahubeeja*	*D. indica*	On leaves of *C. tenuis*	India (Uttar Pradesh)	N/A	[280]
*Endosporoideus*	*E. pedicellatus* (as *E. pedicellata*)	On a dead petiole of *Phoenix hanceana*	China (Hong Kong)	N/A	[235]
*Fasciatispora*	*F. nypae*	On a rotten frond of intertidal *Nypa fruticans*	Brunei	A	[161]
*Fissuroma*	*F. maculans*	On dead leaves of *Arenga westerhoutii*	Thailand	A	[281]
*Flammispora*	*F. bioteca*	On dead leaves of *Licuala longicalycata* submerged in peat swamp	Thailand	A	[282]
*Fluviatispora*	*F. tunicata*	On submerged rachides of *Livistona* sp.	Papua New Guinea	N/A	[174]
*Frondicola*	*F. tunitricuspis*	On decaying fronds of *Nypa fruticans*	Brunei	N/A	[162]
*Frondisphaeria*	*F. palmicola*	On a rachis of *Eugeissona minor*	Brunei	N/A	[170]
*Frondispora*	*F. bicalcarata*	On dead petioles of *Chamaerops humilis*	Italy	N/A	[111]
*Gossypinidium*	*G. sporodochiale*	On a dead rachis of *Praestoea montana*	Puerto Rico	A	[283]
*Guestia*	*G. gonetropospora*	On a dead rachis of *Mauritia flexuosa*	Ecuador	N/A	[150]
*Haploanthostomella*	*H. elaeidis*	On dead leaves and rachis of *Elaeis guineensis*	Thailand	A	[284]
*Haplohelminthosporium*	*H. calami*	On living leaves and petioles of *Calamus* sp.	Thailand	A	[285]
*Helensiella* (as *Digitella*)	*H. rigidophora* (as *D. rigidophora*)	On a rachis of an unidentified palm	Mexico (Veracruz)	N/A	[286,287]
*Helminthosporiella*	*H. stilbacea*	On a dead petiole of *Cocos nucifera*	Thailand	A	[285,288]
*Hemisynnema ^#^*	*H. malayasianum*	On a rachis of an unidentified palm	Malaysia	N/A	[289]
*Hyalobelemnospora*	*H. amazonica*	On a rotten petiole of an unidentified palm	Peru	N/A	[64]
*Kalamarospora*	*K. multiflagellata*	On rachides of dead leaves of *Sabal palmetto*	USA (Florida)	N/A	[290]
*Letendraeopsis*	*L. palmarum*	On leaves of *Euterpe oleracea*	Brazil (Pará)	N/A	[291]
*Lockerbia*	*L. palmicola*	On dead rachides of an unidentified palm	Australia (Queensland)	N/A	[114]
*Longicorpus*	*L. striatisporus* (as *L. striataspora*)	On a decayed rachis of *Nypa fruticans*	Thailand	A	[9]
*Mackenziella* (as *Mackenziea*)	*M. livistonae*	On decaying rachides of *Oraniopsis appendiculata*	Australia (Queensland)	N/A	[15]
*Maculatifrondes* (as *Maculatifrondis*)	*M. aequatoriensis*	On leaves of an unidentified palm in rainforest	Ecuador	N/A	[208]
*Maculatipalma*	*M. frondicola*	On a leaf of *Linospadix microcarya*	Australia (Queensland)	N/A	[197]
*Malthomyces*	*M. calamigena* (as *M. calamigenus*)	On tissues of *Calamus rudentum*	Sri Lanka	N/A	[212]
*Manokwaria*	*M. notabilis*	On dead rachides of an unidentified palm in freshwater swamp	Indonesia	N/A	[109]
*Monosporoschisma*	*M. elegans*	On a dead material of an unidentifed palm	Chian (Hainan)	A	[268]
*Neoastrosphaeriella*	*N. krabiensis*	On a petiole of *Metroxylon sagu*	Thailand	A	[281]
*Neobarrmaelia*	*N. hyphaenes*	On leaves of *Hyphaene* sp.	South Africa	A	[292]
*Neolinocarpon*	*N. globosicarpum*	On decaying intertidal fronds of *Nypa fruticans*	Brunei	N/A	[162]
*Neoxylaria*	*N. arengae*	On a dead petiole of *Arenga pinnata*	Thailand	A	[293]
*Nigromammilla* (as *Nigramammilla*)	*N. calami*	On a sheath of dead rattan of *Daemonorops margaritae*	China (Hong Kong)	N/A	[179]
*Nipicola*	*N. carbospora*	On immersed fronds of *Nypa fruticans*	Brunei	N/A	[163]
*Nusia*	*N. scheeleae*	On a rachis of *Scheelea insignis*	Singapore	N/A	[294]
*Nypaella*	*N. frondicola*	On intertidal fronds of *Nypa fruticans*	Brunei	N/A	[164]
*Ornatispora* ^#^	*O. palmicola*	On a dead rachis of an unidentified palm	Ecuador	N/A	[181]
*Oxodeora*	*O. petrakii*	On living fronds of *Oreodoxa regia*	Dominican Republic	N/A	[212]
*Palmaria* (as *Palmomyces*)	*P. montanea* (as *P. montaneus*)	On a leaf of *Oraniopsis appendiculata*	Australia (Queensland)	N/A	[143]
*Palmeiromyces*	*P. chamaeropicola*	On leaf spots of *Chamaerops humilis*	Portugal	A	[295]
*Palmicola*	*P. archontophoenicis*	On a fallen rachis of *Archontophoenix alexandrae*	Australia (Queensland)	N/A	[108]
*Paracapsulospora*	*P. metroxyli*	On a dead *Metroxylon sagu*	Thailand	A	[296]
*Paradactylella*	*P. peruviana*	On a rotten petiole of an unidentified palm	Peru	N/A	[64]
*Paraproliferophorum*	*P. hyphaenes*	On living leaves of *Hyphaene* sp.	South Africa	A	[297]
*Pararamichloridium*	*P. livistonae*	On leaves of *Livistona australis*	Australia (New South Wales)	A	[298]
*Parateichospora*	*P. phoenicicola*	On leaves of *Phoenix reclinata*	South Africa	A	[299]
*Phaeochoropsis*	*P. neowashingtoniae*	On leaves of *Neowashingtonia filamentosa*	USA (California)	N/A	[212]
*Phaeomonilia*	*P. pleiomorpha*	On a decaying petiole of an unidentified palm submerged in stream	Mexico (Veracruz)	N/A	[300]
*Phruensis*	*P. brunneispora*	On a dead trunk of *Licuala longicalycata*	Thailand	A	[301]
*Polybulbophiale*	*P. palmicola*	On the decaying petiole of *Licuala* sp.	Brunei	N/A	[190]
*Porodiplodia*	*P. livistonae*	On leaves of *Livistona australis*	Australia (New South Wales)	A	[302]
*Pseudopalawania*	*P. siamensis*	On a dead rachis of *Caryota* sp.	Thailand	A	[303]
*Pulmosphaeria*	*P. archontophoenicis*	On a dead petiole of *Archontophoenix alexandrae*	Australia (Queensland)	N/A	[194]
*Quasiphoma*	*Q. hyphaenes*	On leaves of *Hyphaene* sp.	South Africa	A	[292]
*Rachidicola*	*R. palmae*	On a rachis of *Calamus* sp.	China (Hong Kong)	N/A	[129]
*Rattania*	*R. setulifera*	On leaves of *Calamus thwaitesii*	India (Goa)	N/A	[304]
*Rogergoosiella*	*R. roystoneicola*	On a dead petiole of *Roystonea regia*	Cuba	N/A	[305]
*Sabalicola*	*S. sabalensioides*	On petioles of *Sabal serrulata*	USA (Florida)	N/A	[122]
*Sawantomyces*	*S. indicus* (as *S. indica*)	On a spathe of *Cocos nucifera*	India (Maharashtra)	N/A	[306]
*Setophiale*	*S. unisetulata*	On a decayed petiole of an unidentified palm	Peru	N/A	[65]
*Sorokinella*	*S. appendicospora*	On a dead petiole of *Livistona chinensis*	China (Hong Kong)	N/A	[6]
*Stratiphoromyces*	*S. brunneisporus*	On decaying petioles of *Licuala* sp.	Brunei	N/A	[189]
*Striatiguttula*	*S. nypae*	On a decayed rachis of *Nypa fruticans*	Thailand	A	[9]
*Thailandiomyces*	*T. bisetulosus*	On senescent trunks of *Licuala longicalycata*	Thailand	A	[307]
*Tirisporella*	*T. beccariana*	On decaying leaf bases of *Nypa fruticans*	Malaysia	N/A	[167]
*Tretendophragmia*	*T. palmivora*	On a rachis of *Korthalsia* sp.	Singapore	N/A	[308]
*Tretocephala*	*T. decidua*	On a leaf sheath and rachis of *Oncosperma horridum*	Singapore	N/A	[309]
*Tribulatia*	*T. appendicospora*	On a dead petiole of *Archontophoenix alexandrae*	Australia (Queensland)	N/A	[8]
*Triseptatospora*	*T. calami*	On dead petioles of *Calamus* sp.	Thailand	A	[310]
*Unisetosphaeria*	*U. penguinoides*	On a petiole of *Eleiodoxa conferta* submerged in peat swamp	Thailand	N/A	[245]
*Uwemyces*	*U. elaeidis*	On leaves of *Elaeis oleifera*	Colombia	A	[288]
*Venustocephala*	*V. aequatorialis*	On a decayed petiole of an unidentified palm	Ecuador	N/A	[65]
*Venustisporium* (as *Venustusporium*)	*V. chelyoforme* (as *V. chelysforme*)	On fallen rotten leaves of *Bactris setulosa*	Venezuela	N/A	[311]
*Veramycella*	*V. bispora*	On rachides of dead leaves of *Sabal palmetto*	USA (Florida)	N/A	[312]
*Veramyces*	*V. manuensis*	On a rotten petiole of an unidentified palm	Peru	N/A	[64]
*Waihonghopes*	*W. australiensis*	On a decaying rachis of *Oraniopsis appendiculata*	Australia (Queensland)	N/A	[15]

^1^ Availability of DNA sequence data for the type species of each genus, N/A: DNA sequence data not available; A: DNA sequence data available. * The monotypic genus *Corynesporasca* was introduced to accommodate *C. caryotae* and linked with an unnamed *Corynespora* asexual morph in culture by Sivanesan [275]. *Corynesporasca* has been treated as a synonym of *Corynespora* in several studies, e.g., ref. [313]. However, the present review follows Hyde et al. [314], who did not synonymize *Corynesporasca* under *Corynespora*. *Corynespora* was shown to be polyphyletic and *Corynespora*-like asexual morphs have been associated with many genera, e.g., ref. [315]. Therefore, the type species of both genera may be unrelated. ^#^ Genera currently synonymised under other genera. *Hemisynnema* was synonymised under *Morrisiella* by Wu and Zhuang [316]. *Ornatispora* was synonymised under *Stachybotrys* by Wang et al. [317].

There are several scattered reports of palmicolous hyphomycetes in different regions of the world. For instance, species of helicosporous fungi from various genera, such as *Drepanospora*, *Helicoma*, *Helicomyces*, *Helicosporium*, and *Xenosporium*, have been recorded in collections of palm tissues. These were reviewed by Goos [318,319,320,321,322,323,324,325] and Goos et al. [326] during their studies on anamorphic genera of helicosporous fungi. In the 1990s, along with the examples previously cited by Hyde and co-workers, several palmicolous hyphomycetes were reported in studies carried out by Subramanian in India and in Southeast Asian countries, such as Malaysia and Singapore (Figure 1). In these studies, Subramanian [259,262,266,289,294,308,309,327,328] introduced many new species and genera of dematiaceous hyphomycetes from different palm trees species, including *Tretendophragmia* [308], *Tretocephala* [309], *Ashtaangam*, *Basauxia* [266], *Acarocybellina* [259], *Agrabeeja* [262], *Nusia* [294], and *Hemisynnema* [289] (Table 1). These records further emphasised the importance of palm trees for the description of taxonomic novelties and for the study of fungal biodiversity, and the same trend would be found in the palm trees of Central American countries.

Palmicolous hyphomycetes have been widely collected from palms in Central American countries, including Cuba and Mexico (Figure 1), by Castañeda-Ruiz, Holubová-Jechová, Mena-Portales, Mercado-Sierra, and many other co-workers, following their previously mentioned investigation of hyphomycetes from Cuba in the 1980s. Although most of these studies were not conducted to explicitly evaluate palm fungi, the extent of these reports has made it possible to reveal the composition of the assemblage of palm hyphomycetes that inhabit palm tissues in the tropics. Furthermore, it has become evident, particularly in studies from Cuba, that some parts of palm trees, mainly their decaying rachides and large petioles, are exceptional substrata for the growth and development of microfungi, mainly hyphomycetes, and several new taxa have been identified [260,305,329,330,331,332,333,334,335,336,337,338,339,340,341,342,343,344].

Similar to what Hyde and co-workers discovered in their investigation in East and Southeast Asia, Australasia, and Ecuador, many of the fungi growing on palm trees in Cuba were described as new to science and found to form an autochthonous mycobiota, where host-specificity, sometimes at the host genus or species level, is often observed. The fungus–host plant relationship and the factors that affect it were expressly discussed and reviewed by Mercado-Sierra et al. [329], particularly for genera of palm trees that grow abundantly in Cuba, namely *Roystonea*, *Cocos*, and *Coccothrinax*. The Cuban royal palm *Roystonea regia* appears to be a particular case where an enormous diversity of fungi was found, including 265 species from different taxonomic groups, a number much higher than that reported for other plant species endemic to Cuba [345]. Moreover, its relevance and importance in studies of hyphomycetes were pointed out by Mercado-Sierra [73]. Many of the fungi identified on palm trees from Cuba were new reports for Cuban mycobiota and several taxa were described as new to science, including more than thirty species and six genera, viz. *Consetiella* [75], *Holubovaea* [73], *Phragmospathulella* [96], *Cheiromyceopsis* [99], *Rogergoosiella* [305], and *Acarocybiopsis* [260] (Table 1).

Several Cuban provinces and localities, such as Pinar Del Río, Camagüey, Matazanas, and Sancti Spíritus, have been extensively sampled during surveys of hyphomycetes in protected natural areas in Cuba, and on several occasions these hyphomycetes have been collected from palm trees, e.g., refs. [338,346,347,348,349,350,351]. Although the extension of these studies made it possible to uncover the assemblage of palm hyphomycetes in those locations, their objective was mainly to study ecological stations, biosphere reserves, and protected areas in order to promote the conservation of important hotspots of fungal diversity. These studies continue to be carried out today and report an exceptional diversity of palm fungi. Recently, Mena-Portales et al. [352], following Mercado-Sierra et al. [329], reviewed and analysed the relationship between fungal diversity and palms trees in Cuba by compiling information on some interesting species of hyphomycetes found in different *Arecaceae* hosts.

Similar studies have also been carried out in other Central American countries (Figure 1). Mercado-Sierra et al. [353,354] reported some palmicolous hyphomycetes from Costa Rica. Recently, a checklist of asexual fungi from Costa Rica, which compiled information obtained during 1927 to 2018 based on scientific papers, was presented by Granados-Montero et al. [355], including several reports of hyphomycetes and coelomycetes on palm trees. Very few palm fungi, including hyphomycetes, have been reported from Puerto Rico and most reports have been summarised in an annotated bibliography entitled “The Fungi of Puerto Rico and the American Virgin Islands” by Stevenson [356]. However, some new species were later introduced based on occasional palm collections, e.g., refs. [240,357,358]. Palmicolous hyphomycetes from Panama and Nicaragua have been listed in checklists of Panamanian and Nicaraguan fungi by Piepenbring [359] and Delgado-Rodríguez [360], respectively. Likewise, several palmicolous hyphomycetes were reported in Mexico during studies carried out in order to increase the knowledge about Mexican hyphomycetes [286,300,361,362,363,364,365,366,367,368,369,370,371], where two new genera, *Phaeomonilia* [300] and *Digitella* [286], and some new species were introduced (Table 1). Similar to the studies in Cuba, the investigation in Mexico, which continues today, was not pursued to study palm fungi, but to inventory and gain knowledge about conidial fungi from plant litter in tropical forests, particularly in the states of Campeche, Tabasco, and Veracruz, due to the accelerated deforestation of tropical habitats, e.g., refs. [367,371,372,373,374,375,376,377,378,379,380]. As a result, since palm trees are an important component of the flora of these forests, several palmicolous hyphomycetes have been reported.

In addition to the studies in Central American countries, South American countries have also been occasionally surveyed (Figure 1). Therefore, palmicolous hyphomycetes have been reported in collections from Argentina [381], Colombia [382], Peru [265], and Venezuela [272,311,383,384,385,386,387], where some new species and genera have been introduced (Table 1).

#### 2.1.4. Palm Fungi from Understudied Tropical Hotspots, Argentina, India, and Brazil

Some scattered studies have surveyed palmicolous fungi in Argentina. However, with the exception of Mercado-Sierra et al. [381], these were not systematic studies of descriptive taxonomy, but studies carried out to better understand the diversity of ascomycetes in woody parts of palms in Argentina, especially in areas or parks that had been proposed as natural reserves for protection and where some native palm species are an important element of the local flora, including *Butia yatay*, *Euterpe edulis*, and *Syagrus romanzoffiana* [388,389,390]. Capdet and Romero [389] summarised previous information on palm fungi and their occurrence in Argentina, reflecting on the lack of knowledge about palm fungi in the country.

Similarly, considering the available literature on palm fungi, except for a few stray collections, no comprehensive investigation on the fungal diversity that occurs on palms in India has been carried out (Figure 1). However, some remarkable reports by Subramanian and his students from Chennai, P. R. Rao, D. Rao, and V. Rao, and colleagues from Hyderabad and Bhat and colleagues from Goa, revealed a considerable diversity of palmicolous fungi, especially hyphomycetes, occurring on leaf litter. An overview of these studies is given here.

Subramanian [391,392,393,394,395,396,397], in his series of seven papers “Fungi imperfecti from Madras”, reported several new palmicolous hyphomycetes from dead palm leaves in Chennai. Moreover, in addition to his previously mentioned reports on dematiaceous hyphomycetes [259,262,266,289,294,308,309,327,328], Subramanian extensively studied hyphomycetes in tropical regions, especially India. These studies resulted in a major monograph of the Indian species [398], many of which were collected from palm material and introduced as new to science [399,400,401,402,403,404,405,406]. A number of these new palmicolous hyphomycetes were introduced in his series of six papers entitled “Hyphomycetes”, where new genera and species were described on the leaves of several different palm species, such as *Cocos nucifera*, *Phoenix canariensis*, *Rhopalostylis sapida*, and *Borassus flabellifer* from Tamil Nadu [407,408,409,410,411,412].

Rao and Rao [413,414,415,416,417,418,419,420], Chaudhury and Rao [421], and Rao and Chaudhury [422], also reported several palmicolous fungi, including new taxa, from different palm species, such as *B. flabellifer*, *Caryota urens*, *C. nucifera*, and *Livistona chinensis*, mainly from Hyderabad. Moreover, new species of palmicolous fungi were reported by Rao [423,424,425] from Maharashtra. Later, Varghese and Rao [426,427] recorded several palm fungi during their mycological survey of the forests of Kerala, near where Pande and Rao [428] collected the new species *Rosellinia lakshadweepensis* on the pericarp of *C. nucifera* from the island of Kavaratti (Lakshadweep).

Bhat and co-workers isolated some new taxa of palmicolous litter-inhabiting hyphomycetes in palm collections from India in their studies on fungi from the forests of the Western Ghats hills in Goa, the rainforests of the Andaman-Nicobar Islands and, to a lesser extent, the humid mountains forests of Northeastern Himalayas, e.g., refs. [429,430,431,432]. In addition to these taxonomic studies, ecological studies were also carried out on litter colonisers and endophytes in plant species from the forests of the Western Ghats in Goa, which included palm trees species, such as *Calamus thwaitesii*. *C. urens*, and *Elaeis guineensis* [433,434].

A vast area of the peninsular India still remains underexplored for fungal biodiversity [435]. However, the fungi of the forests of the Western Ghats in Goa and the rainforests of the Andaman-Nicobar Islands, two of the biological hotspots of India, have been explored to some extent, e.g., refs. [436,437,438,439,440,441]. In turn, many palm fungi have been documented, including several new taxa (Figure 1, Table 1). Most of these studies were reviewed by Bhat [437], who considered the forests of the Western Ghats as “an abode of novel and interesting microfungi” and presented a list of new hyphomycetes discovered in them, where several palmicolous fungi are cited. Further studies on the diversity of microfungi from these forests in Goa and in some parts of Karnataka, Kerala, Tamil Nadu, and Maharashtra were carried out by Pratibha et al. [442,443], which resulted in the documentation of a few more palmicolous hyphomycetes. Still, today studies on the fungi of the forests of the Western Ghats hills and the rainforests of the Andaman-Nicobar Islands continue to be carried out and to report an exceptional diversity of palm fungi. For instance, Dubey and Moonnambeth [306,444,445,446,447,448], Dubey [449], and Dubey and Neelima [450] documented several dematiaceous palmicolous hyphomycetes, including new taxa, during an investigation of fungi from the forests of the Western Ghats of Maharashtra (Table 1). More recently, Niranjan and Sarma [451] compiled a checklist of fungi reported from the Andaman-Nicobar Islands, where many palmicolous fungi have been documented. Several other palmicolous fungi, including new species, have been reported from the rainforests of these islands by Ram and Sinha [452] and Niranjan and Sarma [453,454,455,456,457].

Although information on Indian palm fungi is scarce and difficult to review and compile properly, fungi collected from living palm leaves, diseased palm foliage, and palm litter by various researchers have been included in periodically published lists and compilations of Indian fungi, for example “List of Indian fungi 1952–1956” [404], “List of Indian fungi 1956–1960” [458], “Fungi of India 1989–2001” [459], “Ascomycetes of Peninsular India” [460], and, more recently, “Bilgrami’s Fungi of India List and References (1988–2020)” [461]. One of these compilations was dedicated exclusively to palm fungi under the title “Fungal records on palms from India” by Pande et al. [462], who made a list of fungi recorded on palm trees up to 1999 and listed 355 species distributed in 188 genera described from 29 species of palm trees from different Indian states.

Brazil is a country rich in palm species, some of which have great socio-economic value [463]. Even so, few taxonomic studies have been carried out on Brazilian palm fungi (Figure 1) and most of them have focused on palm hyphomycetes. However, the few studies available have also revealed the trend towards the presence of a rich fungal diversity, from which many new palmicolous fungi have been introduced. A summary of some examples of these studies is given herein. In 1978, Hennen and Ono [464] identified the first rust fungus on a palm tree, the new genus and species *Cerradoa palmaea* on *Attalea ceraensis* from Brasília. Several palm fungi were gathered from collections of *Astrocaryum* from Amazonas and published by Farr [465,466,467] in his series of papers “Amazonian foliicolous fungi”. Later, Rodrigues and her colleagues recorded some new palmicolous taxa when studying the endophytic fungi that inhabit the tissues of the Amazonian palm *Euterpe oleracea* [291,468,469,470,471,472,473] (Table 1). Rodrigues [468,472] published the first study on the fungal endophytes inhabiting the foliage of *E. oleracea* from Combu Island, growing in the Brazilian Amazon estuary and in Amazonian floodplains, and listed 57 species of palm ascomycetes, including several hyphomycetes. In addition, several fungi recorded on arecaceous hosts from Brazil have been compiled by Silva and Minter [474] and Mendes et al. [475,476].

Many other sparse reports of palm fungi from Brazilian regions have been made. However, similar to the studies on palm fungi from Argentina, most of the studies on Brazilian palm fungi were not carried out as systematic studies of descriptive taxonomy. Instead, these studies aimed to better understand the diversity of conidial fungi, especially hyphomycetes, on woody palms of the Amazon rainforest and other biomes, where biodiversity research and conservation programs were being conducted. An overview of these studies is presented here.

The investigation on conidial fungi associated with the decomposition of palm leaves in the Amazon rainforest has been restricted to three areas, namely the National Forest of Caxiuanã [274,387,477,478,479,480,481] and the Combu Island [482,483] in Pará, and the National Forest of Amapá in Amapá [264,387,479,481]. Several *Arecaceae* hosts have been sampled, including members of *Astrocaryum*, *Attalea*, *Bactris*, *Euterpe*, *Geonoma*, *Maximiliana*, *Oenocarpus*, *Socratea*, and *Syagrus*. Most of these studies were motivated both by the accelerated deforestation of the Amazon rainforest and by the lack of research into the diversity of palm fungi in the tropical regions of South America. In addition to the importance of these studies for expanding the knowledge about the composition and distribution of palm fungi in the Amazon rainforest, several new records for these Brazilian regions, as well as new genera and species, have been reported, e.g., refs. [264,274,387,479] (Table 1).

Surveys of microfungi on palm trees have also been carried out in Bahia, Brasília, and Pernambuco, particularly in biomes in areas of Northeast Brazil where palm trees are a major component [484,485,486,487,488,489,490,491,492,493,494,495,496,497,498,499,500,501,502,503,504,505,506,507,508,509,510,511,512,513,514,515,516]. These studies aimed to understand the fungal composition of important biomes of Northeast Brazil, such as the Atlantic rainforest, e.g., refs. [496,499,500], the Caatinga, e.g., refs. [497,501,502,503,504,507,508,509,512], and the Cerrado, e.g., refs. [485,486,489], as well as to uncover the mycota that inhabit the tissues of important palms that can be found there, including *Acrocomia intumescens*, *Attalea funifera*, *Bactris acanthocarpa*, *Cocos nucifera*, *Elaeis guineensis*, *Euterpe edulis*, *E. oleracea*, *Mauritia flexuosa*, *Polyandrococos caudescens*, *Syagrus botryophora*, and *S. coronata*. In turn, several new palmicolous taxa have been reported, including members of the typical palmicolous mycota found by Hyde and co-workers on their extensive investigation on East and Southeast Asian and Australasian countries. For instance, Vitória et al. [491,495] introduced the new species *Arecomyces attaleae* and *Neolinocarpon attaleae* on dead rachides of *A. funifera* from Bahia.

#### 2.1.5. Palm Fungi and Reflections on the Recent Input from Molecular Era

This comprehensive overview of the literature shows that palms support a vast array of fungi, especially ascomycetes. In the well-studied tropical regions of East and Southeast Asia, Australasia, and, to a lesser extent, Central America (Figure 1), where these fungi have received considerable attention, a remarkable diversity of fungi has been revealed with the description of numerous new taxa, e.g., refs. [6,8,104,329,352]. Much of this diversity can be attributed to the tropical and subtropical habitats surveyed, where the diversity of fungi is known to be higher [10]. However, the few studies carried out on palms that thrive in temperate regions have also revealed a considerably rich fungal diversity, of which some taxa have been described as new to science. For instance, several palmicolous fungi have been recorded in New Zealand (Figure 1), particularly from collections of *Rhopalostylis* spp. [54,517,518,519,520,521]. McKenzie et al. [54] noted that 147 named fungal species and 50 fungal records identified only to genus have been recorded on *Rhopalostylis*, mainly on *R. sapida* from New Zealand. Thus, the great diversity of palmicolous fungi recovered can be also attributed to the wide variety of palm hosts and habitats studied, including many different palm species and tissues in terrestrial, freshwater, and marine or mangrove ecosystems. While the first studies by Hyde and co-workers covered the diversity of fungi on mangrove palms, e.g., ref. [168], most subsequent studies were dedicated to surveying saprobic fungi that develop on palm substrata from tropical rainforests, e.g., ref. [150]. In addition, few studies were conducted to survey palm phytopathogens, e.g., ref. [212].

Up to 2003, the intensive research carried out by Hyde and co-workers has reported more than 320 new species and more than 45 new genera of palmicolous fungi. However, in all these earlier studies, all the taxa reported were introduced, described, and arranged in different taxonomic ranks within the *Ascomycota* based solely on their morphology. This is currently known as a subjective approach and many taxa have been assigned to *Ascomycota* genera *incertae sedis*. Given that palm trees are important hosts that harbour potential novel taxa, it is critical that these palmicolous fungi are recollected, epitypified where needed, and isolated so that molecular data can be obtained and used to establish their natural phylogenetic placements [522]. For example, regarding the initial examples of some of the most common fungal genera found on palms, such as *Anthostomella*, *Astrosphaeriella*, *Linocarpon*, and *Oxydothis*, several species have been recorded on palms, e.g., refs. [111,134,138], but only a small percentage of these have associated sequence data available. As a result, their position in a natural taxonomic framework is poorly supported and, consequently, their evolutionary relationships in higher taxonomic ranks are poorly understood.

Some of the first studies on palmicolous fungi that combined molecular data with morphological data were those by Pinruan et al. [270,282,301,307,523] and Pinnoi et al. [524,525] in Thailand, who reported several new taxa of saprobic fungi on *Calamus* species and on the peat swamp palms *Licuala longecalycata* and *Eleiodoxa conferta*. These studies were a continuation of previous solely morphological studies that had already yielded a remarkably rich fungal diversity from palms in the Sirindhom Peat Swamp Forest at Narathiwat, Southern Thailand [236,241,242,243,244,245,246,247]. Four new genera have been introduced (Table 1) and their phylogenetic relationships among extant taxa have begun to be unveiled. An interesting example worth mentioning is the description of the new species *Astrocystis eleiodoxae* on petioles of *E. conferta* submerged in a peat swamp from Thailand by Pinnoi et al. [525]. Much of the early studies by Hyde and co-workers focused on the treatment of several genera that occur on palms and their taxonomic relationships and placement based mainly on morphological characters related to asci, ascospores, and associated features. The taxonomic placement of the genus *Astrocystis*, as well as several other xylariaceous-related genera commonly recorded on palms, has been analysed, although it has often remained obscure due to the assessment of its morphology alone, e.g., ref. [150]. The description of *A. eleiodoxae* by morphological and phylogenetic means, as well as other new xylariaceous taxa from palms, such as *Rosellinia capetribulensis* on decaying rachides of *Calamus* sp. from Northern Queensland, Australia by Bahl et al. [526], underlines the importance of introducing molecular data into the study of palm fungi as a highly diverse fungal group. Although the assignment of these taxa to higher taxonomic ranks remained ambiguous and undetermined, the relationship with other xylariaceous genera started to become clearer than when these studies were conducted solely on the basis of morphology.

The introduction of DNA sequence data to study fungal biodiversity in the early 1990s has served as a stimulus for the description of new taxa from palms. Hence, several of the old collections have been accessed and their taxonomic placement clarified. Over the last 15 years, Hyde and co-workers have been revisiting their studies on palms, recollecting and epitypifying some of the taxa and clarifying their phylogenetic position among extant and new taxa [522]. This, in turn, has refined the identity of the most common mycota that inhabits palm tissues by providing information on the natural grouping of palmicolous genera based on sequence analyses (Table 2). The number of reports has been increasing rapidly, especially in collections from Northern Thailand (Figure 1), revealing several new palmicolous taxa that have complexified the taxonomic structure of palm fungi (Table 1 and Table 2).

Several new species of the common palm mycota were introduced, along with new genera, including members of the *Sordariomycetes* and *Dothideomycetes* frequently found on palms. This ultimately led to the establishment of new families to accommodate and clarify their phylogenetic relationships. Indeed, new taxa of some of the most common fungal genera found on palms, including *Oxydothis* [527,528,529], *Linocarpon*, *Neolinocarpon* [530], *Astrosphaeriella sensu lato* [267,281,531,532,533], and *Roussoella* [534], are continuously reported on palm tissues and the new families *Oxydothidaceae* [528], *Linocarpaceae* [530], *Astrosphaeriellaceae* [267], and *Roussoellaceae* [534], respectively, have been introduced to accommodate them (Table 2). A historical account of molecular studies carried out on palm fungi is presented herein. In addition, some case studies of common palm taxa are highlighted to reflect on the impact of molecular data on the taxonomy and biodiversity of this group of fungi.

##### *Astrosphaeriella*-like Taxa: A Polyphyletic Nature Hiding Cryptic Genera

*Astrosphaeriella* is an interesting case study of how phylogenetics and its inputs had implications in the taxonomic structure of palm fungi. Although there have been many morphological-based studies of *Astrosphaeriella*, including those major morphological studies by Hyde and Fröhlich [144] and Hyde et al. [148], based particularly on species of *Astrosphaeriella* occurring on palms in tropical regions, no thorough molecular investigation of the genus has been carried out. The taxonomy of *Astrosphaeriella* and its natural placement, especially at family level, stayed unresolved until recently. The frequent collection of *Astrosphaeriella*-like species on palms and other monocotyledonous trees has made it possible to move towards their natural classification. Thus, in addition to understanding the polyphyletic nature of *Astrosphaeriella sensu lato*, Liu et al. [281] erected two new genera of *Astrosphaeriella*-like species in *Aigialaceae*, viz. *Fissuroma* and *Neoastrosphaeriella* (Table 1). Later, Phookamsak et al. [267] recognised that *Astrosphaeriella*-like species can be distinguished into three families and established *Astrosphaeriellaceae* for typical *Astrosphaeriella* species (*sensu stricto*), *Pseudoastrosphaeriellaceae* to accommodate the new genus *Pseudoastrosphaeriella*, and the new genus *Astrosphaeriellopsis* for a distinct *Astrosphaeriella*-like lineage basal to *Aigialaceae* (Table 1). *Astrosphaeriellopsis* was later accommodated in *Astrosphaeriellaceae* by Wanasinghe et al. [532] following a multigene phylogeny and respective taxonomic circumscription of *Astrosphaeriella* species and allied genera with the introduction of several novel palmicolous taxa in the genera *Astrosphaeriellopsis*, *Fissuroma*, *Neoastrosphaeriella*, and *Pithomyces* isolated from *Calamus*, *Caryota*, and *Licuala* species in Northern Thailand and Southwest China. Further additions to *Fissuroma* and *Neoastrosphaeriella* were made by Konta et al. [533] and Zhang et al. [531] from palms in mangrove and terrestrial habitats in Thailand. Another genus, *Xenoastrosphaeriella* (Table 1), was introduced in *Astrosphaeriellaceae* to accommodate saprobic fungi on bamboo and palms that previously represented a basal lineage in *Astrosphaeriellaceae* [535]. More recently, the new genus *Triseptatospora* was introduced in *Astrophaeriellaceae* to accommodate *T. calami* found on dead petioles of *Calamus* sp. in Thailand [310] (Table 1).

Several new *Dothideomycetes*, along with *Astrosphaeriella sensu lato* and *Roussoellaceae*, are also being recorded from palm tree collections. Mapook et al. [536] introduced the new family *Palawaniaceae* to accommodate the *Palawania* species, which are saprobes common on palms, occurring on dried fronds and spines. Later, Jayasiri et al. [535] introduced two new species of palmicolous *Dothideomycetes* in *Delitschia* and *Vaginatispora* on fallen fruit pericarp of *Nypa fruticans* from Thailand. The new genus *Pseudopalawania* was introduced in *Muyocopronaceae* by Mapook et al. [303] to accommodate *P. siamensis* on a dead rachis of *Caryota* sp. from Thailand (Table 1). Recently, Yu et al. [537] made new additions to *Occultibambusaceae* based on collections of decaying petioles of *Trachycarpus fortunei* from China. Several aquatic *Dothideomycetes*, as well as *Sordariomycetes*, have also recently been described from palm trees and will be discussed later.

##### *Xylarialean* and Related *Sordariomycetes*: The Enigmatic *Anthostomella* and Allied Genera

Regarding *Sordariomycetes*, along with *Oxydothidaceae* and *Linocarpaceae*, many other new ascomycetes are being introduced and their phylogeny resolved based on collections from palms. However, several palmicolous genera are poorly represented with sequence data. In addition, several sequences are of poor quality, which can compromise the resolution of the phylogenetic placement of taxa, especially at higher taxonomic levels, such as family and class [538]. This has often been observed in studies on palm fungi. For example, while introducing *Linocarpaceae* to accommodate *Linocarpon* and *Neolinocarpon*, Konta et al. [530] also introduced the new family *Leptosporellaceae* in *Chaetosphaeriales* to accommodate *Leptosporella*, including two new species collected from palms, and provided a comparative morphological list of species in *Leptosporella*, *Linocarpon*, and *Neolinocarpon*. However, as the authors stated, fresh collections of several of the known taxa with associated molecular data are needed to establish and strengthen their natural phylogenetic placements, since all the diagnosed clades are sparsely populated. Similarly, a checklist of *Diatrypaceae* that occur on palms was presented by Konta et al. [263], with the introduction of many new species and a new genus, *Allodiatrype* (Table 1). However, the generic taxonomic resolution of several lineages remained unclear, probably due to a lack of sequence data or previous misidentifications, so their phylogenetic placement will only be clear with fresh collections and adequate sequence data [538].

The need to recollect and isolate some of the old collections of palm fungi was also reinforced by Daranagama et al. [539], who re-examined the type specimens of some *Sordariomycetes* genera to determine their family placement according to modern taxonomic concepts. As previously mentioned, the taxonomic placement of several xylariaceous genera, including those commonly recorded on palms, is often obscure due to the assessment of their morphology alone and the lack of sequence data to properly place them in a natural taxonomic framework. Moreover, novel taxa are often introduced when new collections and their respective molecular data are obtained. For instance, Konta et al. [293] introduced the new genus *Neoxylaria* (Table 1) to accommodate the new species *N. arengae*, as well as the morphological species *Xylaria juruensis* (as *N. juruensis*) from palm material in Brazil and *X. queenslandica* (as *N. queenslandica*) from *Archontophoenix alexandrae* in Australia, using both their fresh collection of *N. arengae* and morphology data, as there is no molecular data for these old collections. More recently, two new families were introduced for genera that frequently occur on palms, along with the introduction of new species. *Appendicosporaceae* was introduced to accommodate *Appendicospora*, with the analysis of a new fresh collection designed as a reference specimen for *A. hongkongensis* on dead fronds of *Livistona chinensis* from China [540]. *Fasciatisporaceae* was introduced to accommodate *Fasciatispora*, with the introduction of the new species *F. cocoes* on decaying rachides of *Cocos nucifera* from Thailand [541].

Several species of *Fasciatispora* have been introduced from palm trees since the first studies by Hyde et al. in the 1990s, and a synopsis of *Fasciatispora* species, along with *Anthostomella*, from decaying palm fronds collected in Indonesia and Thailand was presented by Hidayat et al. [542]. However, only a few of them have molecular data available for inclusion in modern taxonomic concepts and most of the new species from the 1990s remain described based only on their morphology. Recently, the type species of the genus, *F. nypae*, was re-collected on a frond of *Nypa fruticans* from Thailand and a reference specimen was designated, which made it possible to begin to resolve the phylogeny of *Fasciatispora* as a basal clade in *Xylariaceae* [543], which was later introduced as the family *Fasciatisporaceae* [541].

Likewise, several *Anthostomella* species have been introduced from palm trees and, since its first synopsis by Hyde [133], who accepted twenty-seven species (of which nine were new) occurring on palms, several other palmicolous *Anthostomella* species have been described, e.g., refs. [143,209,218,219,220,221]. However, the phylogeny of this species-rich genus is still ambiguous. Although its polyphyletic nature has already been recognised, a significant phylogenetic and morphological re-assessment of *Anthostomella* is still needed, including fresh collections and associated molecular data [539,544,545]. None of the *Anthostomella* species described from palms have been re-evaluated to gain knowledge about their phylogenetics, so it can be assumed that several new taxa have yet to be described under morphological *Anthostomella*-like species. In fact, several *Anthostomella*-like species have been analysed and placed in existing and new genera to properly accommodate them, e.g., refs. [539,541,544,545]. For instance, recently, Konta et al. [284] introduced the new genus *Haploanthostomella* from palms based on *H. elaeidis* on dead leaves and rachides of *Elaeis guineensis* from Thailand (Table 1) and provided a key to genera with *Anthostomella*-like characteristics. Konta et al. [284] also provided a family replacement of *Endocalyx* to *Cainiaceae*. *Endocalyx* is a genus of coelomycetes almost exclusively reported on palm hosts [546,547]. Although Delgado-Rodríguez et al. [546] made a recent phylogenetic assessment of *Endocalyx*, some species still lack molecular data, as is often the case with xylariaceous genera.

##### Palmicolous “Anamorphs”: A Plethora of *Botryosphaeriaceae* and Other *Dothideomycetes*

Following the trends of the late 1990s, along with the description of the *Ascomycota* coverage through the presence of its sexual morphs on the host, reports on palmicolous “anamorphs” began to become more frequent, e.g., refs. [241,243,244,245,246]. In recent years, several species of *Botryosphaeriaceae* are being introduced as new to science based on palm collections from different regions of the world, particularly species of *Neodeightonia* [505,547,548,549,550,551,552,553]. Moreover, some new pestalotioid-like species have recently been described on palms, including species of *Neopestalotiopsis*, *Pestalotiopsis*, *Pseudopestalotiopsis* [296,310,554,555,556,557,558,559], *Seridium* [560], and *Morinia* [561].

Dematiaceous hyphomycetes, as demonstrated by studies on palmicolous hyphomycetes from Central American countries and India, are also frequently reported on palm tissues and their phylogenetics is continuously revealing new taxa. Li et al. [562] introduced the new family *Zygosporiaceae* to accommodate *Zygosporium*, a widespread genus usually associated with monocotyledonous, including palms. Delgado-Rodríguez, in his series of papers on “South Florida microfungi”, in which many new species of palm hyphomycetes were described, e.g., refs. [290,312,563,564,565,566], introduced a new species of *Taeniolella* on the petiole of a dead leaf of *Sabal palmetto*, expanding the concept of the strong polyphyly of the genus among different classes [567]. Later, Delgado-Rodríguez and co-workers introduced the new genus *Castanedospora* to accommodate *Sporidesmium pachyanthicola* based on an epitype specimen collected on the petiole of a dead leaf of *S. palmetto*, redefining its placement at family level in the *Extremaceae* [273] (Table 1). Moreover, the phylogenetic placement of *Ernakulamia cochinensis*, one of the saprobic hyphomycetes taxa commonly found associated with palm hosts, as a member of *Tetraplosphaeriaceae* in *Pleosporales*, was investigated based on a representative specimen collected on *Astrocaryum standleyanum* in Panama [568]. Species of *Hermatomyces* are being described on palms from different regions of the world, including Panama [569], Thailand [570], and Texas (USA) [571], in addition to other exclusively morphological studies that have reported *Hermatomyces* species from palm trees collections in India [572] and Sierra Leone [573] (Figure 1, Table 2). More recently, Konta et al. [285] described *Helminthosporium*-like taxa from palms in Thailand and introduced two new genera in *Massarinaceae*, viz. *Haplohelminthosporium* and *Helminthosporiella* (Table 1). Konta et al. [285] also provided a checklist for *Helminthosporium* reported worldwide and most *Helminthosporium* species described from palms were based on morphology alone and only one species, *H. livistonae* on leaves of *Livistona australis* from Australia [302], was based on both morphology and sequence data. Chen et al. [574] also introduced the new species *H. chinense* on a decaying branch of an unidentified palm tree from China.

Many other occasional reports of palmicolous “anamorphs”, including new genera and species, are continuously published, revealing the plethora of genera that make up the hyphomycetous and coelomycetous assemblage that inhabit palm tissues, e.g., refs. [268,279,310,575,576,577,578] (Table 2). For example, Hongsanan et al. [279] described the new genus and species *Discopycnothyrium palmae* on the branches of an unidentified palm from Thailand (Table 1). A number of new hypocrealean members have been recently introduced based on palm collections from French Guiana, including species of *Chaetopsina* [579], *Clonostachys* [580,581], *Hydropisphaera* [582], *Ijuhya* [583,584], *Lasionectria* [585], and *Volutella* [586]. Several novel anamorphic chaetosphaereaceous fungi, including new genera, were reported from palm collections from China [268,575,577,578] and Thailand [310] (Table 1).

Crane and Miller [587] introduced some new species in *Torula*-like genera from palms, such as *Bahusaganda* and *Bahusandhika*, but no molecular data was obtained to establish their natural placements. Although the importance of acquiring sequence data to identify fungal taxa is now widely recognised and advisable [588,589,590], there are still several publications that have been and continue to be published without the support of molecular methods, introducing new morphological palmicolous taxa. For example, Wulandari et al. [591], while synopsising the species of *Phyllosticta* (as *Guignardia*) described from palm trees, introduced two new species from Northern Thailand based on morphology. Lechat and Fournier [592] introduced the new species *Lasionectria marigotensis* on a decaying leaf of *Cocos nucifera* from Guadeloupe (French West Indies) by comparing its morphology with a previous collection of *Lasionectria*, *L. calamicola*, from palms in Australia and Brunei Darussalam by Fröhlich and Hyde [6]. Later, the new species *Dictyocheirospora indica* (as *Dictyosporium indicum*) was collected on a petiole of *Phoenix rupicola* from India [593] and the new species *Endophragmiella licualae* was collected on dead branches of *Licuala fordiana* from China [594]. More recently, several palmicolous fungi, including new species, have been reported from the rainforests of the Andaman-Nicobar Islands (India) by Niranjan and Sarma [453,454,455,457] based on morphology alone, including important palmicolous genera whose taxonomic resolution highly relies on DNA sequence data, such as members of *Aigialaceae*, viz. *Fissuroma* and *Neoastrosphaeriella*, and *Astrosphaeriellaceae*, viz. *Astrosphaeriella*. Likewise, as already mentioned, many new species and genera of palmicolous hyphomycetes have been described on the basis of their morphology in Central and South American countries, including Mexico, Argentina, and Brazil.

The consistent new discoveries of fungi from palm hosts suggest their considerable potential for the identification of novel fungal taxa. Thus, using morphology alone to introduce new palmicolous taxa is strongly discouraged here. Although many fungal species have unique and distinctive characters that make them easily identifiable, especially in genera of hyphomycetes, the existence of cryptic species and species complexes has highlighted the importance of molecular methods in fungal identification [590,595]. In fact, the morphological species concept is thought to underestimate the number of species, since morphological characters can be very plastic (phenotypic plasticity) and often defines groups of cryptic species [590].

##### From Aquatic to Phytopathogenic Fungi: The Broad Taxonomic Spectrum of Palm Fungi

Freshwater and marine or mangrove ecosystems have also revealed a remarkable diversity of new palmicolous taxa with the incorporation of phylogenetics into the previous regular morphological studies [9,261,269,278,596,597,598,599,600,601,602,603,604]. The mangrove palm *Nypa fruticans* has proven to be a very distinctive habitat for fungal research since 1988, when Hyde began his studies to understand the assemblage of fungi that colonise palm tissues [154,162]. In fact, many new palmicolous fungi are continuously being described from *N. fruticans*. For instance, Zhang et al. [261] introduced the new genus *Acuminatispora* isolated from decayed petioles and rachides of palms in mangrove habitats in Thailand, including *N. fruticans* and *Phoenix paludosa*. More recently, Jones et al. [604] described the new species *Salsuginea phoenicis* on a decaying petiole of *P. paludosa* from the intertidal zone in Thailand. Thus, mangrove and peat swamp palms have been the substratum for the collection of various new aquatic fungi (freshwater and/or marine ascomycetes), whose phylogenetic resolution has often led to the description of new families and genera. In fact, four families have been recently introduced to accommodate new and extant taxa of aquatic fungi with affiliations to palm habitats. *Falciformispora* and *Trematosphaeria*, with representatives found on palms, were assigned to the new family *Trematosphaeriaceae* [596,598]. Suetrong et al. [599] introduced the new family *Tirisporellaceae* in *Diaporthales* to accommodate the genera *Tirisporella* and *Thailandiomyces* collected from palms in freshwater streams or peat swamps, and Abdel-Wahab et al. [269] added the new genus *Bacusphaeria* isolated from the petiole base of *N. fruticans* from Malaysia. The new family *Dictyosporiaceae* was established to accommodate saprobic fungi that occur on decaying wood and plant debris in terrestrial and freshwater habitats, including *Dictyosporium*, often found on palm trees, and the new genus *Dictyopalmispora* described from decaying leaves of *Licuala longecalycata* in a peat swamp forest in Thailand [278,602,603] (Table 1). Later, Zhang et al. [9] introduced another new pleosporalean family, *Striatiguttulaceae*, to accommodate two monophyletic lineages described from decayed rachides of *N. fruticans* and *P. paludosa*, the new genera *Longicorpus* and *Striatiguttula*.

In the last decade, microfungi that occur as saprobes, pathogens, and endophytes on palm hosts are commonly introduced and reanalysed in mycological series, such as Fungal Diversity notes, e.g., refs. [296,605,606,607,608], Fungal Planet description sheets, e.g., refs. [609,610,611,612,613,614,615,616], and Mycosphere notes, e.g., refs. [617,618,619,620], as well many other similar publications, e.g., refs. [621,622,623,624,625,626,627,628,629]. A remarkable taxonomic novelty published recently based on a palm collection was the new order *Pararamichloridiales* introduced to accommodate the new family *Pararamichloridiaceae* based on the new genus and species *Pararamichloridium livistonae* on leaves of *Livistona australis* from New South Wales, Australia [298]. Therefore, these occasional publications continue to expand both the broad taxonomic spectrum of palm fungi and the importance of studying them as phytopathogens and other ecological groups. In fact, in recent years, a series of new palmicolous phytopathogens have been introduced and have highlighted the lack of knowledge about the ecology of palmicolous fungi. For instance, the new species *Cercospora arecacearum* has been found associated with necrotic leaflets of areca palms in Thailand by To-anun et al. [630]. Kinge and Mih [631] described the new basidiomycete *Ganoderma ryvardenii* (as *G. ryvardense*) associated with basal stem rot disease of oil palm in Cameroon, one of the main production constraints faced by agro-industries and smallholders’ farmers in oil palm production. Moreover, Mbenoun et al. [632] described the new species *Thielaviopsis cerberus* (as *Ceratocystis cerberus*) on the stump of a felled *Elaeis guineensis* tree from Cameroon, while studying species boundaries in the *C. paradoxa* complex, a serious constraint to the cultivation of monocotyledonous crops, including the trunk rot affecting almost all palm species. Considerations on the importance of these diseases as one of the main causes of losses in oil and ornamental palms have been put forward by Aiello et al. [633], while introducing the new phytopathogen species *Ilyonectria palmarum*. More recently, the new genus and species *Palmeiromyces chamaeropicola* was collected from diseased foliage of *Chamaerops humilis* from Portugal [295], revealing a new insight into *Teratosphaeriaceae* leaf diseases, which are caused by important phytopathogens of various plant hosts.

Research into palm fungi continuously reveals the importance of the *Arecaceae* family as host plants to search for novel taxa. In addition, recent studies have been important in showing the imperative need to apply molecular data to resolve the taxonomic structure of palm fungi as an ecologically diverse and important assemblage.

### 2.2. History of Biodiversity and Ecological Studies on Palm Fungi

Palm trees have proven to be a diverse habitat, exhibiting intense fungal colonisation. Most studies on microfungi that colonise palms are taxonomic, insomuch as they have primarily focused on cataloguing fungi and describing new taxa collected on palm substrata from various regions of the world, especially in the tropics (Figure 1). However, there are a number of studies on the ecology of palm fungi, which have focused on different aspects of their biodiversity, mainly with regard to saprobes and endophytes. Although the approach of these studies is different, the description of new taxa has often resulted from initially ecological approaches that yielded several interesting fungi for further analysis. This suggests that both approaches are essential to explore the knowledge on fungi and their biodiversity. In fact, the implications of biodiversity and ecological data on the global numbers of fungi and their general knowledge have been pointed out, which will be discussed later in relation to palm fungi and their key role in biodiversity surveys.

A review of the literature on the ecology of palm fungi reveals that studies have been conducted over the past three decades. These have mainly resulted from the intensive research carried out by Hyde and his co-workers. They have made substantial contributions to knowledge of both the taxonomy and biodiversity of palm fungi, not only by identifying the fungal assemblage that occur on palms, but also by exploring the extent of their diversity and the factors that affect it. However, some ecological studies have also been carried out on Indian palm fungi, as well as palm fungi from Central American regions. A historical account of research into the ecology and biodiversity of palm fungi is presented herein.

Fröhlich and Hyde [10] studied the biodiversity of palm fungi in the tropics, forecasting that the estimate of 1.5 million species would be a “very conservative estimate of the number of fungal species extant on the planet”. Taylor et al. [12] investigated the biogeographical distribution of microfungi from temperate and tropical palms. They revealed that differences in fungal assemblages were more related to climatic influences than to the hosts sampled, as well as to the status of these hosts at the site sampled. Subsequently, Yanna et al. [13,14,634] assessed the composition of palm fungal communities and their succession over time, pointing out that differences in fungal assemblages could be related to different collection sites, hosts, stages of decomposition, and tissues sampled. Later, Pinnoi et al. [16] studied saprotrophic fungal communities associated with *Calamus* spp. and reported differences in the assemblages of fungi inhabiting different microhabitats and tissues, with dry petioles supporting a greater diversity of species.

Several studies have been dedicated to endophytic palm fungi, which was one of the first ecological issues to be investigated in palm fungi [10,11,468,469,472,473,635,636,637,638]. These studies often report significant differences in the number of isolates and the taxonomic composition of fungi in respect to the plant growth stages, season, site, and tissues sampled, not only in tropical palms, but also in temperate palms, such as *Trachycarpus fortunei* [215]. Even so, research into palm endophytes, which began in the early 1990s, was primarily motivated by the lack of knowledge about endophytes in plants from tropical regions when compared with those in plants from temperate regions [472,473].

The first study on palm endophytes was by Rodrigues and Samuels [469], who documented the occurrence of endophytes inhabiting the leaves of the Australian fan palm *Licuala ramsayi*, pointing out preliminary differences in the endophyte assemblages recovered from different parts of *L. ramsayi* leaves. In addition, the existence of a fungal assemblage composed mainly of xylariaceous fungi was documented. This was also later documented by Rodrigues and her colleagues when they published the first studies on the fungal endophytes that inhabit the foliage of the Amazonian palm *Euterpe oleracea* from Combu Island (Belém, Pará). In fact, some of these studies were based on systematic and descriptive taxonomy, particularly for xylariaceous species commonly recorded as endophytes of palm tissues, including *Xylaria* and *Idriella* [291,470,471]. However, a comprehensive ecological approach was conducted by Rodrigues [468], who found that colonisation of *E. oleracea* by endophytes was positively correlated with leaf age, plant growth stages, site, and season, as well as with the interactive effect of some of these factors. Later, a similar study was carried out by Fröhlich et al. [11], who investigated the endophyte communities inhabiting different parts of the fronds of *L. ramsayi* individuals from Australia and Brunei. They noted differences between the fungal assemblages of different palm tissues and tissues with different ages. Likewise, Taylor et al. [215] observed differences in the endophytic communities recovered from different tissues of the frond of the temperate palm *Trachycarpus fortunei*. They also documented important information regarding the effect of climate on endophytes assemblages by analysing individuals growing inside and outside their natural geographical range.

The importance of these studies and their implications for fungal biology and biodiversity rapidly increased interest in unveiling more details of the ecology of palmicolous endophytes. Molecular data began to be applied in such studies and made endophytes one of the main issues of palm fungi to be explored until recent years [523,639,640,641,642,643,644,645,646,647,648]. Subsequently, the array of questions has diversified greatly. While some studies have invested in documenting the maximum possible diversity of palm endophytes communities, others have targeted particularly important palms and the impacts that endophytic communities can have on their pests and diseases. For instance, Guo et al. [639,640,641] tried to decrease the percentage of sterile mycelium that often lacks identification in endophyte studies by using both morphology and rDNA sequences in a study of endophytes on *Livistona chinensis* fronds. Later, Rungjindamai et al. [643] and Pinruan et al. [523] used both morphology and molecular techniques to characterise basidiomycete endophytes isolated from the leaves, rachides, and petioles of the oil palm *Elaeis guineensis*, and to reflect on how these fungi can be used as a biocontrol management strategy against the palm pathogen *Ganoderma boninense*. Similarly, Mahmouda et al. [644] examined the endophytic fungal diversity associated with the roots of the date palm *Phoenix dactylifera* growing in coastal dunes to generate a collection of strains that can be used as biocontrol agents against date palm root diseases. More recently, Azuddin et al. [646] studied the fungal endophytes on the spines of *Calamus castaneus* and evaluated their antagonistic activity against phytopathogens.

Although the importance of palm pathogens and their management are often mentioned, particularly in palms that are important in international trade, relatively few studies have focused on these fungi and their ecology, e.g., refs. [199,212]. Most publications on palmicolous pathogens have been motivated by systematic and descriptive taxonomic studies that have identified new taxa associated with palm diseases, e.g., refs. [187,195,196,197,198,208,295,556,630,631,632,633]. In fact, although the main fungal diseases that affect palm trees have virtually all been identified, e.g., refs. [649,650,651] little is known about minor pathogens, including those that cause leaf spots, as shown by the investigation of Hyde and co-workers. Recently, Douanla-Meli and Scharnhorst [551], while describing botryosphaeriaceous taxa associated with palm foliage from Mexico, reflected on the risk these taxa may pose to temperate countries, since palm foliage can be an import route for potentially phytopathogenic fungi. In addition, a number of studies have addressed the identification of fungal pathogens associated with *P. dactylifera* in regions where this palm is an important agricultural crop, e.g., refs. [652,653,654,655,656].

Ecological studies on palm fungi are often directed at palm species that are important world crops in international trade. For example, Asensio et al. [657] investigated the mycobiota of the phylloplane of the date palm *P. dactylifera* and their interactions. Kirkman et al. [658] studied the diversity and ecological association of the oil palm *E. guineensis* fungal microbiome across root, rhizosphere, and soil compartments, while Seephueak et al. [659] studied the diversity of microfungi that occur in different tissues of the oil palm frond litter in a plantation in Southern Thailand. Very few studies on palm fungi have explored fungal diversity in roots and, as a result, palm root fungi are virtually unknown. However, few ecological studies on palm fungi have addressed the biodiversity of arbuscular mycorrhizal fungi (AMF) and reflected on the importance of these symbiotic associations to the health and growth of some palm species, such as *Attalea speciosa* [660], *Coccothrinax* spp. [661,662,663], *Cocos nucifera* [664], *Desmoncus orthacanthos* [665], *Metroxylon sagu* [666], and *P. dactylifera* [667,668].

Many ecological studies have focused on the biodiversity of fungi from peat swamp [248,249,669,670] and mangrove palms [7,162,671,672,673,674,675]. Such studies often reflect on the assemblage of freshwater and marine fungi that inhabit palm tissues, compared with the fungi typically associated with terrestrial palms. In addition, the presence of different fungal assemblages from different microhabitats and palm trees tissues is often addressed. For instance, Pinnoi et al. [248], studying the fungal biodiversity of *Eleiodoxa conferta*, reported that fungi are more abundant on the petioles of wet palm material. Similar ecological patterns were reported by Pinruan et al. [249], who indicated that the petioles of dry material of *Licuala longicalycata* supported the most diverse fungal communities. Furthermore, questions regarding host- and tissue-specificity are frequently addressed, particularly in studies on the brackish water palm *Nypa fruticans*, whose colonisation by fungi has been well documented [671,674]. Hyde and Alias [7] reported differences in the fungal composition of different palm structures of *N. fruticans*, including leaves, leaf veins, rachides, petioles, and inflorescences, collected from intertidal and terrestrial habitats. Likewise, Hyde and Sarma [672] reported differences in the fungal assemblages inhabiting *N. fruticans* along a river, addressing some ecological observations regarding the horizontal and vertical distribution of fungi. One of the most recent publications in the ecology of palmicolous fungi reported the co-occurrence of certain species of fungi on *N. fruticans*. This study revealed some aspects related to the structure of the fungal communities on tissues of the brackish water palm and helped to understand the dynamics of the ecosystem, suggesting, for example, the potential interaction established between the fungi of these communities [675].

There are numerous studies on palm fungi and a plethora of data has been collected on different aspects of their biodiversity—from their taxonomic and systematic structure to ecological traits of their lifestyles inhabiting different palm tissues, microhabitats, and geographic regions. However, review studies on palm fungi are scarce. Most are confined to proceedings of mycological conferences or chapters in books that explore broader themes, e.g., refs. [17,676,677,678]. With the advent of DNA sequencing, information on palm fungi, particularly their taxonomy and systematics, has become considerably more complex. Thus, defining or understanding them as a complex and diverse group of fungi may not be an easy task. In this sense, the question “what are palm fungi?” arose and will be discussed below.

## 3. What Are Palm Fungi? Global Figure and Taxonomic Structure

Palm fungi have been widely documented in Australia, Brunei, Ecuador, Hong Kong, Thailand and, to a lesser extent, in Cuba, Mexico, and India (Figure 1). The data obtained indicate that palm fungi are undoubtedly a taxonomically diverse group. However, their precise taxonomic structure within a natural phylogenetic framework still requires further studies, especially those using DNA sequence data. Many publications have outlined the studies that have been carried out on palm fungi, e.g., refs. [8,17,104,676,677,678]. Nonetheless, to date, there is no recent comprehensive review on this group of fungi. Therefore, most of the figures presented are outdated and uncertain, as they are only based on the intensive research carried out in the 1990s.

### 3.1. Global Figure of Palm Fungi

According to Hyde et al. [17], by 1997 the global figure of palm fungi was ca. 1580 species, including 650 ascomycetes (41%), 270 basidiomycetes (17%), and 660 “anamorphs” (42%), i.e., 400 hyphomycetes (25%) and 260 coelomycetes (17%). However, given the intensive research carried out since then, it is easy to see that these figures are considerably outdated. In fact, the extensive studies carried out by Hyde and co-workers from the early 1990s to the present day have documented ca. 500 new taxa from palms, almost all of them ascomycetes (the term ascomycetes is used here to refer to species of *Ascomycota* reported through the presence of their sexual morphs in *Arecaceae* host tissues), in addition to a number of new host records for *Arecaceae*. Moreover, several new taxa and host records have been reported from other research groups, including, for example, the palmicolous hyphomycetes collected from palms in Cuba and Mexico by Castañeda-Ruiz, Holubová-Jechová, Mena-Portales, Mercado-Sierra, and many other co-workers.

In view of the present comprehensive review of the literature, it is assumed that the global figure of palm fungi is more than 2500 species. Specifications about each group of fungi, i.e., ascomycetes, basidiomycetes, and asexual morphs, must be carefully made. The global figure produced by Hyde et al. in the 1990s noted that around 41% of the fungi described on palms were ascomycetes [17]. As most of their reports since then were ascomycetes, it can be forecasted that the proportion of ascomycetes in the global figure of palm fungi should be higher, ca. 1370 (55%) species. Similarly, Hyde et al. [17] noted that around 42% of the fungi described from palms were asexual morphs. However, research since then has not revealed as many palmicolous “anamorphs” as ascomycetes, so it is considered that this proportion should be lower, ca. 870 (35%) species. As almost no basidiomycete has been described from palms since the investigation carried out by Hyde et al. in the 1990s, it is considered here that the number of basidiomycetes in the global figure of palm fungi is still ca. 270 (10%) species.

Although these numbers may seem nonsensical, trying to pinpoint them has a great impact on our understanding of the taxonomic structure of palm fungi. In turn, they can have implications for the way mycology studies and searches for them, especially when trying to answer central biodiversity questions, such as “where are the missing fungi?” or “how many fungi are there?”. Before diving into what is currently considered to be the ecology and taxonomy of palm fungi, a few considerations should be made.

*The lack of molecular data*. As previously mentioned, most studies carried out on palm fungi have been exclusively morphological, which means that most of the species, and higher taxonomic ranks, described have not considered molecular data. As is well known, combining molecular data with morphology is essential for establishing a natural phylogenetic framework. Morphology alone is subjective and can mislead species identification or incorrectly assign them to higher taxonomic ranks, disrupting their true identity and phylogenetic relationships. Furthermore, due to the phenotypic plasticity of morphological traits, the global figure of palm fungi is likely to be underestimated or overestimated. The recollection, epitypification, and isolation of these fungi is critical to establish a natural taxonomic framework for palm fungi.

*The lack of studies on palmicolous “anamorphs”*. Very few studies on palm fungi have focused on asexual morphs, especially coelomycetes. In fact, most studies on palm fungi have studied the *Ascomycota* coverage through the presence of its sexual morphs on the hosts. Thus, it is not surprising that palmicolous “anamorphs” are less represented in the global figure of palm fungi than ascomycetes “teleomorphs”. Recent reports predicted that the number of asexually reproducing fungi is greater than the number of sexually reproducing fungi [590]. It can therefore be predicted that palmicolous “anamorphs” are highly understudied and, consequently, underestimated. Since palms are fungi-rich host plants, it is expected that many new asexually reproducing species of palm fungi are awaiting to be documented. Furthermore, the above-mentioned numbers clearly demonstrate how far from the truth the global figure of palm fungi is, since more *Ascomycota* “teleomorphs” have been recorded on palms than “anamorphs”.

*The lack of studies on palmicolous basidiomycetes*. As with palmicolous “anamorphs”, very few studies on palm fungi concern basidiomycetes. Although it can be assumed that their proportion in palm fungal assemblages is low, given the previous studies that have treated these fungi as communities through their isolation in culture (traditional methodology), almost no study has specifically aimed to evaluate palm basidiomycetes. For example, Pinruan et al. [523] studied the occurrence and diversity of endophytic and saprophytic basidiomycetes on the oil palm *Elaeis guineensis* in Thailand and confirmed a rich fungal diversity. As the authors discuss, traditional isolation methods are known to hinder the detection of basidiomycetes. Especial concerns about the selectivity of the methods and the temporal and spatial variability of the basidiomycetes’ mycelium should be considered when studying these fungi [523]. Thus, the lack of studies aimed specifically at palmicolous basidiomycetes can be the reason why the proportion of these fungi is so low in the global figure of palm fungi. It can therefore be predicted that palmicolous basidiomycetes are also highly understudied and underestimated.

*The difficulty of compiling data*. Predicting an exact global figure of palm fungi and their specific groups is a difficult task. Many studies are constantly being published on new species documented on palm substrata and it can be difficult to keep track of them all. Similarly, an overwhelming number of studies have reported on palm fungi by randomly studying certain groups of fungi, hosts, or habitats and compiling these reports is an almost impossible task. Furthermore, the extent of these publications and, consequently, the rich diversity of palm fungi, makes it impossible to use well-known databases such as the US National Fungus Collections Fungus–host Database, which often cannot cope with the number of reports due to the enormous amount of information on palm fungi.

### 3.2. Taxonomic Structure of Palm Fungi

This overview summarises the main taxa found on palm trees, which typically make up what is referred to as palm fungi. Although the expression “palm fungi” has been used here to denote records of fungi on *Arecaceae* hosts, it was originally applied to a particular mycota that was consistently found in association with palms in the tropics. Thus, this overview does not represent an exhaustive list of taxa that have been reported from *Arecaceae* at any taxonomic rank (i.e., species, genera, or higher taxonomic ranks). As a result, some genera already recorded on palms may not be mentioned either because their frequency is not particularly significant, or mainly because they represent ubiquitous and plurivorous taxa, which are associated with several different hosts and present a cosmopolitan distribution (e.g., *Alternaria*, *Aspergillus*, *Penicillium*, and *Phoma*). The taxonomic structure of palm fungi presented here follows the most recent taxonomic updates and phylogenetic treatments available in the literature (i.e., refs. [313,314,596,621,627,679,680,681,682,683,684,685,686,687,688,689,690,691,692]).

Palm fungi are a taxonomically diverse group with more than 2500 species, including representatives of all the major classes of the fungal kingdom (Table 2). The most representative group of palm fungi is the ascomycetes, a diverse assemblage in which the best represented class is the *Sordariomycetes*, with four commonly recorded genera, namely *Anthostomella* (*Xylariaceae*, *Xylariales*)*, Linocarpon* (*Linocarpaceae*, *Chaetosphaeriales*), *Oxydothis* (*Oxydothidaceae*, *Amphisphaeriales*), and *Phomatospora* (*Phomatosporaceae*, *Phomatosporales*). According to Hyde [111], these correspond to the main genera that invariably colonise fallen palm rachides and leaves in the tropics.

#### 3.2.1. Palmicolous Sordariomycetes

Since the extensive studies carried out by Hyde and co-workers in the 1990s, it has become clear that xylarialean fungi are commonly encountered on palm hosts. In fact, the most well-represented order and family of palm fungi is *Xylariales* and *Xylariaceae*, respectively. However, several species of many xylarialean genera still lack molecular data. Thus, to predict their taxonomic structure in *Xylariaceae* and allied families is often difficult and based in subjective approaches (i.e., comparison of morphological characters). In turn, many of these genera are placed in *Xylariales incertae sedis* and, therefore, the taxonomic structure of several xylarialean genera of palm fungi is still obscure and needs molecular-based studies [681,682,683].

In addition to *Anthostomella*, a number of xylarialean genera have been recorded on palms, including *Astrocystis*, *Kretzschmaria*, *Nemania*, *Rosellinia*, *Stilbohypoxylon*, *Xylaria* (*Xylariaceae*), *Biscogniauxia* (*Graphostromataceae*), *Idriella* (*Microdochiaceae*), and *Hypoxylon* (*Hypoxylaceae*), e.g., ref. [693]. However, according to Smith and Hyde [150], although palm litter is a major component of many lowland rainforests, comparatively few of most of these xylarialean fungi seem to exploit this substratum. Yet, some genera are exceptions to the rule and, in addition to *Anthostomella*, Hyde and co-workers found that several other xylarialean fungi are common on collections of rainforest palms, particularly clypeosphaeriaceous and diatrypaceous fungi, e.g., refs. [134,263,694]. These include *Annulohypoxylon* (*Hypoxylaceae*), *Apioclypea*, *Brunneiapiospora*, *Palmaria* (*Clypeosphaeriaceae*), *Allocryptovalsa*, *Allodiatrype*, *Anthostoma, Cryptovalsa*, *Diatrype*, *Diatrypella*, *Eutypa*, *Eutypella*, *Frondisphaeria*, *Peroneutypa* (*Diatrypaceae*), *Arecophila*, *Seynesia*, *Endocalyx* (*Cainiaceae*), *Fasciatispora* (*Fasciatisporaceae*), *Neoxylaria* (*Xylariaceae*), *Zygosporium* (*Zygosporiaceae*) and many other genera, such as *Capsulospora*, *Circinotrichum*, *Cyanopulvis*, *Guestia*, *Haploanthostomella*, *Lasiobertia*, *Nipicola*, *Palmicola*, *Pemphidium*, *Pulmosphaeria*, and *Sabalicola* (*Xylariales* genera *incertae sedis*). Several of these genera are found exclusively or almost exclusively on palms and represent morphological genera, whose phylogenetic resolution is still needed for a precise taxonomic structuring of palm fungi, e.g., ref. [539].

Many other *Sordariomycetes* occur on palms and many genera are considered to be typical in the tropical assemblage of palm fungi, especially in *Amphisphaeriales*, *Chaetosphaeriales*, *Meliolalles*, *Phyllachorales*, and, to a lesser extent, *Sordariales* and *Diaporthales*.

In *Amphisphaeriales*, apart from *Oxydothis* (*Oxydothidaceae*), which is the most commonly found genus of palm fungi, e.g., ref. [528], a great diversity of taxa has been recorded on palms, particularly in *Amphisphaeriaceae*, e.g., ref. [136], *Hyponectriaceae*, e.g., ref. [138], and *Apiosporaceae*, e.g., refs. [125,540]. Several of these genera, like the xylarialean genera mentioned, are almost exclusively palm taxa and include only morphological species, so their phylogenetic resolution is still needed. *Amphisphaeriales* members occurring on palms include *Amphisphaeria*, *Lepteutypa* (*Amphisphaeriaceae*), *Arecomyces*, *Frondicola*, *Hyponectria*, *Rachidicola* (*Hyponectriaceae*), *Appendicospora* (*Appendicosporaceae*), *Arthrinium*, *Dictyoarthrinium* (*Apiosporaceae*), *Iodosphaeria* (*Iodosphaeriaceae*), and *Leiosphaerella* (*Pseudomassariaceae*). In addition to members of *Apiosporaceae* and *Appendicosporaceae*, several genera of ascomycetes with unitunicate asci and apiospores are often found on palm tissues [143]. These include some of the xylarialean genera mentioned above (i.e., *Anthostomella*. *Apioclypea*, *Brunneiapiospora*, *Palmaria*) and other amphisphaeriaceous members, such as *Pseudomassaria* (*Pseudomassariaceae*).

In *Chaetosphaeriales*, the genera *Linocarpon*, *Neolinocarpon* (*Linocarpaceae*), and *Leptosporella* (*Leptosporellaceae*) are often found on palms, and their natural placements are beginning to be resolved as new collections are made, although they are still poorly represented with sequence data, e.g., ref. [530]. In addition to these three genera, other *Chaetosphaeriales* genera commonly recorded on palms include *Chaetosphaeria*, *Chloridium*, *Sporoschisma* (*Chaetosphaeriaceae*), and *Caudatispora* (*Chaetosphaeriales* genus *incertae sedis*), e.g., ref. [695].

In *Phyllachorales*, most of the members that occur in association with palms are in *Phaeochoraceae*, which was introduced to accommodate saprotrophic or biotrophic ascomycetes on plant leaves apparently restricted to *Arecaceae* hosts, including the genera *Cocoicola*, *Phaeochora*, *Phaeochoropsis*, and *Serenomyces*, e.g., refs. [212,696]. These genera of *Phaeochoraceae*, along with members of *Phyllachoraceae*, including *Brobdingnagia*, *Camarotella*, *Coccodiella*, *Coccostromopsis*, *Maculatifrondes*, *Malthomyces*, *Ophiodothella*, *Oxodeora*, *Phyllachora*, *Sphaerodothis* and *Tribulatia*, and *Catabotrys* (*Catabotryaceae*, *Catabotryales*), accommodate species that cause tar spots or lesions on palm leaves and can cause substantial diseases in the hosts, e.g., refs. [212,697].

In *Meliolalles*, species of *Meliola* (*Meliolaceae*) are commonly represented as pathogens on palms, e.g., ref. [6]. In *Sordariales*, members occurring in association with palms are essentially represented by *Cercophora*, *Lasiosphaeria* (*Lasiosphaeriaceae*) and *Lockerbia* (*Sordariales* genus *incertae sedis*), e.g., ref. [185].

Most of the previously discussed taxa commonly found on palms are represented in two subclasses of the *Sordariomycetes*, namely *Xylariomycitidae* and *Sordariomycetidae*. However, a great diversity of *Sordariomycetes* is collected from palms and dispersed among many other taxonomic ranks, including a number of members of the *Diaporthomycetidae*, such as the frequently reported palmicolous genus *Phomatospora* (*Phomatosporaceae*, *Phomatosporales*), and *Hypocreomycetidae*, such as genera in the *Microascales*, viz. *Triadelphia* (*Triadelphiaceae*) and *Wardomycopsis* (*Microascaceae*), as well as other examples that will be discussed below, e.g., ref. [110].

Some genera of the *Sordariomycetes* recorded on palms are represented by a single or a few collections and their phylogeny is still vague and of uncertain placement within subclasses or orders, for example, *Arecacicola*, *Curvatispora*, *Nigromammilla*, *Paracapsulospora*, *Mangrovispora* (*Sordariomycetidae* genera *incertae sedis*), *Cannonia* (*Coniochaetales* genus *incertae sedis*), *Frondispora*, *Manokwaria* (*Xylariomycitidae* genera *insertae sedis*), *Myelosperma* (*Myelospermataceae*, *Xylariomycetidae* family *incertae sedis*), *Neobarrmaelia* (*Xylariales* genus *incertae sedis*), and *Thyridium* (*Thyridiaceae*, *Diaporthomycetidae* family *incertae sedis*). Thus, their recollection and epitypification is crucial to establish and clarify their natural placements among extant well-known taxa of *Sordariomycetes* [681]. In some cases, such as *Paracapsulospora* and *Neobarrmaelia*, its natural placement is unclear due to the limited sequence data available to populate surrounding clades [292,296]. Less frequently, members of other subclasses of *Sordariomycetes* are also reported from palm hosts. For example, members of *Conioscyphales*, *Pleurotheciales*, and *Savoryellales* (*Savoryellomycetidae*), including the hyphomycete genera *Conioscypha* (*Conioscyphaceae*) and *Monotosporella* (*Pleurotheciaceae*), and *Ascotaiwania*, *Canalisporium*, and *Savoryella* (*Savoryellaceae*), respectively, have occasionally been found on palms, e.g., refs. [619,698,699].

In *Diaporthales*, the members that occur in association with palms are essentially represented by *Diaporthe* (*Diaporthaceae*) and mostly restricted to typically temperate palms, although no study has yet dealt with their diversity in depth, e.g., ref. [276]. Other genera of *Diaporthales* have been recorded on palms, but are represented by single, old collections, whose reanalysis and/or recollection is necessary to properly resolve their phylogeny, namely *Apiosphaeria* (*Diaporthaceae*), *Coniella* (*Schizoparmaceae*), *Maculatipalma* (*Gnomoniaceae*), *Durispora*, and *Phruensis* (*Diaporthales* genera *incertae sedis*), e.g., ref. [143].

Many *Nectria*-like and allied species from a wide range of genera are found on palms. These include several members of the *Hypocreales*, such as *Calonectria*, *Chaetopsina*, *Cosmospora*, *Dactylonectria*, *Ilyonectria*, *Nectriopsis*, *Nectria*, *Ophionectria*, *Pleiocarpon*, *Volutella* (*Nectriaceae*), *Clonostachys*, *Hydropisphaera*, *Ijuhya*, *Lasionectria*, *Nectriella* (*Bionectriaceae*), *Niesslia* (*Niessliaceae*), *Stachybotrys* (*Stachybotryaceae*), *Trichoderma*, and *Verticimonosporium* (*Hypocreaceae*), as well as members of the *Magnaporthales*, such as *Gaeumannomyces* (*Magnaporthaceae*), *Ophioceras* (*Ophioceraceae*), *Pyricularia* (*Pyriculariaceae*), and *Pseudohalonectria* (*Pseudohalonectriaceae*), e.g., refs. [200,207,592]. Likewise, many *Acremonium*-like fungi and related genera have common representatives on palms, some of which have recently been introduced, including members in *Hypocreales*, such as *Neoacremonium* (*Neoacremoniaceae*), *Acremonium*, *Gossypinidium*, *Hydropisphaera*, *Lasionectriella*, and *Paracylindrocarpon* (*Bionectriaceae*), and *Glomerellales*, such as *Brunneomyces* and *Acremoniisimulans* (*Plectosphaerellaceae*), e.g., refs. [310,582].

In *Tirisporellales*, all members are freshwater ascomycetes described from palms, including *Bacusphaeria*, *Thailandiomyces*, and *Tirisporella* (*Tirisporellaceae*), e.g., ref. [599]. In *Annulatascales*, the genera *Annulatascus* and *Submersisphaeria* (*Annulatascaceae*), which are typical freshwater taxa, have representatives collected from terrestrial and intertidal palm samples, e.g., ref. [247].

Several genera with common representatives on terrestrial palms also have some marine and freshwater palmicolous species. These have often been recorded on intertidal samples of Nipa palms, which can be wetted daily by tidal inundations and consequently colonised by marine fungi. These include species of *Anthostomella*, *Fasciatispora*, *Linocarpon*, *Neolinocarpon*, *Nipicola*, *Oxydothis*, and *Phomatospora*, e.g., ref. [110]. In addition to genera typically associated with terrestrial palm samples and members of *Tirisporellaceae* and *Annulatascaceae*, a number of aquatic *Sordariomycetes* are commonly found colonising mangrove and peat swamp palms. These include the halosphaeriaceous genera *Aniptodera*, *Fluviatispora*, and *Lignincola* (*Halosphaeriaceae*, *Microascales*), *Baipadisphaeria* (*Nectriaceae*, *Hypocreales*), *Flammispora* (*Sordariomycetes* genus *incertae sedis*), *Savoryella* (*Savoryellaceae*, *Savoryellales*), *Trichocladium* (*Chaetomiaceae*, *Sordariales*), and *Unisetosphaeria* (*Trichosphaeriaceae*, *Diaporthomycetidae* family *incertae sedis*). Many other genera, whose phylogeny within the ascomycetes is still unresolved, are reported from freshwater and intertidal palm samples, such as *Nypaella* and *Helicorhoidion* (*Ascomycota* genera *incertae sedis*), e.g., refs. [166,700].

#### 3.2.2. Palmicolous *Dothideomycetes*

Apart from the above-mentioned genera, most aquatic palmicolous fungi are representatives of the *Dothideomycetes* [596,686], including several members of *Pleosporales*, some of which also include terrestrial species, viz. *Acuminatispora*, *Plectophomella* (*Pleosporales* genera *incertae sedis*), *Astrosphaeriella* (*Astrosphaeriellaceae*), *Carinispora* (*Pseudoastrosphaeriellaceae*), *Falciformispora*, *Trematosphaeria* (*Trematosphaeriaceae*), *Helicascus* (*Morosphaeriaceae*), *Herpotrichia* (*Melanommataceae*), *Leptosphaeria* (*Leptosphaeriaceae*), *Lolia* (*Lindgomycetaceae*), *Massarina* (*Massarinaceae*), and *Salsuginea* (*Salsugineaceae*), e.g., refs. [116,166]. Recently, the new pleosporalean family *Striatiguttulaceae* was established to accommodate two new manglicolous fungi from palms, *Longicorpus* and *Striatiguttula* [9]. Moreover, other members of *Dothideomycetes* include aquatic representatives from palm samples, such as members in *Jahnulales*, including *Jahnula* (*Aliquandostipitaceae*) and *Manglicola* (*Manglicolaceae*).

In recent years, a series of *Dothideomycetes* have been frequently described from palm trees based on morpho-molecular data [687,688]. This, in turn, has clarified the structure of the taxa of *Dothideomycetes* that make up the assemblage of palm fungi and the best represented order is *Pleosporales*. Some of these taxa are part of the genera most frequently found on palm hosts, particularly the *Astrosphaeriella*-like species. The polyphyletic nature of *Astrosphaeriella sensu lato* has recently been resolved in different families and/or genera to include typically palmicolous taxa. *Astrosphaeriella*-like species on palms include different genera in three families, viz. *Astrosphaeriella*, *Astrosphaeriellopsis*, *Pteridiospora*, *Pithomyces*, *Javaria*, *Xenoastrosphaeriella* (*Astrosphaeriellaceae*), *Fissuroma*, *Neoastrosphaeriella* (*Aigialaceae*), and *Pseudoastrosphaeriella* (*Pseudoastrosphaeriellaceae*), e.g., refs. [267,533,701]. Moreover, members in *Botryosphaeriales* are also found on palms, including *Barriopsis*, *Botryosphaeria*, *Diplodia*, *Lasiodiplodia*, *Neodeightonia* (*Botryosphaeriaceae*), and *Phyllosticta* (*Phyllostictaceae*), and their potential as phytopathogens has occasionally been discussed, e.g., refs. [549,550,702,703]. Likewise, members of the *Occultibambusaceae* have recently been recorded in palms collections, including *Brunneofusispora* and *Neooccultibambusa* [537].

Several other *Dothideomycetes* are commonly found on palms and mostly reside in *Pleosporales*, especially in the *Didymosphaeriaceae*, such as *Didymosphaeria*, *Montagnula*, *Paraconiothyrium*, *Paraphaeosphaeria*, *Pseudopithomyces*, and *Spegazzinia*, and *Roussoellaceae*, such as *Appendispora*, *Neoroussoella*, and *Roussoella*, e.g., refs. [222,223,534]. Some of these genera are part of one of the most interesting groups of *Dothideomycetes* found on palms, as they include truly terrestrial ascomycetes with extracellular, often gelatinous appendages on the ascospores, which are mainly known from aquatic habitats, e.g., ref. [147]. However, many other pleosporalean fungi have been recorded on palms from a wide range of families. These include members of the *Coniothyriaceae*, such as *Coniothyrium*; *Delitschiaceae*, such as *Delitschia*; *Lophiostomataceae*, such as *Lophiostoma* and *Vaginatispora*; and *Neophaeosphaeriaceae*, such as *Neophaeosphaeria*, e.g., ref. [616]. Other members of *Pleosporales* found on palm include the genus *Corynesporasca* (*Corynesporascaceae*), which is a morphological genus introduced based on palm collections. Although it has been shown that *Corynesporasca* has a *Corynespora*-like asexual morph, the phylogenetic relationships of these two genera are unclear until molecular data of the type species are available [275].

#### 3.2.3. Palmicolous “Anamorphs”

In addition to the previously mentioned anamorphic taxa, many families and members of *Pleosporales* that include asexually reproducing fungi, especially hyphomycetes, are typically found on palms. These include members of the *Dictyosporiaceae*, such as *Dictyocheirospora*, *Dictyopalmispora*, *Dictyosporium*, *Pseudocoleophoma*, and *Sporidesmiella*, e.g., ref. [602]; *Hermatomycetaceae*, including *Hermatomyces*; *Leptosphaeriaceae*, including *Chaetoplea* and *Quasiphoma*, e.g., refs. [569,573]; *Massarinaceae*, including *Haplohelminthosporium*, *Helminthosporiella*, and *Helminthosporium*, e.g., ref. [285]; *Melanommataceae*, including *Asymmetricospora*, *Byssosphaeria*, *Camposporium*, and *Herpotrichia*, e.g., refs. [575,704]; *Periconiaceae*, including *Periconia*, e.g., ref. [705]; *Phaeosphaeriaceae*, including *Amarenographium*, *Parastagonospora*, *Phaeosphaeria*, *Septoriella*, and *Wojnowiciella*, e.g., ref. [292]; *Pleosporaceae*, including *Bipolaris*, *Curvularia*, and *Exserohilum*, e.g., ref. [706]; *Pseudoberkleasmiaceae*, namely *Pseudoberkleasmium*, e.g., ref. [576]; *Teichosporaceae*, including *Parateichospora*, e.g., ref. [299]; and *Tetraplosphaeriaceae*, including *Ernakulamia* and *Tetraploa*, e.g., ref. [541]; as well as other taxa, for instance, the genus *Repetophragma* (*Pleosporales* genus *incertae sedis*). Moreover, species of *Torula*-like genera are also frequently recorded on palms from tropical countries, including *Bahusandhika* (*Lentimurisporaceae*), *Cylindrotorula*, and *Torula* (*Torulaceae*), e.g., ref. [587].

Studies on palmicolous “anamorphs” are scarce and are mostly restricted to dematiaceous hyphomycetes and botryosphaeriaceous coelomycetes, which seem to be an important assemblage of fungi that inhabit palm tissues, especially in the tropics. However, several palmicolous “anamorphs” have recently been described and introduced in mycological series as occasional discoveries. In turn, the taxonomic structure of palmicolous “anamorphs” is becoming better known and more complex, including many *Sordariomycetes* and particularly *Dothideomycetes*.

Concerning *Sordariomycetes*, the genera *Ascotaiwania*, *Canalisporium* (*Savoryellaceae*, *Savoryellales*), *Distoseptispora* (*Distoseptisporaceae*, *Distoseptisporales*), *Monotosporella* (*Pleurotheciaceae*, *Pleurotheciales*), *Melanconis*, *Melanconium* (*Melanconidaceae*, *Diaporthales*), *Melanographium* (*Sordariomycetes* genus *incertae sedis*), *Pararamichloridium* (*Pararamichloridiaceae*, *Pararamichloridiales*), and *Spadicoides* (*Xenospadicoidaceae*, *Xenospadicoidales*) are some of the hyphomycetes typically recorded on palms, e.g., refs. [15,238,612].

In addition, some of the most common *Sordariomycetes* taxa found on palms also include palmicolous “anamorphs”. These comprise several members of the *Xylariales*, including the genera *Ascotricha*, *Diabolocovidia* (*Xylariaceae*), *Barrmaelia* (*Barrmaeliaceae*), *Circinotrichum*, *Gyrothrix* (*Xylariales* genera *incertae sedis*), *Endocalyx* (*Cainiaceae*), *Hansfordia* (*Hansfordiaceae*), *Microdochium* (*Microdochiaceae*), and *Zygosporium* (*Zygosporiaceae*), e.g., refs. [546,562]. Likewise, several anamorphic fungi of the *Chaetosphaeriales* are recorded on palms, including *Chloridium*, *Codinaea*, *Craspedodidymum*, *Cryptophiale*, *Dictyochaeta*, *Kionochaeta*, *Rattania*, *Sporoschisma*, *Thozetella (Chaetosphaeriaceae*), and *Endophragmiella* (*Helminthosphaeriaceae*), e.g., refs. [243,707]. Moreover, a plethora of asexual *Hypocreales* genera have common representatives on palm hosts, such as *Acremonium*, *Gossypinidium*, *Hydropisphaera*, *Lasionectriella*, *Paracylindrocarpon* (*Bionectriaceae*), *Chaetopsina*, *Dactylonectria*, *Pleiocarpon*, *Volutella* (*Nectriaceae*), *Neoacremonium* (*Neoacremoniaceae*), *Alfaria*, *Stachybotrys*, and *Virgatospora* (*Stachybotryaceae*), e.g., refs. [283,317]. In addition, species of *Fusarium* (*Nectriaceae*) are also common on palm hosts and have been associated with important diseases, e.g., ref. [708]. Other *Sordariomycetes* orders with palmicolous asexual morphs include the *Magnaporthales* genus *Pyricularia* (*Pyriculariaceae*); the *Microascales* members *Ceratocystis*, *Thielaviopsis* (*Ceratocystidaceae*), *Cirrenalia* (*Halosphaeriaceae*), *Custingophora* (*Gondwanamycetaceae*), *Triadelphia* (*Triadelphiaceae*), and *Wardomycopsis* (*Microascaceae*); and many other genera, such as *Diaporthe* (*Diaporthaceae*, *Diaporthales*), *Coniella* (*Schizoparmaceae*, *Diaporthales*), *Koorchaloma* (*Trichosphaeriaceae*, *Diaporthomycetidae* family *incertae sedis*), *Paraproliferophorum* (*Diaporthomycetidae* genus *incertae sedis*), and *Pararamichloridium* (*Pararamichloridiaceae*, *Pararamichloridiales*), e.g., refs. [8,226].

Species of pestalotioid fungi in *Amphisphaeriales* have recently been described from palm collections, including some members of the *Sporocadaceae*, such as *Bartalinia*, *Morinia*, *Neopestalotiopsis*, *Pestalotiopsis*, *Pseudopestalotiopsis*, *Robillarda*, and *Seiridium*. Other “anamorphs” in *Amphisphaeriales* with representatives on palms include, for instance, *Beltrania* (*Beltraniaceae*), *Arthrinium*, and *Dictyoarthrinium* (*Apiosporaceae*), e.g., refs. [556,559].

Several other palmicolous “anamorphs” are *Sordariomycetes*, such as the hyphomycetes genera *Acrodictys* (*Acrodictyaceae*, *Sordariomycetes* family *incertae sedis*), *Apogaeumannomyces* (Sordariomycetes genus *incertae sedis*), and *Hyalobelemnospora* (*Ophiostomataceae*, *Ophiostomatales*). Even so, most of palmicolous “anamorphs” are *Dothideomycetes* or, like some of the above-mentioned *Sordariomycetes*, represent morphological, monotypic genera introduced based on palm collections and are known only from palms or almost exclusively from palms and their phylogeny is still uncertain and reside in *Ascomycota* genera *incertae sedis*. These include the hyphomycetes genera *Acarocybellina*, *Acarocybiopsis*, *Agrabeeja*, *Anabahusakala*, *Ashtaangam*, *Atrosetaphiale*, *Basauxia*, *Bhadradriella*, *Botryomonilia*, *Brachysporiopsis*, *Bulbocatenospora*, *Ceratosporella*, *Cheiromyceopsis*, *Consetiella*, *Delortia*, *Dwibahubeeja*, *Endosporoideus*, *Helensiella*, *Hemisynnema*, *Holubovaea*, *Kalamarospora*, *Mackenziella*, *Nusia*, *Sawantomyces*, *Septosporiopsis*, *Setophiale*, *Spiculostilbella*, *Stratiphoromyces*, *Paradactylella*, *Phragmospathulella*, *Polybulbophiale*, *Rogergoosiella*, *Tretendophragmia*, *Tretocephala*, *Venustisporium*, *Venustocephala*, *Veramycella*, *Veramyces*, and *Waihonghopes* (for references and details of some of these genera, see Table 1). The recollection of these taxa is imperative for the knowledge of the assemblage of palmicolous “anamorphs”. Other asexual morphs known from palms that reside in *Ascomycota* genera *incertae sedis* include *Argopericonia*, *Barnettella*, *Bharatheeya*, *Bhatia*, *Capitorostrum*, *Ceratosporella*, *Drepanospora*, *Endomelanconium*, *Everhartia*, *Grallomyces*, *Goidanichiella*, *Haplobasidion*, *Helicoubisia*, *Kostermansinda*, *Lacellina*, *Lomachashaka*, *Lylea*, *Megalodochium*, *Phaeomonilia*, *Podosporium*, *Polytretophora*, *Pseudotorula*, *Sporidesmiopsis*, *Stauriella*, *Staurophoma*, *Tharoopama*, and *Vanakripa*, e.g., refs. [229,236,517].

Numerous *Dothideomycetes* are recorded on palm trees, many of which include asexual morphs. In addition to some of the above-mentioned genera, such as *Cirrenalia*, *Delortia*, *Drepanospora*, *Everhartia*, *Helicorhoidion*, and *Helicoubisia*, many other allied genera of helicosporous hyphomycetes are found in association with palms, including *Hymenoscyphus* (*Helotiaceae*, *Helotiales*), *Xenosporium* (*Dothideomycetes* genus *incertae sedis*), and members of the *Tubeufiaceae* (*Tubeufiales*), such as *Helicoma*, *Helicomyces*, *Helicosporium*, and *Thaxteriella*, along with other non-helicosporous genera, such as *Berkleasmium*, e.g., refs. [43,319,321,324]. Other members in *Tubeufiales* encountered on palms include *Aquaphila* (*Tubeufaceae*) and *Wiesneriomyces* (*Wiesneriomycetaceae*). In *Asterinales*, species of *Asterina*, *Cirsosia*, *Discopycnothyrium* (*Asterinaceae*), *Lembosia* (*Lembosiaceae*), and *Morenoina* (*Morenoinaceae*) were collected from palms, some of which included known pathogens commonly represented on palms, e.g., refs. [152,279,606]. Several palmicolous “anamorphs”, particularly hyphomycetes, with *Mycosphaerella*-like sexual morphs reside in *Mycosphaerellales*, including some species of *Cercospora*, *Distocercospora*, *Exosporium*, *Pallidocercospora*, *Passalora*, *Phaeophleospora*, *Pseudocercospora*, *Ramularia*, *Scolecostigmina*, *Uwemyces*, *Zasmidium* (*Mycosphaerellaceae*), and *Pseudoepicoccum* (*Mycosphaerellales* genus *incertae sedis*), some of which have been recorded on palms associated with foliar diseases, e.g., refs. [630,709,710,711]. Recently *Palmeiromyces* (*Teratosphaeriaceae*) was recorded as an obligate biotroph causing palm leafspots [295]. Other members of *Mycosphaerellales*, especially dematiaceous hyphomycetes, have been recorded on palms, such as *Castanedospora* (*Extremaceae*) and *Stenella* (*Teratosphaeriaceae*), e.g., ref. [273]. Moreover, in *Kirschsteiniotheliales*, the hyphomycetes genera *Kirschsteiniothelia* (*Kirschsteiniotheliaceae*) and *Taeniolella* (*Kirschsteiniotheliales* genus *incertae sedis*) have been recorded on palms, e.g., ref. [618].

**Table 2:** Synopsis of the taxonomic structure of palm fungi: genera and respective families in subclasses of *Sordariomycetes* and *Dothideomycetes* with common representatives found on *Arecaceae* hosts.

Class	Subclass	Order	Family	Genera
*Dothideomycetes*	*Dothideomycetidae*	*Dothideales*	*Dothideaceae*	*Uleodothis*
		*Mycosphaerellales*	*Extremaceae*	*Castanedospora*
			*Mycosphaerellaceae*	*Cercospora*, *Distocercospora*, *Exosporium*, *Pallidocercospora*, *Passalora*, *Phaeophleospora*, *Pseudocercospora*, *Ramularia*, *Scolecostigmina*, *Uwemyces*, *Zasmidium*
			*Teratosphaeriaceae*	*Palmeiromyces*, *Stenella*
			*Incertae sedis*	*Pseudoepicoccum*
	*Pleosporomycetidae*	*Acrospermales*	*Acrospermaceae*	*Gonatophragmium*
		*Pleosporales*	*Acrocalymmaceae*	*Acrocalymma*
			*Aigialaceae*	*Fissuroma*, *Neoastrosphaeriella*
			*Arthopyreniaceae*	*Mycomicrothelia*
			*Astrosphaeriellaceae*	*Astrosphaeriella*, *Astrosphaeriellopsis*, *Pteridiospora*, *Pithomyces*, *Javaria*, *Triseptatospora*, *Xenoastrosphaeriella*
			*Coniothyriaceae*	*Coniothyrium*
			*Corynesporascaceae*	*Corynesporasca*
			*Delitschiaceae*	*Delitschia*
			*Dictyosporiaceae*	*Dictyocheirospora*, *Dictyopalmispora*, *Dictyosporium*, *Pseudocoleophoma*, *Sporidesmiella*
			*Didymosphaeriaceae*	*Didymosphaeria*, *Montagnula*, *Paraconiothyrium*, *Paraphaeosphaeria*, *Pseudopithomyces*
			*Hermatomycetaceae*	*Hermatomyces*
			*Lentimurisporaceae*	*Bahusandhika*
			*Leptosphaeriaceae*	*Chaetoplea*, *Leptosphaeria*, *Quasiphoma*
			*Lindgomycetaceae*	*Lolia*
*Dothideomycetes* (cont.)	*Dothideomycetidae* (cont.)	*Pleosporales* (cont.)	*Lophiostomataceae*	*Lophiostoma*, *Vaginatispora*
			*Massarinaceae*	*Haplohelminthosporium*, *Helminthosporiella*, *Helminthosporium*, *Massarina*
			*Melanommataceae*	*Asymmetricospora*, *Byssosphaeria*, *Camposporium*, *Herpotrichia*
			*Morosphaeriaceae*	*Helicascus*
			*Neophaeosphaeriaceae*	*Neophaeosphaeria*
			*Occultibambusaceae*	*Brunneofusispora*, *Neooccultibambusa*
			*Periconiaceae*	*Periconia*
			*Phaeosphaeriaceae*	*Amarenographium*, *Parastagonospora*, *Phaeosphaeria*, *Septoriella*, *Wojnowiciella*
			*Pleosporaceae*	*Bipolaris*, *Curvularia*, *Exserohilum*
			*Pseudoastrosphaeriellaceae*	*Carinispora*, *Pseudoastrosphaeriella*
			*Pseudoberkleasmiaceae*	*Pseudoberkleasmium*
			*Roussoellaceae*	*Appendispora*, *Neoroussoella*, *Roussoella*
			*Salsugineaceae*	*Salsuginea*
			*Striatiguttulaceae*	*Longicorpus*, *Striatiguttula*
			*Trematosphaeriaceae*	*Falciformispora*, *Trematosphaeria*
			*Teichosporaceae*	*Parateichospora*
			*Tetraplosphaeriaceae*	*Ernakulamia*, *Tetraploa*
			*Torulaceae*	*Cylindrotorula*, *Torula*
			*Incertae sedis*	*Acuminatispora*, *Plectophomella*, *Repetophragma*
		*Hysteriales*	*Hysteriaceae*	*Gloniopsis*
	*Incertae sedis*	*Asterinales*	*Asterinaceae*	*Asterina*, *Cirsosia*, *Discopycnothyrium*
			*Lembosiaceae*	*Lembosia*
			*Morenoinaceae*	*Morenoina*
		*Botryosphaeriales*	*Botryosphaeriaceae*	*Barriopsis*, *Botryosphaeria*, *Diplodia*, *Lasiodiplodia*, *Neodeightonia*
*Dothideomycetes* (cont.)	*Incertae sedis* (cont.)	*Botryosphaeriales* (cont.)	*Phyllostictaceae*	*Phyllosticta*
		*Jahnulales*	*Aliquandostipitaceae*	*Jahnula*
			*Manglicolaceae*	*Manglicola*
		*Kirschsteiniotheliales*	*Kirschsteiniotheliaceae*	*Kirschsteiniothelia*
			*Incertae sedis*	*Taeniolella*
		*Muyocopronales*	*Muyocopronaceae*	*Muyocopron*, *Pseudopalawania*
		*Tubeufiales*	*Tubeufiaceae*	*Aquaphila*, *Berkleasmium*, *Helicoma*, *Helicomyces*, *Helicosporium*, *Thaxteriella*
			*Wiesneriomycetaceae*	*Wiesneriomyces*
		-	*Palawaniaceae*	*Palawania*
		-	*Trichopeltinaceae*	*Acrogenotheca*
		-	-	*Letendraeopsis*, *Xenosporium*, *Brooksia*, *Dianesea*, *Leptomeliola*, *Scolionema*
*Sordariomycetes*	*Diaporthomycetidae*	*Annulatascales*	*Annulatascaceae*	*Annulatascus, Submersisphaeria*
		*Diaporthales*	*Diaporthaceae*	*Diaporthe*
			*Gnomoniaceae*	*Maculatipalma*
			*Melanconidaceae*	*Melanconis*, *Melanconium*
			*Schizoparmaceae*	*Coniella*
			*Incertae sedis*	*Durispora*, *Phruensis*
		*Distoseptisporales*	*Distoseptisporaceae*	*Distoseptispora*
		*Magnaporthales*	*Magnaporthaceae*	*Gaeumannomyces*
			*Ophioceraceae*	*Ophioceras*
			*Pseudohalonectriaceae*	*Pseudohalonectria*
		*Ophiostomatales*	*Ophiostomataceae*	*Hyalobelemnospora*
		*Phomatosporales*	*Phomatosporaceae*	*Phomatospora*
		*Tirisporellales*	*Tirisporellaceae*	*Bacusphaeria*, *Thailandiomyces*, *Tirisporella*
*Sordariomycetes* (cont.)	*Diaporthomycetidae* (cont.)	*Xenospadicoidales*	*Xenospadicoidaceae*	*Koorchaloma*, *Spadicoides*
		*Incertae sedis*	*Mesnieraceae*	*Bondiella*
			*Trichosphaeriaceae*	*Unisetosphaeria*
			*Thyridiaceae*	*Thyridium*
			-	*Paraproliferophorum*
	*Hypocreomycetidae*	*Glomerellales*	*Plectosphaerellaceae*	*Acremoniisimulans*, *Brunneomyces*
		*Hypocreales*	*Bionectriaceae*	*Acremonium*, *Clonostachys*, *Gossypinidium*, *Hydropisphaera*, *Ijuhya*, *Lasionectria*, *Nectriella*, *Paracylindrocarpon*
			*Hypocreaceae*	*Verticimonosporium*
			*Nectriaceae*	*Baipadisphaeria*, *Calonectria*, *Chaetopsina*, *Cosmospora*, *Dactylonectria*, *Fusarium*, *Ilyonectria*, *Nectria*, *Nectriopsis*, *Ophionectria*, *Pleiocarpon*, *Volutella*
			*Neoacremoniaceae*	*Neoacremonium*
			*Niessliaceae*	*Niesslia*
			*Stachybotryaceae*	*Alfaria*, *Stachybotrys*, *Virgatospora*
		*Microascales*	*Gondwanamycetaceae*	*Custingophora*
			*Halosphaeriaceae*	*Aniptodera*, *Cirrenalia*, *Fluviatispora*, *Lignincola*
			*Microascaceae*	*Wardomycopsis*
			*Triadelphiaceae*	*Triadelphia*
			*Ceratocystidaceae*	*Ceratocystis*, *Thielaviopsis*
		*Pararamichloridiales*	*Pararamichloridiaceae*	*Pararamichloridium*
	*Savoryellomycetidae*	*Conioscyphales*	*Conioscyphaceae*	*Conioscypha*
		*Pleurotheciales*	*Pleurotheciaceae*	*Monotosporella*
		*Savoryellales*	*Savoryellaceae*	*Ascotaiwania*, *Canalisporium*, *Savoryella*
	*Sordariomycetidae*	*Chaetosphaeriales*	*Chaetosphaeriaceae*	*Chaetosphaeria*, *Chloridium*, *Codinaea*, *Craspedodidymum*, *Cryptophiale*, *Dictyochaeta*, *Kionochaeta*, *Rattania*, *Sporoschisma*, *Thozetella*
*Sordariomycetes* (cont.)	*Sordariomycetidae* (cont.)	*Chaetosphaeriales* (cont.)	*Helminthosphaeriaceae*	*Endophragmiella*
			*Leptosporellaceae*	*Leptosporella*
			*Linocarpaceae*	*Linocarpon*, *Neolinocarpon*
			*Incertae sedis*	*Caudatispora*
		*Coniochaetales*	*Incertae sedis*	*Cannonia*
		*Meliolalles*	*Meliolaceae*	*Meliola*
		*Phyllachorales*	*Phaeochoraceae*	*Cocoicola*, *Phaeochora*, *Phaeochoropsis*, *Serenomyces*
			*Phyllachoraceae*	*Brobdingnagia*, *Camarotella*, *Coccodiella*, *Coccostromopsis*, *Maculatifrondes*, *Malthomyces*, *Ophiodothella*, *Oxodeora*, *Phyllachora*, *Sphaerodothis*, *Tribulatia*
		*Sordariales*	*Chaetomiaceae*	*Trichocladium*
			*Lasiosphaeriaceae*	*Cercophora*, *Lasiosphaeria*
			*Incertae sedis*	*Lockerbia*
		*Incertae sedis*	-	*Arecacicola*, *Curvatispora*, *Nigromammilla*, *Paracapsulospora*
	*Xylariomycetidae*	*Amphisphaeriales*	*Amphisphaeriaceae*	*Amphisphaeria*, *Lepteutypa*
			*Apiosporaceae*	*Arthrinium*, *Dictyoarthrinium*
			*Appendicosporaceae*	*Appendicospora*
			*Beltraniaceae*	*Beltrania*
			*Hyponectriaceae*	*Arecomyces*, *Frondicola*, *Hyponectria*, *Rachidicola*
			*Iodosphaeriaceae*	*Iodosphaeria*
			*Oxydothidaceae*	*Oxydothis*
			*Pseudomassariaceae*	*Leiosphaerella*, *Pseudomassaria*
			*Sporocadaceae*	*Bartalinia*, *Morinia*, *Neopestalotiopsis*, *Pestalotiopsis*, *Pseudopestalotiopsis*, *Robillarda*, *Seiridium*
		*Xylariales*	*Barrmaeliaceae*	*Barrmaelia*
*Sordariomycetes* (cont.)	*Xylariomycetidae* (cont.)	*Xylariales* (cont.)	*Cainiaceae*	*Arecophila, Seynesia, Endocalyx*
			*Clypeosphaeriaceae*	*Apioclypea, Brunneiapiospora, Palmaria*
			*Diatrypaceae*	*Allocryptovalsa*, *Allodiatrype*, *Anthostoma*, *Cryptovalsa*, *Diatrype*, *Diatrypella*, *Eutypa*, *Eutypella*, *Frondisphaeria*, *Peroneutypa*
			*Fasciatisporaceae*	*Fasciatispora*
			*Graphostromataceae*	*Biscogniauxia*
			*Hansfordiaceae*	*Hansfordia*
			*Hypoxylaceae*	*Annulohypoxylon*, *Hypoxylon*
			*Microdochiaceae*	*Idriella*, *Microdochium*
			*Oxydothidaceae*	*Oxydothis*
			*Robillardaceae*	*Robillarda*
			*Xylariaceae*	*Anthostomella*, *Ascotricha*, *Astrocystis*, *Diabolocovidia*, *Kretzschmaria*, *Nemania*, *Neoxylaria*, *Rosellinia*, *Stilbohypoxylon*, *Xylaria*
			*Zygosporiaceae*	*Zygosporium*
			*Incertae sedis*	*Capsulospora*, *Circinotrichum*, *Cyanopulvis*, *Gyrothrix*, *Guestia*, *Haploanthostomella*, *Lasiobertia*, *Neobarrmaelia*, *Nipicola*, *Palmicola*, *Pemphidium*, *Pulmosphaeria*, *Sabalicola*
		*Incertae sedis*	*Myelospermataceae*	*Myelosperma*
			*-*	*Frondispora, Manokwaria*
	*Incertae sedis*	*Catabotryales*	*Catabotryaceae*	*Catabotrys*
		-	*Acrodictyaceae*	*Acrodictys*
		-	-	*Apogaeumannomyces*, *Flammispora*, *Mangrovispora*

#### 3.2.4. Miscellaneous Palm Taxa

Several other *Dothideomycetes* are reported from palms and their taxonomy is either *incertae sedis* or spread over a plethora of orders and families, unlike the palmicolous *Sordariomycetes*, whose taxonomy, although highly diverse, seems to be more concentrated in some specific orders. These *Dothideomycetes* include, for example, *Acrogenotheca* (*Trichopeltinaceae*, *Dothideomycetes* family *incertae sedis*), *Bondiella* (*Mesnieraceae*, *Dothideomycetes* family *incertae sedis*), *Brooksia*, *Dianesea*, *Leptomeliola*, *Scolionema* (*Dothideomycetes* genera *incertae sedis*), *Gonatophragmium* (*Acrospermaceae*, *Acrospermales*), *Gloniopsis* (*Hysteriaceae*, *Hysteriales*), *Letendraeopsis* (*Dothideomycetes* genus *incertae sedis*), *Muyocopron*, *Pseudopalawania* (*Muyocopronaceae*, *Muyocopronales*), *Mycomicrothelia* (*Arthopyreniaceae*, *Pleosporales*), *Palawania* (*Palawaniaceae*, *Dothideomycetes* family *incertae sedis*), *Uleodothis* (*Dothideaceae*, *Dothideales*), and many other taxa occasionally reported, some of which are morphological genera only known from palms, e.g., refs. [303,536,620].

Although most palm fungi belong to the *Dothideomycetes* and *Sordariomycetes*, a series of taxa from other classes of *Ascomycota* are also often encountered on palm collections, including, for example, the genera *Mazosia* (*Roccellaceae*, *Arthoniales*, *Arthoniomycetes*), *Morchella* (*Morchellaceae*, *Pezizales*, *Pezizomycetes*), *Stictis* (*Stictidaceae*, *Ostropales*, *Lecanoromycetes*), and various *Leotiomycetes* taxa. These include members of *Helotiales*, such as, *Diplococcium* (*Vibrisseaceae*), *Hymenoscyphus* (*Helotiaceae*), *Phialocephala* (*Mollisiaceae*), *Porodiplodia* (*Porodiplodiaceae*), many species of *Lachnum* and *Lachnellula* (*Lachnaceae*), the aquatic genus *Vibrissea*, *Strossmayeria* (*Vibrisseaceae*), and some genera *incertae sedis*, viz. *Cenangiumella*, *Sorokina*, and *Sorokinella*. Fungal members of other *Leotiomycetes* orders are also represented on palms, such as *Chalara* (*Pezizellaceae*, *Rhytismatales*), *Dactylaria* (*Calloriaceae*, *Rhytismatales*), *Lophodermium* (*Rhytismataceae*, *Rhytismatales*), *Phacidium* (*Phacidiaceae*, *Phacidiales*), and *Phlyctema* (*Dermateaceae*, *Medeolariales*), e.g., refs. [240,241,622,712,713]. However, considering the comprehensive review of literature carried out here, the great diversity of palmicolous ascomycetes is basically restricted to the subphylum *Pezizomycotina*.

Very few basidiomycetes have been reported from palms and, as a result, their knowledge is practically unknown. However, some palmicolous basidiomycetes are worth mentioning due to their recurrent or specific association with palms. These include the genus *Ganoderma* (*Ganodermataceae*, *Polyporales*, *Agaricomycetes*, *Agaricomycotina*), which is an important phytopathogen that rots the lower part of palm trunks, e.g., ref. [714], and the genus *Graphiola* (*Graphiolaceae*, *Exobasidiales*, *Exobasidiomycetes*, *Ustilaginomycotina*), which parasites almost exclusively *Arecaceae* hosts, causing leaf spots on wide range of palm species, e.g., ref. [715]. Several other genera of *Basidiomycota* have occasionally been isolated from palms and studies on the oil palm basidiomycete assemblage have shown that members of *Polyporales*, such as *Fomitopsis* (*Fomitopsidaceae*), *Pycnoporus*, and *Trametes* (*Polyporaceae*), and *Agaricales* (*Agaricomycetidae*, *Agaricomycetes*, *Agaricomycotina*), such as *Schizophyllum* (*Schizophyllaceae*), may be some of the common basidiomycetes that inhabit palm substrata [523,643]. Nonetheless, more studies are needed to gain knowledge about the common basidiomycete assemblage of palm hosts.

## 4. Palm Trees as Model Plants for the Study of Fungal Biodiversity

Biodiversity is the variety of life on Earth and, consequently, depends on both taxa and their biotic and abiotic interactions [716,717]. Thus, ecological and biodiversity studies are extremely important, along with systematic and taxonomic approaches, to assess a complete and integrated perspective of the complex assemblages that make up biological communities and their ecosystems. Ecological and systematic research on palm fungi indicates that they are remarkably diverse and complex biological communities that exhibit a variety of different lifestyles. Therefore, the great diversity of palm fungi plays an important role in different aspects related to biodiversity and makes it possible to address several questions of great importance in biodiversity surveys.

Many studies have described palms as important substrata for exploring fungal diversity, particularly due to their intense fungal colonisation, e.g., refs. [7,10,12,352]. In addition, the close association and intrinsic relationship between palm hosts and palm fungi have occasionally been discussed, e.g., refs. [12,329,352,718]. Furthermore, the high fungal diversity recorded on *Arecaceae* hosts seems to be related to specific ecological and biodiversity issues observed in palm fungal communities. These include any kind of host- and tissue-specificity, or any kind of established biotic or abiotic relationships, e.g., refs. [12,13,14,329,634,718]. Here, palm trees are regarded as model plants for the study of fungal biodiversity and, therefore, the key role of palm fungi in biodiversity surveys is discussed.

### 4.1. Palm Fungi and the Search for the “Missing Fungi”

Perhaps one of the main aspects for which research on palm fungi is acknowledged is the fact that palm trees seem to harbour numerous undescribed microfungi, e.g., refs. [9,532]. To search for the undescribed mycota around the world is currently one of the main objectives of mycologists, who try to fill the gap between the number of fungal species currently described and the number of species that the most recent estimates predict. In addition, studies on fungal diversity are fundamental to increase awareness of the critical role of fungi in ecosystems. Therefore, to determine the magnitude and patterns of fungal diversity is an ongoing challenge in fungal biodiversity surveys [719,720,721].

The regular discovery of new fungal species has prompted mycologists to wonder about the number of fungi that exists worldwide. Since Fries [722], who established a comparison between the diversity of fungi and that of insects, fungi are known as one of the most speciose groups of organisms. Estimates of the number of fungal species worldwide has varied over time, ranging from relatively low numbers of 100,000 [723], 250,000 to 270,000 [724], and 712,000 [720] to impressive higher estimates of 3.5 to 5.1 [725] and almost 10 [726] and 12 million [721]. Until recently, the most widely cited and recognised number was the 1.5 million fungal species hypothesised by Hawksworth [727]. Hawksworth based his conclusions on observed ratios between fungal and plant species diversity in regions where fungi were considered to be well studied. However, even Hawksworth [727] considered this figure to be a conservative estimate. Moreover, it has been revisited several times in the literature as the worldwide description rate of new fungal species has increased over the last decades [10,728,729,730,731,732].

While molecular data is becoming the standard approach for identifying most fungal groups, environmental metabarcoding via high-throughput sequencing (HTS) is increasing the number of sequence data documenting fungal diversity worldwide [721,733,734,735,736,737,738]. Thus, predicting the number of fungal species worldwide has taken on a new dimension and divergent numbers and opinions are continuously expressed [739,740,741]. One of the ongoing debates among taxonomists is how to formally describe the so called “dark taxa”, i.e., lineages represented only by sequence data and for which no individual voucher specimens or cultures exist [589,742,743,744].

Although there is no universal approach to identifying fungi and accurately predicting fungal diversity on Earth, the currently accepted estimate of species richness is between 2.2 and 3.8 million [745]. This estimate was based on different datasets, including publication rates of new taxa, species recognition studies, extrapolations of ratios between plants and fungi, and consideration of “dark taxa” known as molecular sequence data from environmental samples [589,745]. Considering that around 150,000 accepted fungal species are currently known [746,747], these figures indicate that less than 10% of the world’s mycota have been named so far. Thus, more than 90% of all fungal species remain to be discovered. Taking into account that new species are reported at an average rate of 1500 to 2000 species per year [746,747], it could take more than 2000 years before all the missing fungi are discovered and named. More positive scenarios have been recently published, following updates to the number of named fungal species and the average rate at which fungal species are being published. Yet, the undescribed mycota will only be known in about 200 to 1800 years [746].

For all the above-mentioned reasons, the question “where are the missing fungi?” has often been asked and therefore locating and describing these taxa is a major task among mycologists [10,718,719,748,749,750]. Previous studies have highlighted that understudied biodiversity hotspots, less studied habitats and life modes, as well as less studied or fungi-rich and geographically widespread host plants (and their families), should be explored and may contain many of the missing taxa [595]. In fact, the inventory of fungal species from different substrata, especially those that seem to support a high species richness, is undoubtedly responsible for describing some of the missing fungal diversity [590].

Along with other host plants, such as bamboos (*Poaceae*) [751], *Clematis* (*Ranunculaceae*) [752], *Eucalyptus* (*Myrtaceae*) [753], *Musa* (*Musaceae*) [752], *Pandanus* (*Pandanaceae*) [754], and *Rosa* (*Rosaceae*) [755], *Arecaceae* hosts have been shown to be hyperdiverse substrata for fungal diversity, as well as a rich source of new fungal taxa. In the last three decades, a remarkable number of new taxa have been described from *Arecaceae*, e.g., refs. [6,8,9,10,17,104]). In fact, Hyde et al. [17] reported that 75% of all fungi collected on palms were new to science. In this sense, the evidence gained from the extensive palm fungi research undoubtedly indicates that many of the missing fungi can be found on palms. However, the extent of this assumption is yet to be determined and only predictions can be made.

Most of the taxa introduced from palm substrata over the last 30 years have been based solely on morphological diagnosis (Table 1). Thus, the identity of these fungi within a natural taxonomic framework has yet to be resolved. This is well illustrated when accessing *Anthostomella*-like species from palms. More than 30 species of *Anthostomella* have been introduced from palm collections, insomuch that around 60 palm species are known to be hosts of *Anthostomella* and allied genera [133,203,209,218,219,220,221,542]. However, none of the *Anthostomella* species described from palm trees have been re-evaluated in terms of their phylogenetics. Considering that recent studies have described this genus as polyphyletic and that several new taxa were hidden under *Anthostomella*-like species [539,544,545], the morphological species of *Anthostomella* described from palms may reveal a much greater diversity than initially observed. In addition, several morphological species are known to be cryptic species, i.e., distinct species that are misidentified and hidden under one species name [590,595,756]. This consequently increases the likelihood that several *Anthostomella* morphological species described from palms are unknown and misidentified taxa. Likewise, several other taxa described from palm trees have been based on morphology, which includes many of the taxa that are only known from *Arecaceae* hosts. In the last decade, many studies that used polyphasic approaches, i.e., morpho-phylogenetic analyses, have revealed numerous new taxa from widely studied regions, habitats, and hosts, e.g., ref. [757]. Recent studies have revisited the identification of palmicolous taxa and many new species are now being introduced based on morphological and sequence data, while others are being redefined according to modern DNA sequence-based classifications, e.g., refs. [522,530,531,532,533,541,607]. In turn, this has broadened and structured knowledge about the taxonomy of palm fungi (Table 2) (see Section 3). Thus, collections of palm fungi can help not only to record old taxa that need to be recollected and placed in a natural taxonomic framework, but also, undoubtedly, to reveal some of the world’s undescribed mycota.

In addition to being recognised as fungi-rich host plants, palm trees are also a geographically widespread and highly diverse group of plants. They comprise around 2600 species in 181 genera [1]. However, only a small number of palm species have been investigated for their associated microfungi. It has long been recognised that many more fungal species are reported from plants of economic importance than ones that are not recognised as of human interest [758]. This is also the case with palm trees. There is a disparity in knowledge about fungi on economically important palms compared with other palms. Thus, while economically important palm genera such as *Cocos*, *Elaeis, Phoenix*, and *Calamus* have a reported fungal richness ranging from around 650 to 1300 records, with an average value of ca. 1100 records, most palm genera have a reported fungal richness ranging from less than 10 to less than 300 records [759] (Table 3). This was well illustrated by Taylor and Hyde [8], who observed a pattern of relative species richness between different palm species—151 species for *Archontophoenix alexandrae*, 144 for *Trachycarpus fortunei*, and 77 for *Cocos nucifera*—which differed from the impression gained from species previously described as new taxa from these hosts—177 from *C. nucifera*, 12 from *A. alexandrae*, and 5 from *T. fortunei*. Given that palm trees are host plants rich in fungi, it seems obvious, looking at these figures, that there is a large amount of unstudied data on most palm species. Hence, although palm fungi have been investigated to some extent, their knowledge is still underexplored and the fungal composition for most palm species is virtually unknown.

By accessing the collection of palm fungi, especially through the reporting of taxonomic novelties, it is possible to see that more than 260 palm species in more than 95 genera have been studied for their associated microfungi [759] (Table 3). However, the majority represent occasional collections in the field, insomuch that the great majority of palm species have a reported fungal richness ranging from less than 20 to less than 50 (Table 3). However, some have been chosen for biodiversity studies because they represent palms of particular interest and/or palms that inhabit ecosystems of particular interest. Some examples are considered herein.

**Table 3:** Synopsis of fungal records on *Arecaceae* hosts retrieved from the U.S. National Fungus Collections Fungus–Host Database [759]. Palm species with less than 100 associated fungal records were disregarded from the detailed list but were considered in the summary figures.

Palm Species ^1^	Total Number of							
	Fungal Records ^2^	Fungal Species ^2,3^	Ascomycetes	Asexual Morphs	Coelomycetes	Hyphomycetes	Basidiomycetes	Zygomycetes
*Cocos nucifera*	1296	526	149 (28.33%)	275 (52.28%)	91 (17.30%)	184 (34.98%)	96 (18.25%)	6 (1.14%)
*Elaeis guineensis*	1256	427	100 (23.42%)	235 (55.04%)	50 (11.71%)	185 (43.33%)	80 (18.74%)	12 (2.81%)
*Phoenix dactylifera*	560	197	48 (24.37%)	123 (62.44%)	39 (19.80%)	84 (42.64%)	23 (11.68%)	3 (1.52%)
*Archontophoenix alexandrae*	355	178	87 (48.88%)	86 (48.31%)	11 (6.18%)	75 (42.13%)	5 (2.81%)	0
*Areca catechu*	298	155	26 (16.77%)	111 (71.61%)	33 (21.29%)	78 (50.32%)	16 (10.32%)	2 (1.29%)
*Trachycarpus fortunei*	297	154	58 (37.66%)	94 (61.04%)	41 (26.62%)	53 (34.42%)	2 (1.30%)	0
*Roystonea regia*	225	153	19 (12.41%)	123 (80.39%)	16 (10.46%)	107 (69.93%)	11 (7.19%)	0
*Livistona chinensis*	189	95	47 (49.47%)	35 (36.84%)	10 (10.53%)	25 (26.32%)	13 (13.68%)	0
*Phoenix loureiroi*	173	92	27 (29.35%)	63 (68.48%)	9 (9.78%)	54 (58.70%)	2 (2.17%)	0
*Phoenix canariensis*	160	91	24 (26.37%)	43 (47.25%)	12 (13.19%)	31 (34.07%)	24 (26.37%)	0
*Chamaerops humilis*	128	64	32 (50.00%)	22 (34.38%)	12 (18.75%)	10 (15.63%)	10 (15.63%)	0
*Sabal palmetto*	128	88	45 (51.14%)	28 (31.82%)	6 (6.82%)	22 (25.00%)	15 (17.05%)	0
*Arenga engleri*	122	64	14 (21.88%)	50 (78.13%)	4 (6.25%)	46 (71.88%)	0	0
*Licuala longicalycata*	119	89	49 (55.06%)	40 (44.94%)	3 (3.37%)	37 (41.57%)	0	0
*Rhopalostylis sapida*	113	88	36 (40.91%)	41 (46.59%)	0	41 (46.59%)	11 (12.50%)	0
**Summary figures ^1,2,3^**	Total number of palm genera from which associated fungi have been studied: 97							
	Palm genera with a total number of fungal records ≥ 100: *Cocos* (1296 fungal records), *Elaeis* (1286), *Phoenix* (1146), *Calamus* (658), *Archontophoenix* (395), *Areca* (333), *Rhopalostylis* (318), *Trachycarpus* (306), *Livistona* (278), *Sabal* (274), *Roystonea* (270), *Licuala* (244), *Arenga* (229), *Caryota* (176), *Chamaerops* (128), *Syagrus* (112), *Chamaedorea* (108), and *Borassus* (105)							
	Total number of palm species from which associated fungi have been studied: 262							
	Total number of palm species with a total number of fungal records ≥ 100: 15							
	Total number of palm species with 100 < total number of fungal records ≥ 50: 12							
	Total number of palm species with 50 < total number of fungal records ≥ 20: 26							
	Total number of palm species with a total number of fungal records < 20: 209							
	Total number of fungal records associated with *Arecaceae* hosts: 9339							
	Total number of fungal species recorded from *Arecaceae* hosts: 2932, including 1182 ascomycetes (40.31%), 332 basidiomycetes (11.32%), 1398 anamorphic fungi (47.68%), namely 984 ascomycetes (33.56%) and 413 coelomycetes (14.09%), and 20 zygomycetes (0.68%)							

^1^ All palm species names annotated in the US National Fungus Collections Fungus–Host Database were checked against the Plants of the World Online Database [760]. The fungal records that were reported from palm species identified only to genus or from unidentified *Arecaceae* hosts were only counted in the summary figures, regardless of whether their total number was more than 100. ^2^ For the total number of records and species, only the taxa of the *Fungi* kingdom were considered, so the records of *Oomycota* and *Myxomycota* associated with *Arecaceae* hosts annotated in the US National Fungus Collections Fungus–Host Database were excluded. The total number of fungal records includes records of taxa identified only to genus, as well as records of the same taxa that were obtained from different studies. ^3^ The total number of fungal species excludes taxa identified only to genus, as well as taxa that have been annotated more than once. The latter may correspond either to different collections of the same taxa on a certain *Arecaceae* host, to collections of the same taxa from different *Arecaceae* hosts, or to the same collection reported in different studies. Note: the palm species are listed in descending order of the total number of associated fungal records and species. The fungal species annotated in the US National Fungus Collections Fungus–Host Database and used to construct the table have not been verified in official nomenclatural repositories, so the current classification of some taxa is likely to be different. Taxa currently synonymised under other taxa and taxa for which “teleomorph-anamorph” connections have been established are likely to be overestimating the figures presented. This is likely to be the reason why the summary figure presented for the total number of fungal species recorded from *Arecaceae* hosts is substantially different from the global figure of palm fungi estimated in the present review (see Section 3.1).

The fungi colonising peat swamp palms have been relatively well documented, as these palms inhabit unique ecosystems comprising very distinct abiotic conditions, which are extremely important worldwide due to their rich biodiversity. The numbers of fungi and novel taxa recorded on collections of palms in the Sirindhom Peat Swamp Forest, Narathiwat, Thailand, are presented in Table 4 and Table 5, respectively.

**Table 4:** Numbers of fungi recorded on collections of palms in the Sirindhom Peat Swamp Forest, Narathiwat, Thailand.

Palm Species	Total Number of					Reference
	Fungal Records	Fungal Species	Ascomycetes	Asexual Morphs	Basidiomycetes	
*Eleiodoxa conferta*	462	112	43 (38%)	67 (60%)	2 (2%)	[248]
*Licuala longicalycata*	358	147	79 (53%)	65 (45%)	3 (3%)	[249]
*Metroxylon sagu*	82	45	21 (47%)	24 (53%)	0	[669]
*Nenga pumila*	184	47	19 (40%)	28 (60%)	0	[669]

Pinnoi et al. [248] reported a total of 462 fungal records following six field collections of *Eleiodoxa conferta*, including 112 fungal species, among which 9 new species and 1 new genus were introduced (Table 4 and Table 5). Pinruan et al. [249] reported a total of 358 fungal records following 6 field collections of *Licuala longicalycata*, including 147 fungal species, among which 9 new species and 4 new genera were introduced (Table 4 and Table 5).

**Table 5:** Novel taxa described from palm substrata collected in the Sirindhom Peat Swamp Forest, Narathiwat, Thailand.

Genus	Species	Substratum	Reference
	*Astrocystis eleiodoxae*	On a submerged petiole of *Eleiodoxa conferta*	[525]
*Baipadisphaeria*	*Baipadisphaeria spathulospora*	On a submerged trunk of *Licuala longicalycata*	[270]
	*Chalara siamensis* (as *C. siamense*)	On submerged dead petioles of *E. conferta*	[241]
	*Craspedodidymum licualae*	On a decaying trunk of *L. longicalycata*	[243]
	*Cras. microsporum*	On a decaying trunk of *L. longicalycata*	[243]
	*Cras. siamense*	On a decaying sheath of *L. longicalycata*	[243]
	*Dactylaria flammulicornuta*	On a terrestrial petiole of *Nenga pumila*	[245]
	*D. palmae*	On terrestrial sheath of *N. pumila*	[245]
	*D. uliginicola*	On a submerged rachis of *E. conferta*	[245]
*Dictyopalmispora*	*Dictyopalmispora palmae*	On decaying leaves of *L. longicalycata*	[602]
*Flammispora*	*Flammispora bioteca*	On submerged decaying leaves of *L. longicalycata*	[282]
	*Goidanichiella fusiformis* (as *G. fusiforma*)	On a submerged dead petiole of *E. conferta*	[236]
	*Jahnula appendiculata*	On a submerged trunk of *L. longicalycata*	[242]
	*Knoxdaviesia undulatistipes* (as *Custingophora undulatistipes*)	On a submerged dead petiole of *E. conferta*	[246]
*Phruensis*	*Phruensis brunneispora*	On a dead trunk of *L. longicalycata*	[301]
	*Stachybotrys palmae*	On a decaying rachis of *L. longicalycata*	[244]
	*Submersisphaeria palmae*	On submerged petioles, rachides, and trunks of *E. conferta*, *N. pumila* and *L. longicalycata*	[247]
*Thailandiomyces*	*Thailandiomyces bisetulosus*	On submerged senescent trunk of *L. longicalycata*	[307]
*Unisetosphaeria*	*Unisetosphaeria penguinoides*	On a submerged dead petiole of *E. conferta*	[245]
	*Vanakripa minutiellipsoidea*	On a submerged dead petiole of *E. conferta*	[246]

Similarly, fungi from mangrove palms, especially *Nypa fruticans*, have been widely investigated. Mangrove forests are highly specialised habitats adapted to extreme salinity conditions, which play an important role in the ecology of tropical and subtropical coastal waters. A total of 142 taxa were reported from collections of *N. fruticans*, among which 45 new species and 11 new genera were introduced [9,261,535,597,674,761] (Table 6). Although many genera and species of fungi have been well documented from *N. fruticans* and some peat swamp palms, very few molecular studies of fungi associated with these palms have been carried out (see Section 2). Thus, molecular analysis will certainly be able to provide the mycota that have not yet been discovered in the fungal communities that occur on these *Arecaceae* hosts.

**Table 6:** Novel taxa described from collections of *Nypa fruticans*.

Genus	Species ^1^	Substratum (Collection Site)	Reference
*Acuminatispora*	*Acuminatispora palmarum*	On a submerged decayed petiole (Thailand)	[261]
	*Aniptodera intermedia* *	On an intertidal petiole (Malaysia)	[166]
	*A. nypae* *	On intertidal fronds (Malaysia)	[116]
	*Anthostomella nypae* *	On an intertidal petiole (Malaysia)	[166]
	*A. nypensis* *	On an intertidal petiole (Malaysia)	[166]
	*A. nypicola* *	On an intertidal petiole (Malaysia)	[166]
	*Apioclypea nypicola* *	On an intertidal rachis (Malaysia)	[143]
	*Arecophila nypae* *	On intertidal palm tissues (Malaysia)	[131]
	*Astrocystis nypae* *	On an intertidal frond (Malaysia)	[150]
	*A. selangorensis* *	On a dead intertidal rachis (Malaysia)	[150]
	*Astrosphaeriella nipicola* (as *A. nipaecola*) (basio. *Melanopsamma nipicola*)	On palm tissues (Indonesia)	[144]
	*A. nypae* *	On decaying intertidal fronds (Brunei)	[162]
*Bacusphaeria*	*Bacusphaeria nypae* *	On petiole base (Malaysia)	[269]
*Carinispora*	*Carinispora nypae* *	On decaying intertidal fronds (Brunei)	[162]
	*Delitschia nypae* *	On a decaying fruit pericarp (Thailand)	[535]
*Fasciatispora*	*Fasciatispora nypae* *	On intertidal rotten fronds (Brunei)	[161]
*Frondicola*	*Frondicola tunitricuspis* *	On decaying fronds	[162]
	*Helicascus nypae* *	On intertidal dead fronds (Brunei)	[160]
	*Helicorhoidion nypicola* *	On intertidal palm tissues (Brunei)	[166]
	*Herpotrichia nypicola* *	On an intertidal petiole (Malaysia)	[166]
	*Leptosphaeria nypicola* *	On an intertidal petiole (Malaysia)	[166]
	*Lignincola nypae* *	On an intertidal petiole (Malaysia)	[166]
	*Linocarpon angustatum* *	On an intertidal petiole base (Malaysia)	[165]
	*L. appendiculatum* *	On rotten fronds (Brunei)	[154]
	*L. bipolare* (as *L. bipolaris)* *	On intertidal fronds (Brunei)	[105]
	*L. longisporum* *	On intertidal fronds (Brunei)	[105]
	*L. nipae* (syn. *Ophiobolus nipae*) *	On dead petioles (Philippines)	[154]
*Longicorpus*	*Longicorpus striatisporus* (syn. *Astrosphaeriella striatispora*)	On fronds (Brunei)	[9]
*Neolinocarpon*	*Neolinocarpon globosicarpum* *	On decaying intertidal fronds (Brunei)	[162]
	*N. nypicola* *	On an intertidal petiole base (Malaysia)	[165]
*Nipicola*	*Nipicola carbospora* *	On immersed fronds (Brunei)	[163]
	*N. selangorensis* *	On an intertidal frond (Malaysia)	[116]
*Nypaella*	*Nypaella frondicola* *	On intertidal fronds (Brunei)	[164]
	*Oxydothis nypae* *	On rotten fronds (Brunei)	[156]
	*O. nypicola* *	On a decayed petiole (Brunei)	[117]
	*Phomatospora nypae* *	On dead intertidal leaves (Malaysia)	[110]
	*P. nypicola* *	On an intertidal petiole (Malaysia)	[166]
	*Plectophomella nypae* *	On intertidal fronds (Brunei)	[164]
	*Pleurophomopsis nypae* *	On intertidal fronds (Brunei)	[164]
	*Savoryella nypae* (basio. *Trichocladium nypae*) *	On intertidal palm tissues (Brunei)	[166,619]
*Striatiguttula*	*Striatiguttula nypae* *	On a decayed rachis (Thailand)	[9]
*Tirisporella*	*Tirisporella beccariana* *	On decaying leaf bases (Malaysia and Phlippines)	[167]
	*Vaginatispora nypae* *	On a decaying fruit pericarp (Thailand)	[535]
	*V. palmae* *	On an immersed rachis (Thailand)	[761]
	*Vibrissea nypicola* *	On an intertidal petiole (Malaysia)	[166]

^1^ The species only known from *Nypa fruticans* are noted with a superscript asterisk (*).

*Trachycarpus fortunei* has also been relatively well studied, as it is capable of thriving in warm temperate regions and occurs naturally in the warm temperate areas of China. Thus, this makes it possible to explore the differences between fungal diversity in temperate and tropical regions. Taylor et al. [215] isolated a total of 1728 identifiable fungal endophytes from 3256 frond samples of 10 mature *T. fortunei* individuals, including 75 species in 43 genera.

Some palm species are also more studied because they are commonly found in tropical rainforests and may be geographically restricted to certain regions, i.e., endemic. Thus, they are studied as a means of expanding knowledge about tropical mycology and addressing the biodiversity of fungi that inhabit tropical hotspots. In addition, some palm species that are geographically restricted could be recognised as a valuable source of new taxa, as discussed by Taylor et al. [194] for *Archontophoenix alexandrae* in Australia. As Taylor et al. [194] stated “its endemic nature and the relative geographic isolation of its natural habitat makes it a suitable candidate for studies relating to host-specificity of fungi and fungal biogeography”. In fact, it is likely that endemic host plants that have evolved in geographic isolation can be colonised by many novel fungi when studied in their natural environment. An impressive number of more than 35 new fungal species have been introduced based on collections of *A. alexandrae*, which is certainly associated with the endemic nature of this palm tree (Table 7). A similar pattern has also been observed in other palm species. For instance, *Licuala* palms have been frequently sampled in Brunei and Australia, where they are found naturally on tropical forests. Fröhlich and Hyde [10] recorded a total of 242 taxa, including 189 species of fungi, from 2672 isolates of six *Licuala* palms. Moreover, they reported a surprising estimate of 240 fungal species occurring on 3 individual *Licuala* palms and 155 on a *Licuala* single palm in a Brunei rainforest. This investigation considered only the endophytes, saprophytes, and pathogens that develop on the leaves and petioles. Likewise, a remarkable diversity of fungal endophytes was recorded by Fröhlich et al. [11] from 6 *Licuala* palms, including 73 species in 48 genera of 2237 isolates. Similar hight species richness has also been observed in palm species endemic to temperate regions, such as the New Zealand palm *Rhopalostylis sapida* [54]. Similarly, the subendemic Cuban palm *Roystonea regia* has often been investigated in Cuba for the isolation of a huge diversity of palmicolous “anamorphs”, particularly hyphomycetes. According to Mercado-Sierra et al. [329], more than 150 species and 60 genera of fungi have been recorded from collections of *R. regia* from Cuba, among which 15 new species and 5 new genera were introduced.

**Table 7:** Novel taxa described from collections of *Archontophoenix alexandrae*.

Genus	Species	Substratum (Collection Site)	Reference
	*Aegerita queenslandica*	On a rotten leaf (Queensland, Australia)	[63]
	*Anthostomella clypeosa*	On a dead rachis (Queensland, Australia)	[8]
	*Apioclypea nonapiospora*	On a dead rachis (Hong Kong, China)	[8]
	*Astrosphaeriella immersa*	On a dead petiole (Hong Kong, China)	[148]
	*Barriopsis archontophoenicis*	On dead woody tissues (Thailand)	[549]
	*Botryosphaeria archontophoenicis*	On a dead petiole (Hong Kong, China)	[8]
	*Chaetopsina alexandrae*	On a dead rachis (Queensland, Australia)	[8]
	*Heteroconium queenslandicum*	On a rotten leaf (Queensland, Australia)	[63]
	*Hydropisphaera ciliata*	On a dead sheath (Queensland, Australia)	[8]
	*Iodosphaeria hongkongensis*	On a dead petiole (Hong Kong, China)	[146]
	*Lasiosphaeria alexandrae*	On a submerged rachis (Queensland, Australia)	[185]
	*L. alexandricola*	On a dead sheath (Hong Kong, China)	[185]
	*Linocarpon australiense* *	On palm tissues (Queensland, Australia)	[172]
	*L. luteocollum*	On a dead rachis (Queensland, Australia)	[8]
*Maculatipalma* *	*Maculatipalma fronsicola* *	On a living (Queensland, Australia)	[197]
*Manokwaria* *	*Manokwaria notabilis* *	On a dead rachis on rainforest floor (Queensland, Australia)	[109]
	*Melanographium palmicola* (as *M. palmicolum*)	On a decaying rachis (Hong Kong, China)	[182]
	*Muyocopron hongkongense*	On a dead rachis (Hong Kong, China)	[8]
	*Neolinocarpon inconspicuum* (as *N. inconspicuus*)	On a dead rachis (Queensland, Australia)	[140]
	*N. nonappendiculatum* (as *N. nonappendiculatus*)	On a dead petiole (Queensland, Australia)	[140]
	*Neoxylaria queenslandica* (as *Xylaria queenslandica*)	On a dead rachis (Queensland, Australia)	[8]
	*Oxydothis alexandrarum*	On a rotten rachis (Queensland, Australia)	[112]
	*O. australiensis*	On a rachis in forest litter (Queensland, Australia)	[112]
*Palmicola*	*Palmicola archontophoenicis*	On a basal sheath of a fallen rachis (Queensland, Australia)	[108]
	*P. bipolaris*	On a dead petiole (Queensland, Australia)	[8]
	*Phomatospora archontophoenicis*	On a dead rachis (Queensland, Australia)	[8]
	*Pseudohalonectria eubenangeensis*	On a dead rachis (Queensland, Australia)	[200]
*Pulmosphaeria*	*Pulmosphaeria archontophoenicis*	On a dead petiole (Queensland, Australia)	[194]
	*Selenosporella queenslandica*	On a rotten leaf (Queensland, Australia)	[63]
	*Sorokina frondicola*	On dead rachis (Queensland, Australia)	[8]
	*Sporidesmium queenslandicum*	On a rotten leaf (Queensland, Australia)	[63]
	*Triadelphia archontophoenicicola* (as *T. australiensis*)	On a dead rachis (Queensland, Australia)	[8]
*Tribulatia*	*Tribulatia appendicospora*	On a dead petiole (Queensland, Australia)	[8]
	*Trichoconis queenslandica*	On a rotten leaf (Queensland, Australia)	[63]
	*Volutella queenslandica*	On a rotten leaf (Queensland, Australia)	[63]

* New taxa whose designated holotype material corresponds to collections of other palm species or unidentified palms and not *Archontophoenix alexandrae*, but which were also isolated from collection of *A. alexandrae* when introduced as new to science.

Several studies have also investigated the fungal communities on tissues of selected palms in tropical and subtropical regions, e.g., refs. [13,14], as well as in tropical and temperate regions, e.g., ref. [8]. Yanna et al. [13] identified 288 different taxa from fungal communities on decaying fronds of *Livistona australis*, *Oraniopsis appendiculata* (Australia), *Arenga engleri*, *L. chinensis* (Hong Kong), *A. undulatifolia*, *Salacca affinis*, and *Oncosperma horridum* (Brunei), among which 17 undescribed species were found. Similarly, Taylor and Hyde [8] studied the microfungi associated with three palm species in areas where they were native and where they had been introduced. They identified a total of 288 different taxa, including one new genus and 34 undescribed species, 26 of which showed host-specificity at species level.

All these figures demonstrate the extraordinary richness of palms for research into fungal biodiversity. The high number of fungal taxa found confirms that the fungi on palms are diverse and can be a source of many undescribed species. Furthermore, many palm species inhabit some of the world’s biodiversity hotspots, including areas of South America and India, where many palm species are native or even endemic. Although considered biodiversity-rich areas, both South America and India are poorly explored regions in terms of fungal diversity. Some studies have reported a high diversity of palm fungi from India and Brazil, e.g., refs. [437,501]. However, no comprehensive study has yet been carried out on the composition of palm fungi in these regions (see Section 2). Since a large number of fungi inhabit biodiversity hotspots, it is to be expected that many unknown fugal taxa inhabit palm trees native to these regions, especially considering the lack of studies in this regard.

Collecting fungi based on a chosen host is one of the most popular methods for studying fungal diversity. In addition to being fungi-rich and geographically widespread hosts, palms are mostly distributed in the tropical and subtropical regions of the world, some of which are underexplored biodiversity hotspots [590,595]. Thus, palms have several characteristics that increase the possibility of discovering new fungal species, which makes them ideal hosts for searching part of the worldwide unknown mycota. Therefore, palm trees should be prioritised for seeking new taxa and studying fungal diversity, given the ecological possibilities they can represent and reflect in the composition of their fungal assemblages. A number of studies have documented the role of palm trees and the corresponding palm fungal communities in biodiversity surveys. A notable example is the investigation carried out by Hyde and co-workers, which allowed estimates of fungal biodiversity to be questioned and adjusted to more reasonable values.

### 4.2. Palm Fungi and the Fungal Biodiversity Estimates

The studies carried out by Hyde and co-workers resulted in a wealth of data that provided new information for estimating fungal biodiversity. Much of these data were discussed and revised by Fröhlich and Hyde [10], who wondered whether the estimates of global fungal biodiversity at the time were realistic after the remarkable diversity observed in the fungal communities of palm trees in the tropics. For this reason, they considered that the estimate of 1.5 million species proposed by Hawksworth was a “very conservative estimate of the number of fungal species extant on the planet”. Furthermore, fungus to plant ratios seem to be noticeably higher on palms compared with those estimated by Hawksworth [727,728], when revisiting the numbers of fungal diversity on Earth. Thus, determining fungus to plant ratios on palms and, consequently, its contribution to estimates of fungal numbers is of great importance in fungal biodiversity surveys.

Hyde [762,763], reviewing his extensive work on palm fungi in North Queensland, estimated that there are about 3 pathogens, 10 saprophytes, and 100 endophytes that can develop on each palm species. In addition, Hyde [762,763] considered that 25% of these fungi are likely to be host-specific, i.e., restricted to a single host species (compared with the 67% host-specificity assumed by Hawksworth [727]). As a consequence, about 28 fungal taxa are likely to be associated with each palm species. This astounding plant to fungus ratio of 1:28 would imply the existence of almost 73,000 species of fungi on palms worldwide, of which only less than 3% (ca. 1580 species) were known [8]. However, following detailed investigation on six palm trees in Australia and Brunei carried out by Fröhlich and Hyde [10], the 1:28 ratio was subsequently revised upwards to 1:33. As Hyde [762,763] stated, after years of experience with palm fungi, it appears that “with palms the host species to fungi ratio is much higher”. Therefore, values ranging from 1:28 to 1:33 would be a more accurate estimate than the much lower and conservative plant:fungus ratios that have been estimated over the years [10,762].

The figures for plant:fungus ratios on palms are of marked significance for the total numbers of fungi worldwide. Plant:fungus ratios rely heavily on the concept of whether fungi are host- and/or tissue/organ-specific or have host- and/or tissue/organ-recurrence. Thus, host-specificity or -recurrence is probably the most important single factor used in estimating global fungal numbers [728,764]. The concepts of host-specificity and host-recurrence are not distinguished in the context of this review and are often used synonymously. However, host-specificity may be an inappropriate term for saprobic fungi. Zhou and Hyde [764] suggested host-exclusivity and host-recurrence as more appropriate terms (for a definition and discussion of these concepts, see Zhou and Hyde [764]).

There is now much circumstantial evidence that many palm fungi are host- and/or tissue-specific, and their impact on palm fungi numbers are discussed here. Many palm fungi have only been recorded on *Arecaceae* or sometimes on other large woody monocotyledons, such as *Pandanaceae* hosts. This could be due to similarities in the physical nature of the substratum of these plant families, which produce relatively large, thick leaves, which offer a range of microhabitats for fungal growth. Moreover, palm fungi differ widely from the taxa recorded on other monocotyledons, such as grasses.

#### 4.2.1. Fungal Specificity at Family, Genus, and Species Levels

Host-specificity infers a relationship between hosts and fungi and has mostly been applied to plant pathogens. There are numerous examples of host- and tissue-specific plant pathogens [765,766,767,768]. However, most fungi on palms are not pathogens, and therefore are unlikely to be host-specific. They may, however, exhibit a host-recurrence, i.e., occur repeatedly on the same host, but be absent or rare on adjacent hosts of the same family [764].

An extremely high diversity of palm saprophytes was found developing on a wide range of dead palm material. Saprobic fungi are less likely to be host-specific [764]. However, in the great diversity of saprophytic fungi supported by palm tissues, many species are found exclusively or recurrently on palms. Therefore, it would be expected that some saprophytic fungi be selective to specific palm species or genera. This was found to be true and is well documented on mangrove palm trees, e.g., refs. [7,165]. At which level this specificity occurs, i.e., host genus, subtribe, tribe, or subfamily, is not yet obvious, but should become clear as the mycota of more palm hosts are systematically investigated. In fact, following his studies on the fungi on palms in North Queensland, Australia, Hyde [769] listed several species and genera of fungi that are thought to be unique to *Archontophoenix alexandrae* and other palm genera, as they have not been identified in detailed studies of other hosts occupying the same habitat. As Hyde [769] stated “these fungi are almost certainly genus-specific and some may also be host-specific” (Table 8).

**Table 8:** Possible host-specific fungi known from a single palm species or genus in Australia (adapted and updated from [769]).

Palm Species/Genus	Fungal Species	Reference
*Archontophoenix alexandrae*	*Hydropisphaera ciliata*	[8]
	*Lasiosphaeria alexandrae*	[185]
	*Lockerbia palmicola* *	[114]
	*Neolinocarpon inconspicuum*	[140]
	*N. nonappendiculatum*	[140]
	*Oxydothis alexandrarum*	[112]
	*O. australiensis*	[112]
	*Palmicola archontophoenicis*	[194]
	*P. bipolaris*	[8]
	*Phomatospora archontophoenicis*	[8]
	*Pseudohalonectria eubenangeensis*	[200]
	*Pulmosphaeria archontophoenicis*	[194]
*Calamus*	*Anthostomella bipileatus*	[6]
	*Astrosphaeriella australiensis*	[144]
	*Cyanopulvis australiensis*	[6]
	*Neolinocarpon australiense*	[140]
	*Oxydothis calami*	[117]
	*O. luteaspora*	[112]
	*O. rubella*	[112]
	*O. uniseriata*	[6]
	*Pemphidium calamicola*	[135]
	*P. rattanicola*	[6]
	*Pseudohalonectria palmicola*	[200]
	*Roussoella calamicola*	[147]
*Cocos nucifera*	*Mycosphaerella palmicola*	[198]
*Licuala*	*Ascotaiwania licualae*	[6]
	*Capsulospora angustispora*	[6]
	*Nectriella erythroclypea*	[121]
	*Nipicola licualae*	[6]
	*Oxydothis angustispora*	[6]
	*O. cyrtospora*	[6]
	*O. extensa*	[6]
	*O. parasitica*	[195]
*Linospadix*	*Oxydothis linospadicis*	[195]
	*O. obducens*	[117]
*Oraniopsis appendiculata*	*Monotosporella palmicola*	[15]
	*M. sphaerica*	[15]
	*Palmaria montanea*	[143]
	*Sporidesmiella oraniopsidis*	[230]
*Pinanga* sp.	*Phyllosticta candeloflamma*	[187]

* Although *Lockerbia palmicola* has been included in the present list as a possible host-specific fungal species of *Archontophoenix alexandrae*, the species was introduced by [114] on dead palm rachides on forest floors from North Queensland, Australia, which were referred to as “possibly *Archontophoenix*”. Thus, this fungal species may be specific to other *Archontophoenix* species, rather than *A. alexandrae*, or even to the genus *Archontophoenix*. Note: only fungal species known from more than one collection were included.

Although most palmicolous taxa will not be specific to a particular palm species, most of them belong to genera that specialise on palm hosts [17]. Many fungi that are saprobes on palms appear to be unique or occur disproportionately on palms relative to other hosts. Many genera typically found on palms, such as *Arecomyces*, *Arecophila*, *Ascotaiwania*, *Manokwaria*, *Myelosperma*, *Neolinocarpon*, *Palmicola*, and *Pemphidium*, comprise species known only from palms, while many speciose genera, such as *Astrosphaeriella*, *Linocarpon*, and *Oxydothis*, are predominantly found on palms [17,718,764]. Some of these genera were originally described from palm substrata (Table 1) and remain taxa that are apparently restricted to palms. In some cases, specificity seems to have a wider taxonomic range. For instance, members of the *Phaeochoraceae* (*Phyllachorales*), such as *Cocoicola*, *Phaeochora*, *Phaeochoropsis*, and *Serenomyces*, are saprotrophic or biotrophic ascomycetes on plant leaves that are apparently restricted to the *Arecaceae* [212]. Some examples of host-specificity suggested in palmicolous taxa are presented below, along with comments on the factors that can justify their specificity.

Although several *Oxydothis* species are known from more than one palm host, some are only known from a single palm host. This has been observed with *O. alexandrae*, which was frequently collected on decaying petioles of *A. alexandrae* but was not found on adjacent host palms, including *Calamus* or *Licuala* palms inhabiting the same habitat or region [12,117]. Thus, this taxon is a good example of an apparently saprobic fungus showing host-specificity. Hyde et al. [180] observed that species of *Oxydothis* and *Cocoicola* develop on the fronds before they completely dry out, suggesting that they may be endophytes that convert to a saprobic lifestyle with the onset of senescence. A similar situation occurs with *Neolinocarpon nypicola* on *Nypa fruticans*. The blackened stroma of this fungus forms throughout the senescing palm material, long before it has the appearance of being rotten [165]. In addition, it has recently been shown that saprobic *Oxydothis* species inhabiting dead palm tissues can produce appressoria by germinating ascospores [528]. Appressoria are specialised infection structures used by pathogenic taxa to infect their hosts and have rarely been observed in saprobic fungi, e.g., ref. [770]. The production of appressoria by saprobic taxa suggests that they may be adapted to an endophytic lifestyle and become active after host senescence [718,771]. The appressoria found on germinating ascospores of *Oxydothis* species from palms suggest that these taxa can infect healthy plants as endophytes, making them the first colonisers of dead palm material as saprobes [211].

Species of *Astrosphaeriella* are more commonly associated with the climbing or rattan palms, such as *Calamus*, *Daemonorops*, and *Livistona*, which indicate some degree of host-specificity [12]. For example, *A. bakeriana* is one of the first and most common taxa to appear on dead fronds and leaves of *Livistona chinensis* in Hong Kong and is hardly known from any other host, although it seems to be common throughout the Asian region [144]. Guo et al. [639], while studying the endophytes of *L. chinensis*, showed that this common saprobe is also an endophyte at an earlier stage. Many other fungi that were isolated as endophytes from palms have been shown to occur as common saprobes [639]. If many of the saprobes develop from endophytes, then it is likely that many saprobic fungi on palms are host-specific. As Guo et al. [639] pointed out “some endophytes and saprotrophs are interrelated, i.e., some saprotrophs have a latent period inside plant tissues, or some endophytes become saprotrophs after plants scenesce”. It is imperative to understand the mechanism of life mode conversion in fungi, as such conversions would have a significant impact on fungal diversity. The extraordinarily rich mycota found on palm trees in the tropics is likely to reveal new insights into this suggested change in life mode between endophytes and saprobes, or even between endophytes and pathogens. This fungus–host relationship, in which the plant tolerates the fungus in its tissues as an endophyte, is likely to have evolved over a long period, so it is likely to have resulted in saprobes having a host preference [763]. Thus, given that many fungi are specific or recurrent on palms, this may be one of the reasons why so many undescribed taxa are found on *Arecaceae* hosts. As Zhou and Hyde [764] stated, common fungal genera on palms may be endophytes that become saprobes on senescent plant parts, which would justify such high plant:fungus ratios in these hosts.

Most *Neodeightonia* species are exclusively or almost exclusively found on arecaceous hosts, and the co-evolution of *Neodeightonia* species as endophytes with these hosts to adapt to new environmental conditions has been recently discussed [547]. For example, *N. phoenicum* has only been reported from *Phoenix* spp. to date, so it is apparently restricted to palms and may represent an example of host-specificity at the genus level, e.g., ref. [548].

Many examples of host-specific fungi have also been suggested in palmicolous hyphomycetes. *Brachysporiella* species are mainly found on palms, although they also grow in other habitats [103]. Other genera, such as *Ceratosporella*, *Endocalix*, *Piricauda*, *Phragmospathula*, and *Phragmospathulella*, are practically exclusive to palms. In some cases, such as *Holubovaea* and *Consetiella*, specificity even appears at the level of host genus or host species, in this case *Roystonea regia* [329,352]. However, in cosmopolitan or speciose genera of hyphomycetes, this specificity is often lost. Speciose genera are more heterogeneous and, in turn, their species are more genetically diverse. Greater genetic diversity leads to greater adaptability to the environment, so they can be found in palm trees and other substrata [329]. An interesting case of host-specificity is the species *Holubovaea roystoneicola* described on petioles of *R. regia* from Cuba [73]. There is no record of this species on a different host plant since its introduction 40 years ago, nor in any other region outside Cuba. However, it has been collected more than 140 times in different Cuban localities and always inhabiting *R. regia* [352].

One remarkable study that gave new insights into the issue of host-specificity was that of Fröhlich and Hyde [10]. They compared the fungal communities on three *Licuala ramsayi* palms in Northern Queensland, Australia, and on a different unidentified species of *Licuala* in a pristine tropical rainforest in Brunei Darussalam. Only 30 of 242 taxa overlapped between the fungal communities recorded on both *Licuala* species, although some of these taxa may have been misidentified, as it was not possible to resolve them using molecular data. As Hyde et al. [756] asked, if fungal species were not mainly host- or genus-specific, how did almost completely different communities occur on these palm species of the same genus but in different countries?

Studies on the fungal communities of different terrestrial palm species have suggested that both host genera and host species affect the composition of these communities in relation to the fungal species recovered from palm tissues (Table 9). Yanna et al. [13] studied the fungal composition of communities recovered from decaying fronds of seven palm species in Australia, Brunei, and Hong Kong and reported that few fungi were common to palms of the same genus and the number was lower on palms of different genera. In Brunei and Hong Kong, only 10% and 17%, respectively, of the fungi recorded were common to palms of different genera, while in Australia the numbers were even lower, ranging from 6 to 9%. Furthermore, only 5 to 23% of the fungi were common to two of the palms studied. These figures strongly suggest evidence for host-specificity on different palm host genera. Similarly, Yanna et al. [634] and Taylor and Hyde [8] studied fungal communities on *Phoenix hanceana* and *A. alexandrae* in Hong Kong, respectively, and fungi overlapping with those on other palm hosts in Hong Kong was very low.

**Table 9:** Ten most common species recorded on selected terrestrial palm species in ecological studies conducted in different countries (adapted from [16]).

*Arenga engleri* (Hong Kong)	*Arenga undulatifolia* (Brunei)	*Calamus* sp. (Thailand)	*Livistona chinensis* (Hong Kong)	*Oncosperma horridum* (Brunei)	*Phoenix hanceana* (Hong Kong)	*Salacca affinis* (Brunei)
*Piricauda cochinensis*	*Piricauda cochinensis*	*Tetraploa* sp.	*Astrosphaeriella bakeriana*	*Linocarpon livistonae*	*Diplococcium stoveri*	*Zygosporium minus*
*Diplococcium stoveri*	*Melanographium selemiodes*	*Morenoina palmicola*	*Lachnum palmae*	*Craspedodydimum nigroseptatum*	*Endocalyx cinctus*	*Linocarpon livistinae*
*Helminthosporium solani*	*Trichoderma harzianum*	*Circinoconis paradoxa*	*Appendicospora hongkongensis*	*Zygosporium minus*	*Cryptophiale udagawae*	*Peltistromella anomala*
*Melanographium palmicola*	*Zygosporium minus*	*Diaporthe* sp.	*Monodictys putredinis*	*Monotosporella setosa var. macrospora*	*Penzigomyces nodipes*	*Helicosporium griseum*
*Melanographium selenioides*	*Pleurophragmium* sp.	*Helminthosporium* sp.	*Oxydothis elaeicola*	*Neolinocarpon australiense*	*Thozetella effusa*	*Volutella ciliata*
*Monodictys putredinis*	*Helmithosporium velutimum*	*Linocarpon* sp.	*Trichoderma harzianum*	*Trichoderma harzianum*	*Pseudospiropes simplex*	*Oxydothis luteaspora*
*Oxydothis ragai*	*Volutella ciliata*	*Phaeosphaeria* sp.	*Neolinocarpon australiense*	*Oxydothis luteaspora*	*Dictyochaeta simplex*	*Periconiella* sp.
*Pestalotiopsis palmarum*	*Peltistromella anomala*	*Anthostomella* sp.	*Fasciatispora petrakii*	*Oxydothis licualae*	*Serenomyces shearii*	*Arecomyces bruneiensis*
*Guignardia manokwaria*	*Stachylidium* sp.	*Astrosphaeriella* sp. 1	*Corynesporopsis isabelicae*	*Oxydothis elaeicola*	*Capsulospora brunneispora*	*Sporidesmium parvum*
*Dischoridium roseum*	*Anthostomella minutoides*	*Goidanichiella fusiformis*	*Dictyosporium elegans*	*Brachysporiella gayana*	*Harknessia globosa*	*Codinaea intermedia*

Note: the taxa are listed in descending order of their percentage abundance. The taxa listed are only those identified at least to genus level; unidentified taxa have been disregarded. The taxa names are presented according to the literature used to construct the table, regardless of whether their current classification is different.

**Figure 2:**
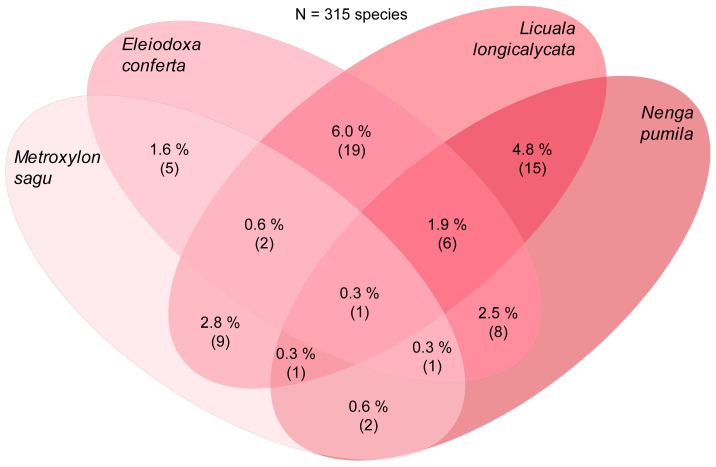
Percentage of fungal overlapping between the four peat swamp palm species in ecological studies conducted in the Sirindhom Peat Swamp Forest, Narathiwat, Thailand (adapted from [669]).

Host-specificity has also been strongly suggested on the fungal communities of palms inhabiting peat swamp and mangrove forests. Pinnoi et al. [248] and Pinruan et al. [249] documented the fungal communities that occur on the peat swamp palms *Eleiodoxa conferta* and *Licuala longicalycata*, respectively. They observed a very low overlap between the fungi and the dominant mycota found on terrestrial palms. While genera such as *Anthostomella*, *Arecomyces*, *Linocarpon*, *Oxydothis*, and *Sorokinella* are generally common on terrestrial palms, but do not usually constitute a dominant group on peat swamp palms, showing a marked difference in the percentage of occurrence, species of *Astrosphaeriella* tend to be common to both terrestrial and peat swamp palms. Recently, these data were revisited and analysed by Pinruan et al. [669], who also documented the diversity of fungi occurring on two other peat swamp palms, *Metroxylon sagu* and *Nenga pumila*. These studies report either some taxa common on submerged wood (e.g., *Brocchiosphaera brocchiata*, *Dictyochaeta gyrosetula* and *Thozetella nivea*) and many taxa common to those known to come from terrestrial palms (e.g., *Astrosphaeriella* spp., *Linocarpon* spp., *Massarina* and *Oxydothis*). However, the percentage of overlap between these communities is low and most of the taxa found are exclusive to peat swamp palms and have never been documented in other habitats. When analysing the percentage of overlap in fungal biodiversity between the four peat swamp palms, Pinruan et al. [669] reported that of the ten most common fungal species, or even genera, occurring on each of them, no taxa was found to be common to the four palms (Table 10). The results reinforce that each of the four peat swamp palms supports its own specific fungal community. Moreover, the overlap of fungal species between the four palms was less than 1%, while between three and two it was less than 2% and less or 6%, respectively, even though they all grew in close proximity in a peat swamp forest in Thailand (Figure 2).

**Table 10:** Ten most common genera and species recorded on each and all of the four peat swamp palm species in ecological studies conducted in the Sirindhom Peat Swamp Forest, Narathiwat, Thailand (adapted from [669]).

Taxonomic Rank	*Eleiodoxa conferta*	*Licuala longicalycata*	*Metroxylon sagu*	*Nenga pumila*	Peat Swamp Palms
Genera	*Astrosphaeriella*	*Astrosphaeriella*	*Nawawia*	*Diplococcium*	*Astrosphaeriella*
	*Stilbohypoxylon*	*Oxydothis*	*Anthostomella*	*Dinemasporium*	*Microthyrium*
	*Cancellidium*	*Annulatascus*	*Oxydothis*	*Linocarpon*	*Stilbohypoxylon*
	*Xylomyces*	*Massarina*	*Apiospora*	*Arecomyces*	*Cancellidium*
	*Lophiostoma*	*Microthyrium*	*Cylindrocladium*	*Spadicoides*	*Diplococcium*
	*Microthyrium*	*Phaeoisaria*	*Dinemasporium*	*Lophodermium*	*Oxydothis*
	*Morenoina*	*Nectria*	*Tetraploa*	*Sporidesmium*	*Xylomyces*
	*Phaeoisaria*	*Phruensis*	*Apioclypea*	*Dactylaria*	*Lophiostoma*
	*Jahnula*	*Submersisphaeria*	*Ornatispora*	*Oxydothis*	*Phaeoisaria*
	*Annulatascus*	*Thozetella*	*Massarina*	*Jahnula*	*Annulatascus*
Species	*Cancellidium applanatum*	*Microthyrium* sp.	*Anthostomella bipapillispora*	*Diplococcium stoveri*	*Microthyrium* sp.
	*Xylomyces aquaticus*	*Phaeoisaria clematidis*	*Nawawia filiformis*	*Dinemasporium* sp.	* Cancellidium applanatum *
	*Astrosphaeriella aquatica*-like	*Annulatascus velatispora*	*Oxydothis*-like	*Arecomyces epigeni*	* Diplococcium stoveri *
	*Stilbohypoxylon elaeicola*	*Massarina bipolaris*	*Apioclypea eccentricospora*	*Linocarpon* sp. 4	* Xylomyces aquaticus *
	*Lophiostoma frondisubmersa*	*Phruensis brunneispora*	*Apiospora* sp.	*Lophodermium* sp.	* Phaeoisaria clematidis *
	*Microthyrium* sp.	*Solheimia costaspora*	*Dinemasporium lanatum*	*Dactylaria palmae*	* Astrosphaeriella aquatica * -like
	*Morenoina palmicola*	*Thailandiomyces bisetulosus*	*Tetraploa aristata*	*Lophiostoma* sp.	* Stilbohypoxylon elaeicola *
	*Phaeoisaria clematidis*	*Nectria* sp. 1	*Ornatispora* sp.	*Oxydothis* sp. 8	* Jahnula appendiculata *
	*Stilbohypoxylon eleiodoxae*	*Helicoma* sp. 1	*Massarina bipolaris*	*Spadicoides* sp. 4	* Lophiostoma frondisubmersa *
	*Jahnula appendiculata*	*Astrosphaeriella malayensis*	*Acrogenospora sphaerocephala*	*Jahnula appendiculata*	* Morenoina palmicola *

Note: the taxa are listed in descending order of their percentage abundance. The taxa names are presented according to the literature used to construct the table, regardless of whether their current classification is different.

A very low overlap in fungal diversity is similarly observed between peat swamp palms and the mangrove palm *N. fruticans*. None of the 10 most common fungal species on each peat swamp palm studied has been recorded on *N. fruticans*, even though its fungal diversity has been well documented in Brunei, e.g., refs. [154,162], Malaysia, e.g., refs. [110,165], the Philippines, e.g., refs. [673,772], Thailand, e.g., refs. [9,162,261], and Indonesia, e.g., ref. [117] (Table 11). Likewise, few of the fungi recorded on *N. fruticans* have been recorded inhabiting peat swamp palms [674]. When compared with the peat swamp palm *E. conferta*, only a few species are common to both palms in *Astrosphaeriella*, *Linocarpon*, and *Oxydothis*. However, the genera *Carinispora*, *Fasciatispora*, *Halocyphina*, *Helicascus*, *Lignincola*, and *Lulworthia*, which are common on *N. fruticans*, have not been recorded on *E. conferta*, as these genera are more commonly found on substrata in marine habitats and may require salt for growth, while those on *E. conferta* may not be salt tolerant. The latter may be more tolerant to acidic waters, while marine fungi tend to occur in more alkaline waters [248,674]. A similar pattern of low overlap in fungal composition is observed between the peat swamp palm *L. longicalycata* and *N. fruticans*, although some taxa are common to both palms, including species of *Helicoma*, *Helicosporium* and *Thozetella* [249,674]. Thus, most of the fungi found on Nipa palm is intertidal and do not appear to occur on other palms. In addition, species composition on this palm also differs from that on mangrove wood [168,671].

*Nypa fruticans* grows in the brackish waters of the intertidal region along marine coastlines in the tropics and extends into freshwater zones. This highly specialised habitat makes *N. fruticans* an interesting host for fungal colonisation. Fungi occurring on *N. fruticans* can be categorised into three main groups: typically marine/mangrove fungi (e.g., *Aniptodera chesapeakensis*, *Halocyphina villosa*, *Kallichroma tethys*, *Marinosphaera mangrovei*, *Lignincola laevis*, *Lulworthia* spp., *Savoryella paucispora*, *Saagaromyces ratnagiriensis*, *Sammeyersia grandispora* and *Verruculina enalia*), many of which appear to be host-specific (e.g., *Aniptodera nypae*, *Fasciatispora nypae*, *Helicascus nypae*, *Helicorhoidion nypicola*, *Lignincola nypae*, *Savoryella nypae*, and *Tirisporella beccariana*); typically freshwater fungi (e.g., *Anthostomella eructans*, *Annulatascus velatisporus*, *Helicoma hongkongense*, *H. hyalonemum*, *H. pannosum*, and *Thozetella nivea*); and fungi from typical palm-inhabiting fungal genera, most of which also appear to be exclusive to this palm (e.g., *Anthostomella nypae*, *Linocarpon angustatum*, *L. appendiculatum*, *L. bipolare*, *L. nipae*, *Oxydothis nypae*, and *O. nypicola*) [671,672,673]. To-date, 142 fungi have been documented growing on *N. fruticans*, of which 42 are only known from this host [9,261,535,597,674,761] (Table 6). Thus, one can assume ca. 30% of host-specificity for the fungal species recorded on *N. fruticans*. In fact, *N. fruticans* appears to support a large number of unique fungi, which do not overlap with those occurring on terrestrial palms [13].

**Table 11:** Ten most common species recorded on *Nypa fruticans* in different ecological studies conducted in different countries.

Thailand ^1^	Brunei ^2^	Philippines ^3^
*Trichocladium nypae*	*Linocarpon bipolare*	*Linocarpon appendiculatum*
*Linocarpon appendiculatum*	*Linocarpon appendiculatum*	*Microthyrium* sp.
*Lulworthia grandispora*	*Oxydothis nypae*	*Astrosphaeriella striatispora*
*Oxydothis nypae*	*Astrosphaeriella striatispora*	*Oxydothis nypicola*
*Astrosphaeriella striatispora*	*Trichocladium nypae*	*Halocyphina villosa*
*Helicorhoidion nypicola*	*Lignincola nypae*	*Didymella* sp.
*Aniptodera nypae*	*Neolinocarpon globosicarpum*	*Lignincola nypae*
*Lignincola laevis*	*Sporidesmium crassisporum*	*Helicorhoidion nypicola*
*Dictyosporium elegans*	*Helicorhoidion nypicola*	*Aniptodera intermedia*
*Anthostomella* cf. *rehmii*	* Aniptodera nypae *	*Massarina* sp.

^1^ Data from [671]. ^2^ Data from [672]. ^3^ Data from [673]. Note: the taxa are listed in descending order of their percentage abundance. The taxa names are presented according to the literature used to construct the table, regardless of whether their current classification is different.

It should be noted that most of the palms studied were only one species of a given genus, so it is unclear whether the data obtained is the result of host- or genus-specificity. Some cases of host-specificity seem to be well established, such as that observed in fungal communities of *N. fruticans*, which is the only species in the *Nypa* genus. There are other intertidal mangrove palms, such as *Calamus erinaceus*, *Oncosperma tigillarium*, and *Phoenix paludosa*. It is therefore necessary to examine the fungi on these hosts to determine whether there is overlap in fungal communities. This will certainly fine-tune the percentage of host-specificity predicted for *N. fruticans*. However, *N. fruticans* is notable for the fact that more than 40 fungal species are exclusive to this host. Thus, although it is unclear whether the same fungi also occur on the other three intertidal palms, even if they did, the ratio of these palm hosts to specific intertidal fungi is extremely high.

#### 4.2.2. Fungal Specificity at Organ/Tissue Level

In addition to host-specificity at the plant family, genus, and species levels, studies on palm fungi have also revealed host-specificity at the organ/tissue level. Palm trees comprise several different types of tissue with different textures and chemistries, including roots, trunks, petioles, rachides, leaves, and flowers. For instance, species of *Astrosphaeriella* are particularly common on the aerial stems or trunks of climbing or rattan palms, which suggests some degree of tissue-specificity [12]. In fact, different palm tissues have been found to support different assemblages of saprobic and endophytic fungi [17], and this difference is probably due to substratum structure. For instance, Hyde et al. [17] reviewed the ascomycetes reported on palms and compiled a list of some common genera reported from different palms structures. These include species of *Anthostomella*, *Lembosia*, *Meliola*, *Mycosphaerella*, *Phyllachora*, and *Sphaerodothis* predominantly found on leaves, and species of *Anthostomella*, *Astrosphaeriella*, *Linocarpon*, *Oxydothis*, *Rosellinia*, and *Xylaria* predominantly found on rachides. Likewise, Hyde et al. [17] reported that the few taxa described from palm inflorescences were not found in other tissues. Thus, fungi associated with the reproductive tissues of palms are likely to form a distinct assemblage of species when compared with other palm fungal communities [10]. This was found to be true in several palm fungal communities recovered from different palm tissues.

Tissue-specificity has been suggested in endophytic palm fungal communities. A higher isolation rate, i.e., the recovery of a greater number of isolates, is often observed in vein rather than intervein tissues in both tropical [11,291,469] and temperate [215] palmicolous endophytes. Moreover, differences are often observed between the endophytic mycotas of different palm tissues and the tissues of different ages [11,215]. Tissue-specificity has also been suggested by the preference of xylariaceous taxa for leaf tissues [11,215] and of coelomycetes taxa for petioles [11].

Hyde and Alias [7] found that different fungi colonised different parts of the fronds of *Nypa fruticans*, including the leaves, leaf midribs, petioles, and petiole bases, indicating that some fungi may develop preferentially on certain types of tissue. Similar results were found on terrestrial palms. Yanna et al. [13] investigated the effect of different parts of the decaying fronds of seven different palm species on fungal communities. They reported that distinct fungal communities occurred on the leaves and rachis-tips, mid-rachides, and rachis-bases of most of the palm species examined. The exception was *Livistona australis* in which the fungal communities occurring on different parts of the rachides were more similar, probably due to their similar structures. However, the fungi on the leaves were distinct. Yanna et al. [14] investigated the effect of different parts of the decaying fronds of *L. chinensis* from Hong Kong on fungal communities. Distinct fungal communities were also observed, insomuch that 25 to 70% of the fungi recorded during different periods of decay were restricted on either leaves or petioles, which included 20 and 54 of 91 species confined to leaves and to petioles, respectively. For example, *Appendicospora hongkongensis* and *Cocoicola livistonicola* have only been recorded on petioles, while *Pseudospiropes arecacensis* and *Vesiculozygosporium echinosporum* (syn. *Zygosporium echinosporum*) have only been recorded on leaves [14]. Pinnoi et al. [16] also found that the petioles of *Calamus* spp. supported a greater species diversity than rachides (61% versus 39%, respectively) and while many species, such as *Melanographium citri*, *Astrosphaeriella vesuvius*, and *Berkleasmium micronesiacum* (syn. *Coleodictyospora micronesiaca*), were confined to petioles, only *Lachnellula* sp. occurred exclusively on rachides. Likewise, fungi were found to be more abundant and diverse on the petioles of the peat swamp palms *Eleiodoxa conferta* [248] and *Licuala longicalycata* [249] than on their trunks, rachides, and leaves (53% on petioles versus 30% on rachides and 17% on leaves for *E. conferta*; 61% on petioles versus 24% on trunks and 15% on leaves for *L. longicalycata*). Moreover, the percentage of overlapping fungi found in all the tissues examined of *L. longicalycata* were only 0.3%, while 69.7% of them were only found on the petioles (versus 8.9% only found on trunks and 8.9% only found on leaves) [249]. Although only preliminary results have been retrieved, a similar pattern of petioles supporting the greatest number of fungal records and diversity were also found for the peat swamp palms *Metroxylon sagu* and *Nenga pumila* [669].

Palm petioles have long been recognised as an ideal substratum for the development of a wide diversity of fungi and their structure has been identified as a crucial factor in establishing an intimate fungus–host relationship. This fungus–host relationship was first mentioned by Mercado-Sierra [73] for the rotten and large sheathing petioles of *Roystonea regia* and later discussed for other Cuban palms [329,352]. The petioles of *R. regia* are very long (1.5–2 m) and wide. Thus, upon decomposition, the adequate surface available for the development of hyphomycetes is very large, and much larger than that of other plants, which are, therefore, subjected to a higher level of competition than palm trees [329]. Furthermore, the diversity of taxa was also considered remarkable. Mercado-Sierra [73] hypothesised that this should be related to the very long period of active leaf growth in *R. regia*, which allows a high concentration of nutrients that can be used by the fungi that inhabit its tissues.

Some studies have also suggested that the morphological and anatomical structures of palms can affect the composition and appearance of the taxa that colonise palm fronds. For instance, Hyde and Cannon [212] reported that the heavily lignified and robust arrangement of palm vascular bundles seems to affect the development and final appearance of some fungal fructifications. These fructifications tend to be elongated, so that they fill the tissue between the veins rather than growing over or through them. They are often erumpent or inserted between the outer layers of the host tissue. The more deeply immersed ascomata can often be seen developing between vascular bundles and having their walls distorted by them [212]. The hardness and arrangement of the veins in palm tissues seem to also influence the taxa that use them, which may reveal some degree of tissue-specificity. This has been observed in fungi that cause tar spots on palms. For example, relatively few *Phyllachora* species are found on *Arecaceae* when compared with other host families, and the species that do occur are limited to tissues with low levels of structural components and small amounts of lignification, such as the leaf blade [212].

The anatomical structure of palm tissues has also been reported as the main factor that determines the greater abundance and diversity of fungal assemblages on petioles, trunks, and rachides when compared with leaves [13,14,16,248,249,669]. While leaves contain mainly thin-walled, starch-rich parenchymatous cells, petioles have more thick-walled sclerenchymatous cells with associated vascular bundles [773], which can take up water and retain moisture for a longer time. Thus, thicker cell walls can yield more nutrients, namely cellulose and lignin, for sustained fungal growth. These anatomical differences between palm tissues are likely the reason why tissue-specificity has been widely suggested in palm fungal communities. The repeated occurrence of certain fungi on different types of tissue may result from different nutritional requirements of fungi or their ability to utilize different substrata due to the production of specific enzymes [13,14,249]. In addition, palm petioles are structurally more robust, with more concentrated supportive tissue than leaves, and do not decompose as quickly [773], thus allowing time for a more complex fungal community to form and a succession of different fungi to develop [10].

Many other issues can affect the composition of palm fungal communities, which will therefore also have important implications for fungal estimates. These include, for instance, fungal succession and the existence of different microhabitats, as well as fungal co-occurrence patterns and geography. Fungal succession on palms have been studied on *L. chinensis* and *Phoenix hanceana* from Hong Kong [14,634]. These studies have shown that there is a sequential order in which fungi appear on substrata as they decay, so that different fungal communities are established on different tissues during the decomposition process, with certain species associated exclusively or primarily with certain palm tissues. These results are in line with the evidence that some of the early colonisers of palm substrata are derived from endophytes and, therefore, likely to be host-specific [764]. In fact, as Hyde et al. [718] stated, if “different fungi colonise substrata at different stages of decay, this has important implications for fungi numbers”. Similarly, studies on both terrestrial [16] and peat swamp [248,249] palms have shown that palm parts exposed under different microhabitat conditions, such as dry and wet/submerged palm material, showed differences in their fungal communities and, therefore, it is likely that the existence of these different conditions also influence the fungal diversity and the suggested taxa specificity. In addition, the specificity observed can also be influenced by the existence of certain biotic relationships, such as competition, which can inhibit the establishment of certain species and may enhance the co-occurrence of others. This has recently been discussed for fungal communities on *N. fruticans* [675]. Likewise, studies on saprobic and endophytic palm fungi have provided an indication that site-specific factors and geographical distance may be important in shaping fungal assemblages. In fact, if the same host taxa occurring in different countries support similar or different fungal assemblages, this will have important implications for fungal estimates.

### 4.3. Palm Fungi as Good Biogeographical Indicators

Palm fungal communities have shown distinct patterns in relation to their collection site and geography, which in some cases seems to strongly affect their qualitative and quantitative composition. For instance, Yanna et al. [13] showed that fungal species composition was significantly affected by the site of collection. There were few taxa common (5 to 16%) to palms from different sites, insomuch that distinct fungal communities were observed on samples from Australia, Brunei, and Hong Kong. In addition, the fungal composition on palms from either different or the same genera at different sites of collection were less coherent than those from the same sites [13]. Taylor et al. [12,215] have also shown variation between geographically separated communities of endophytic fungi in *Trachycarpus fortunei*, as well as saprobes on other palm hosts.

The close association of palm fungi with palm hosts suggests that they are good biogeographical indicators. Thus, they are a particularly important group of fungi for studying the biogeographical distribution of fungi, which is a challenging task in fungal biodiversity surveys. In fact, studies likely to provide good biogeographical data are those involving fungi that coevolved or are intimately associated with their hosts [774]. This is well documented in palm fungal communities, especially by the number of host-specific fungi estimated for palm hosts [10]. In addition, some palm fungi recorded as saprobes have also been recovered as endophytes, which emphasises their close relationship with palm hosts [215].

Climate has proved to be an influential factor affecting the distribution of fungi associated with palm trees. Taylor et al. [12] reported different assemblages of fungi associated with palms in temperate regions as compared with those in tropical regions, with differences being more related to climatic influences than to the hosts sampled. In temperate regions, the dominant tropical palm mycota were replaced by more ubiquitous, plurivorous ascomycetes, in addition to fungi of different groups, such as coelomycetes. The tropical palm *Archontophoenix alexandrae* presented a largely distinct palmicolous mycota within its natural biogeographical range, when compared with the taxa recovered outside of the palm’s natural habitat. An assemblage depauperated in typical palmicolous taxa, but with representatives of widespread tropical taxa of a more plurivorous nature, was recovered from *A. alexandrae* planted outside its natural habitat [12].

Fröhlich and Hyde [6] also observed that the differences and similarities between the palm mycota recovered from Australia, Brunei, and Hong Kong are likely to be chiefly influenced by three factors, namely past and present biogeography, host distribution, and climate. According to Fröhlich and Hyde [6], while biogeography and host distribution seems to have a dominant effect on the distribution of fungi at the genus level, climate seems to be more important in determining the distribution of species. Distribution of fungi in the Old World Tropics followed patterns consistent with climate, rather than past and present biogeography and host distribution. Thus, palmicolous ascomycete assemblages from Hong Kong and Australia were found more similar despite being in different hemispheres [6]. Yanna et al. [13] and Taylor and Hyde [8] also noted that geographical distribution significantly affects palm fungal communities, regardless of the host.

The data available for palm fungi biogeography are incomplete and fragmentary [12]. However, some patterns were noted by Fröhlich and Hyde [6], mostly from the collection of palm fungi in four countries, viz. Australia, Brunei, Ecuador, and Hong Kong. The typical palm fungi found in the tropics, such as *Linocarpon*, *Astrosphaeriella*, *Oxydothis*, *Anthostomella*, *Arecomyces*, *Lophiostoma*, and *Capsulospora*, seems to have the same pantropical distribution as their hosts. Thus, the same genera, or even species, have been recorded on both sides of the equator, in both the Old and New World Tropics. Fröhlich and Hyde [6] noted no significant variation in species richness of the different tropical countries studied. However, the relative abundance of the most common species was different in the New and Old World Tropics. For instance, species of *Arecomyces* are more frequently found in Ecuador, while species of *Oxydothis* are more frequently found in Southeast Asia and Australia.

## 5. Why Study Palm Fungi? Biodiversity Estimates and Their Significance

In the wealth of data obtained from the extensive investigation on palm fungi, a diverse and abundant assemblage of host- and tissue-specific fungi was found (see Section 4). This certainly accounts for the high fungal diversity recorded on palms, and subsequently accounts for the largely unknown number of fungal species estimated. Furthermore, it has been shown that many other important factors in the estimation of fungal diversity determine the occurrence of distinct palm fungal communities, including the existence of different microhabitats, site of collection, and fungal succession. However, while palm fungi appear to be an important source for the description of many of the unknown fungal taxa, the extent of their contribution is yet to be determined. Considering the figures previously overviewed, an updated estimate of the number of fungal taxa occurring on palms worldwide is presented and discussed here, and its impact on the fungal biodiversity estimates is also noted.

Several different benchmarks can be used to assess estimates of fungal diversity on palm trees. In addition, it can be assumed that their mean value may be closer to true biological reality, since it will accommodate and integrate all those differences mentioned in the literature. One might wonder why it would be important to accommodate these differences and the answer is basically related to the remarkable extent of latitude in which palm trees can thrive and exploit ecosystems. Palms occupy a great diversity of habitats, from tropical rainforests to deserts. This reflects their impressive adaptability to diverse climatic conditions, which is easily observed in the abundance of palms in temperate regions, although the overwhelming majority are native to tropical climates [5]. Considering that palm fungi are good biogeographical indicators, due to their close association with palm hosts, their communities can provide important biogeographical data, since they comprise fungi that coevolved or are closely associated with palms [12]. Thus, it is to be expected that differences in palm fungal communities, expressed in both their qualitative and quantitative composition, will be observed when accessing the fungal composition of palms that occupy extremely distinct habitats, such as those found in temperate and tropical ecosystems.

Considering the most recent literature available, palms comprise around 2,600 species in 181 genera [1]. As previously mentioned, after years of research into palm fungi, Hyde [762,763] estimated that there were approximately three pathogens, ten saprobes, and one hundred endophytes for each species of palm. Thus, the number of ca. 113 fungal taxa developing on each palm species is used here to predict the number of fungal species expected to occur on palms worldwide.

Benchmark I—*Plant:fungus ratios in tropical palms*. Hyde [762,763] considered that 25% of the fungi that occur on palms would be host-specific, which means that 28 specific fungal taxa are likely to be associated with each palm species. A plant:fungus ratio of 1:28 would imply the existence of 67,600 species of palm fungi worldwide (Table 12). Later, the above ratio was revised upwards, and Fröhlich and Hyde [10] predicted that 33 specific fungal taxa are likely to be associated with each palm species. A plant:fungus ratio of 1:33 would imply the existence of 85,800 species of palm fungi worldwide (Table 12). Naturally, these figures, with an average value of 76,700 species of fungi on palms, are a reference for palms inhabiting tropical regions, where fungal diversity is expected to be higher compared with temperate regions. As has long been observed, plant:fungus ratios are expected to vary depending on the geographical location [10,727]. Therefore, it is expected that the plant:fungus ratio in palms native to temperate regions will be lower, as the fungal communities of temperate palms tend to be less diverse than their tropical counterparts.

Benchmark II—*Plant:fungus ratios in temperate palms*. Hawksworth and Lücking [745], while revisiting estimates of fungal diversity on Earth, estimated that plant:fungus ratios range from 1:8 to 1:19.1, with an average value of 1:9.8, a considerably higher ratio than the conservative 1:6 estimated in 1991 [727]. Interestingly, this new estimated ratio of 9.8 unique fungal species to vascular plants is based on data obtained through field surveys and molecular approaches. Thus, this figure naturally includes much more information on fungal diversity than studies that are based solely on field surveys. In fact, field surveys are biased towards recording certain groups of fungi, while others remain underexplored or even undetected. Furthermore, most of the studies considered by Hawksworth and Lücking [745] were long-term investigations based on collections of fungi from temperate regions. For example, studies on Esher Common (Surrey, England, UK), the site most investigated by field mycologists in the world, have produced a plant:fungus ratio of 1:8, which is remarkably close to the average value of 1:9.8. In this sense, the plant:fungus ratio of 1:9.8 can be seen as a more appropriate value for estimating fungal diversity in temperate vascular plants. Considering that this plant:fungus ratio is accurate for temperate palm trees, this would imply the existence of 25,480 species of palm fungi worldwide (Table 12). Although the above approach represents an interpolation of the studies by Hawksworth and Lücking [745], studies on palms can also be used to assess the plant:fungus ratio in temperate palms. Taylor et al. [12] studied the biogeographical distribution of microfungi associated with palms from tropical and temperate habitats and estimated a number of potentially host-specific fungi ranging from three to thirteen species. As expected, palms from tropical regions showed a higher number of host-specific fungi, namely thirteen for *Archontophoenix alexandrae* and ten for *Cocos nucifera*, than the three specific fungi found on *Chamaerops humilis*, which is native to temperate regions. The average plant:fungus ratio of 1:8.7 interpolated from the data obtained by Taylor et al. [12] is lower than those suggested for tropical palm hosts (1:26–33) [10]. However, it is remarkably closer to the number of specific fungi estimated for hosts in temperate regions (1:8) [745]. Furthermore, the plant:fungus ratio estimated by Taylor et al. [12] may be highly influenced by the data obtained for *Chamaerops humilis*, as well as by the experimental set-up. This included palm hosts outside their natural geographic range, which will naturally influence their fungal composition and may be the reason why tropical palms showed plant:fungus ratios more typical of temperate climate hosts. In this sense, a plant:fungus ratio of 1:8.7 is considered here to be an adequate approximation for temperate palms and would imply the existence of 22,620 species of palm fungi worldwide (Table 12). Thus, an average value of 24,050 species of palm fungi can be assumed for palms inhabiting temperate regions.

Benchmark III—*Plant:fungus ratios in palms inhabiting highly specialised habitats*. Considering the previous extensive discussion on host-specificity in palm fungi, it appears that some palm species, particularly those inhabiting exceptionally unique and diverse habitats, may have a relatively higher percentage of host-specificity than those 25% estimated by Hyde [762,763]. Studies on peat swamp palms have revealed exceptionally diverse and distinct communities recorded on palms growing in close proximity, with the description of several new species. Only less than 1%, 2%, and 6% overlap in fungal species was observed between four, three, and two, respectively, of the peat swamp palms investigated [669] (Figure 2). Although some collections can be considered as preliminary results, the overlap between fungal communities is incredibly low. In turn, this may reflect a higher plant:fungus ratio than that considered for typically tropical terrestrial palms, which probably results in a high number of host-specific fungi. Similarly, a host-specificity of ca. 30%, higher than the 25% suggested by Hyde [762,763], has been estimated here for *Nypa fruticans*, a mangrove palm that inhabits an exceptionally unique ecosystem. This percentage of host-specificity was calculated taking it to account that 42 of the 142 fungal species recorded on this palm are host-specific (Table 6). Future collections, including collections from mangrove formations in other countries, may reveal an even higher percentage of host-specificity, as new species inhabiting *N. fruticans* tissues are continuously being described, e.g., ref. [9]. It seems reasonable to consider that 25% of host-specificity may be a conservative estimate for palms inhabiting highly specialised habitats. Using a plant:fungus ratio of 1:42 as an illustrative figure of current knowledge about fungal diversity on *N. fruticans*, this would imply the existence of 109,200 species of palm fungi worldwide (Table 12). Hawksworth [727] examined the number of fungi recorded associated with vascular plants in the British Isles and considered that one third of the fungal records could be considered as not host-specific, resulting in 67% host-specificity. It is not surprising that palms show lower percentages of host-specificity than that considered by Hawksworth [727] for a temperate region. As May [775] suggested, fungi, like insects, may be more generalised with regard to hosts in tropical regions due to a greater diversity of tree species, which results in lower percentages of host-specificity. As tree diversity increases, individuals of a particular species become more sparsely distributed, which probably exerts a selective pressure on fungi to become less specialised in their host requirements [775]. Even so, although tropical fungi may tend to be less host-specific than their temperate counterparts, the extraordinarily rich mycota of tropical hosts are likely to ensure higher plant:fungus ratios [10]. Thus, considering that ca. 30% of host-specificity in *N. fruticans* is likely to be higher as research continues to reveal new species (especially with the introduction of DNA sequence-based identifications), and that the worldwide prediction of fungal diversity is based on two/thirds host-specificity, it can be considered that host-specificity in palms inhabiting highly specialised habitats may have an intermediate value. It can be assumed that an average host-specificity value of ca. 49% occurs in palm trees inhabiting highly specialised habitats. In these cases, a plant:fungus ratio of 1:55 can be hypothesised, which would imply the existence of 143,000 species of palm fungi worldwide (Table 12). Thus, an average value of 126,100 species of palm fungi can be assumed for palms inhabiting highly specialised habitats.

**Table 12:** Estimates of the total number of species of palm fungi in the world derived by different methods (see Section 5 for further explanation).

Benchmark	Basis	Reference and Reasoning	Plant:Fungus Ratio ^1^	Estimate of Total Species Number ^2^
I	Plant:fungus ratios in tropical palms	Hyde [762,763] based on extensive work on palm fungi in Australia	1:28	67,600
		Fröhlich and Hyde [10] based on survey of fungi associated with six *Licuala* palms in Australia and Brunei Darussalam	1:33	85,800
II	Plant:fungus ratios in temperate palms	Hawksworth and Lücking [745] based on long-term investigations mainly on fungal collections from temperate regions	1:9.8	25,480
		Present study based on the estimates presented by Taylor et al. [12] for the number of host-specific fungi in tropical and temperate palms growing inside and outside their natural geographic range	1:8.7	22,620
III	Plant:fungus ratios in palms inhabiting highly specialised habitats	Present study considering that 42 of the 142 fungal species recorded on *Nypa fruticans* are likely to be host-specific	1:42	109,200
		Present study considering that palms inhabiting highly specialised habitats may have a higher percentage of host-specific fungi than typical tropical palms (25%) and be closer to the percentage of host-specificity estimated for temperate hosts (63%)	1:55	143,000
Mean I–III				75,617

^1^ Plant:fungus ratio 1:52 for benchmark III was calculated considering the estimates presented by Hyde [762,763] for the number of fungal taxa (ca. 113) expected to develop on each palm species. ^2^ The estimate of the total number of fungal species was calculated considering that, according to the most recent literature available, palm trees comprise around 2600 species [1].

Based on the above-mentioned estimates, an average of benchmarks I to III yields a figure of 75,617 species of palm fungi worldwide (Table 12). However, some considerations should be made before further predictions, considering the estimation of almost 76,000 species of fungi on palms worldwide, which is remarkably close to the previous prediction of 73,000 by Taylor and Hyde [8]. The benchmarks presented are not comparable and represent different means of evaluating or predicting the same information, i.e., plant:fungus ratios in palm trees, given that plant:fungus ratios are one of the most important factors used in estimating global numbers of fungi [764]. A wide range of variation has been considered, which attempts to mimic the wide variation in habitats exploited by palms and the intimate relationship that palm fungi establish with their hosts. However, the figure of 76,000 species of fungi on palms worldwide is considered conservative and its accuracy is yet to be determined. Some considerations are discussed below.

*An estimate based on an unweighted average*. A determining factor for this scenario is the fact that all the benchmarks considered have the same weight when calculating the average value. However, most palm trees are native to tropical and subtropical regions, so it can be predicted that a plant:fungus ratio calculated on tropical palms (benchmarks I and III) may reveal a more realistic scenario than the one calculated on temperate palms (benchmark II). In fact, only about 130 of the 2600 palm species (ca. 5%) occur naturally beyond the tropical latitudes and would have a plant:fungus ratio like those calculated for temperate hosts [776]. Moreover, more than 90% of *Arecaceae* species diversity is restricted to tropical rainforests and part of the remaining 10% inhabit seasonal tropical and subtropical vegetation [777].

*The overlooked fungal biodiversity*. In most studies on palm fungi, particularly those that estimate plant:fungus ratios, the biodiversity of several groups of fungi are omitted from the surveys. These include fungi growing on tissues that are usually not examined, such as fungi from below-ground or reproductive organs, as well as lichen-forming fungi, whose occurrence is almost unknown on palm trees. In addition, many other species of fungi are often not recovered from samples, such as fastidious fungi, which can only be isolated using selective media (for detailed discussion on biodiversity of fungi omitted in such studies see [10]). Similarly, most studies are often biased towards the isolation or collection of ascomycetes or asexual morphs. As a consequence, certain taxonomic groups are overlooked, such as basidiomycetes, which are clearly poorly studied on palms (see Section 3).

*The lack of data on certain palm fungal communities*. Although considered conservative, the almost 76,000 species of fungi on palms worldwide may not be far from the truth, since the downward factors can be accommodated in benchmark III, which predicts a much higher plant:fungus ratio for some palms that inhabit hyperdiverse tropical ecosystems. However, the contribution of this benchmark is less detailed and supported by actual data than benchmarks I and II. Therefore, its contribution as a fine-tuning factor to the accuracy of the estimate may not be as reliable as the contribution of benchmarks I and II. This exceptionally high plant:fungus ratio is not expected to be found in most palms, as it is likely to be influenced by the unique abiotic conditions of the highly specialised habitats that are colonised by few palm species.

*The lack of molecular-based studies*. The vast majority of ecological studies on palm fungi have been based on morphological analyses. Since morphological characters are known to be an inadequate approach to identifying fungi due to phenotypic plasticity, the true diversity of documented palm fungal communities is likely to be underestimated. In addition, to date, almost no study has explored the diversity of palm fungi using palm samples for studies based on DNA metabarcoding technology via HTS. For instance, recent studies on the endophyte communities inhabiting the leaflets of mule palms (×*Butiagrus nabonnandii*), sampled using culture-dependent (CD) and culture-independent (CI) methods, have shown a small overlap in endophyte composition, with CI methods providing a higher estimate of species richness and composition [778]. Since palm fungal communities have proven to be extremely diverse, the assessment of environmental DNA in palm samples would probably give a more accurate idea of their true diversity and how far from the truth current predictions are. Likewise, given their diverse nature, palm fungal communities assessed using DNA metabarcoding technology could provide important new insights into the current discussion on how to formally describe “dark taxa”.

Predicting fungal diversity in palms is of great significance for estimates of the total number of fungi worldwide. In the present review, it is conservatively estimated that ca. 76,000 species of fungi can be found on palm trees worldwide, of which only just over 3% (ca. 2500 species) have been documented. This results in a total of ca. 97% of palm fungi awaiting to be documented. Considering that ca. 75% of all fungi collected on palms are new to science, this means that prioritising palm trees as host plants for fungal collections could reveal more than 55,000 new taxa to science. Therefore, given that the most currently accepted estimate of fungal species richness is between 2.2 and 3.8 million, ca. 2.5 to 1.5%, respectively, of the world’s unknown mycota could potentially be found on palm hosts.

## 6. Conclusions and Future Perspectives

Research carried out over the last 30 years suggests that *Arecaceae* hosts can be regarded as model plants for the study of fungal biodiversity. Palm tissues seem to support a vast and diverse mycobiota that can address several questions in biodiversity studies, which can be pointed out as actual fungal biodiversity challenges. Studies in Southeast Asia and Australasia have shown that there is a hyperdiverse group of fungi, referred to as palm fungi, consistently associated with palm trees in the tropics. A number of ecological issues were assessed in palm fungi, which are critical to the study of fungal communities and their biological patterns in ecosystems. These studies have emphasised the intimate relationship of palm fungi with palm hosts, insomuch as they are considered to be good biogeographical indicators, playing a key role in biodiversity surveys.

Palm fungi are considered a unique group of fungi, since many genera found to be associated with palms are host-specific or are rarely found associated with other plants. This host-specificity is far from being fully understood. Studies have revealed that it may be associated with the ecology of these microorganisms and their close association with palm hosts. In fact, some palm fungi recorded as saprobes are often recovered as endophytes, which can justify the high plant:fungus ratios estimated in *Arecaceae* hosts. In turn, this surely accounts for the remarkable number of new species to science that are continuously being described from palms.

The currently accepted estimate of the world’s mycota is between 2.2 and 3.8 million fungal species, yet less than 10% of them have been named so far. For this reason, the question “where are the missing fungi?” has often been asked and has motivated the persistent search for new fungal species. Evidence gained from the extensive investigation on palm fungi undoubtedly indicates that many of the missing fungi can be found on palms. In the present review, it has been conservatively estimated that more than 55,000 new taxa are expected to be found in palm collections. This means that approximately 1.5 to 2.5% of the world’s unknown mycota could potentially be found on palm hosts. Although host-specificity can be an important factor in estimating the number of palm fungal species worldwide, it is likely that conclusions are being drawn from data that are somewhat biased towards fungi, hosts, and substrata that are of human interest. This has been well illustrated in studies on palm fungi, which have largely focused on palms of international economic interest.

The estimates provided here highlight that palm fungi are an understudied assemblage. There is an enormous wealth of undiscovered and untapped palm fungi that could hold substantial potential for mankind. Therefore, the search for the undescribed palm fungi (and fungi in general) and the study of their diversity patterns are of the utmost importance in biodiversity studies. These studies have incredible economic potential in discovering microorganisms with new biotechnological and industrial uses. However, only a more complete inventory of these microorganisms will make it possible to preserve a representative collection for future research, society, and prosperity. Furthermore, this will only be possible with the development of appropriate protocols and methods to detect and understand this diversity, which will largely depend on the use of molecular data.

DNA sequence-based studies have revealed numerous additional cryptic taxa in well-known and established species and genera, suggesting that fungal biodiversity estimates may be highly underestimated. Most studies on palm fungi have been based on morphological analyses. As a result, most of the 2500 species of palm fungi have no associated molecular data. In order to successfully study and understand these fungi, a major investment is needed in their re-collection and epitypification. Only phylogenetic studies will be able to clarify the taxonomic structure of palm fungi and fill the current gaps in their knowledge. In addition, molecular analyses, including DNA metabarcoding, could provide missing links to palm fungal communities and therefore help to understand their population dynamics, such as host-specificity and biogeographical distribution.

## Data Availability

Study does not involve any huge data acquisition and the corresponding authors may be contacted for further assistance of the subjects discussed.
